# Biogeographic Overview of Ulmaceae: Diversity, Distribution, Ecological Preferences, and Conservation Status

**DOI:** 10.3390/plants10061111

**Published:** 2021-05-31

**Authors:** Yann Fragnière, Yi-Gang Song, Laurence Fazan, Steven R. Manchester, Giuseppe Garfì, Gregor Kozlowski

**Affiliations:** 1 Department of Biology and Botanic Garden, University of Fribourg, Chemin du Musée 10, CH-1700 Fribourg, Switzerland; yann.fragniere@unifr.ch (Y.F.); laurence.fazan@unifr.ch (L.F.); 2 Eastern China Conservation Centre for Wild Endangered Plant Resources, Shanghai Chenshan Botanical Garden, 3888 Chenhua Road, Songjiang, Shanghai 201602, China; ygsong@cemps.ac.cn; 3 Florida Museum of Natural History, University of Florida, 1659 Museum Rd, Gainesville, FL 32611, USA; steven@flmnh.ufl.edu; 4 Institute of Biosciences and BioResources—National Research Council, Corso Calatafimi 414, 90129 Palermo, Italy; giuseppe.garfi@ibbr.cnr.it; 5 Natural History Museum Fribourg, Chemin du Musée 6, CH-1700 Fribourg, Switzerland

**Keywords:** climatic niche, diversity centers, elm family, Köppen–Geiger climate classification, latitudinal diversity gradient, relict trees

## Abstract

The elm family (Ulmaceae) is a woody plant group with important scientific, societal, and economic value. We aim to present the first biogeographic synthesis investigating the global diversity, distribution, ecological preferences, and the conservation status of Ulmaceae. A literature review was performed to explore the available data for all extant species. Our study made it possible to map the actual global distribution of Ulmaceae with high precision, and to elucidate the centers of diversity, located mainly in China and in the southeastern USA. A detailed comparative analysis of the macroclimatic niche for each species was produced, which shows the general biogeographic pattern of the family and pinpoints the outlier species. The results corroborate recent molecular analyses and support the division of Ulmaceae into two taxonomically, biogeographically, and ecologically well-differentiated groups: the so-called temperate clade with 4 genera and 43 species and the tropical clade with 3 genera and 13 species. The elm family is often described as a typical temperate plant group, however the diversity peak of all Ulmaceae is located in the subtropical zone, and a non-negligible part of the family is exclusively distributed in the tropics. We also noticed that a high proportion of Ulmaceae is linked to humid macro- or microhabitats. Finally, we highlighted that nearly 25% of all Ulmaceae are threatened. Fieldwork, conservation efforts, and research activities are still necessary for this family, particularly for the tropical members and the most endangered species.

## 1. Introduction

Although ranking among the smallest families in the plant kingdom in terms of the number of species, the elm family (Ulmaceae) has important scientific, economic, societal, and conservation value [1,2]. Ulmaceae is an ancient and exclusively woody plant group consisting of deciduous, rarely evergreen trees and shrubs [3,4,5]. It is an extremely interesting plant family with respect to different scientific issues, such as paleobotany, biogeography, systematics, plant evolution, or species diversification. However, though considered a relatively well known plant group, many of its representatives, especially from the tropical regions, still remain insufficiently investigated, e.g., [6,7].

Ulmaceae includes many relict trees. Fossil records date the origin of the family to the early Cenozoic Era. By the early Paleocene, members of the elm family were already widespread throughout the entire Northern Hemisphere [8,9], but the oldest confirmed records of extant genera, such as *Ulmus* and *Zelkova*, are from the Eocene. At least one widespread genus went extinct, i.e., *Cedrelospermum*, from the Paleogene and Neogene of Europe, Asia, and North America [10,11]. Other genera, common in the past at the continental scale, persist at present in disjunct (e.g., *Zelkova*, growing in Eastern Asia and South-Western Eurasia) [1] or in restricted distribution areas (e.g., *Hemiptelea*, thriving in several localities of Korea and eastern China) [12]. The oldest fossils of living genera consist of leaves and fruits of *Ulmus* referred to the early Eocene of China (ca. 50 Mya) and the middle to late Eocene of North America [13].

The systematics of elm family has had a very controversial story. It was taxonomically described for the first time in 1815 by de Mirbel [14] and contained at that time only two genera, *Ulmus* and *Celtis*. Subsequently, for nearly 150 years, the family was commonly divided into two subgroups associated with each of these original genera [8]. Until the late 1990s, two subfamilies of Ulmaceae were recognized, the Ulmoideae and the Celtidoideae, often denominated as “ulmoids” and “celtoids,” respectively [15], though at the end of the 1960s Grudzinskaya [16] had proposed distinguishing two different families within the family Ulmaceae, the Ulmaceae s.s. and the Celtidaceae. At that time, the number of genera included in the elm family ranged between 15 and 18 [8,9]. The clarification of the taxonomic division of this group came with the molecular phylogenies performed on all closely related urticoid families of the order Rosales, mainly on Cannabaceae. The modern circumscription of Cannabaceae resulted in the integration of the majority of celtoids into this family (e.g., *Celtis*, *Pteroceltis*, *Trema*, *Aphananthe*, and *Lozanella*) and their exclusion from Ulmaceae [15,17]. Furthermore, the genus *Ampelocera*, treated previously as a member of Celtidoideae [8,18], was recognized as an ulmoid taxon [6,15,19].

Modern treatments based on molecular phylogenies therefore clearly separate Cannabaceae and Ulmaceae [15,18,19,20,21]. Moreover, Cannabaceae is a sister family to Moraceae and Urticaceae and thus is not the closest taxon of Ulmaceae within the urticalean rosids [17,20,22]. Moreover, recent molecular studies [23,24] divide the Ulmaceae into two distinct taxonomic and biogeographic groups: the temperate clade (including *Ulmus*, *Zelkova*, *Planera,* and *Hemiptelea*) and the tropical clade (including *Ampelocera*, *Phyllostylon,* and *Holoptelea*).

Ulmaceae possess very distinctive fruit structures (and corresponding dispersal mechanisms), which provide interesting elements to outline the evolutionary pathways within the family [16], in addition to being extremely important for the identification of extant and extinct genera and species [8]. The asymmetric akene-type, unwinged fruit of *Zelkova* is suggested as being the most primitive fruit-type within the family. At the opposite, the genera *Ulmus*, *Hemiptelea*, *Holoptelea,* and *Phyllostylon* have the most evolved winged fruits and are thus clearly wind-dispersed [9]. However, the dispersal mechanisms of *Hemiptelea* need more investigation, since its small asymmetric fruits possess a wing-like appendix only on one side of the fruit [3], and for this reason it can be referred to as an intermediate step in the evolutionary pathway of the family. *Planera* have fleshy protuberances, and since they grow mainly along water courses, the tree is probably water dispersed [9]. The members of the neotropical *Ampelocera* possess ellipsoid or even pyriform drupes, which in certain species can be relatively large and colored (e.g., *A. macrocarpa*) and are primarily bird-dispersed [6]. The most sophisticated, however, seems to be the dispersal mechanisms in the genus *Zelkova* due to the drupaceous and unwinged features of its individual fruits. In fact, mature fruits commonly fall with the entire twig, and the dried leaves that are still attached function as a drag-enhancing appendage, carrying the fruits away from the parent tree [25,26].

The members of Ulmaceae show a variety of breeding systems and floral types [27]. Some genera, such as *Zelkova* or *Planera*, exhibit three flower types (staminate, pistillate, and hermaphrodite flowers) on the same individual or even on the same flowering branch [27,28]. Similar to other closely related families belonging to the Urticalean rosids, the flowers of Ulmaceae have only one whorl of 4–8 green or brown perianth lobes (denominated either as sepals or tepals). Stamens usually show the same number as tepals (with the exception of *Holoptelea* and *Ampelocera* with up to 12 or 16 stamens, respectively) [27]. The superior ovary is composed of two fused carpels with two linear styles [4].

Despite the long history of Ulmaceae research, a synthesis of the diversity and biogeography of this family that takes more recent publications and the current state of knowledge into account has yet to be produced. Information dealing with spatial distributions and biodiversity is central to many fundamental questions in biogeography and conservation biology [29,30]. The distribution of different plant taxa (especially families) is basic and essential information fundamental in many studies, but syntheses at global scales are rather scarce [31,32]. Understanding global biogeography is of great importance for the effective conservation of any group of organisms, especially plant families with disjunct distribution patterns [28]. In this paper, we investigate the global diversity, distribution pattern, conservation status, and ecological preferences of the elm family. The aim of the present work is thus to provide an up-to-date synthesis. Our main objectives are to (1) present the actual global distribution of Ulmaceae with the highest possible resolution, (2) contribute to elucidating the diversity hotspots of Ulmaceae at the generic and species levels, (3) elucidate the realized (macro)climatic niche and ecological preferences of all extant Ulmaceae species, and (4) synthesize the conservation status of the elm family.

## 2. Results

### 2.1. Taxonomic Division and Species List

Based on the published taxonomic treatments and available literature (Supplementary file S1), the taxonomic division and number of species of the Ulmaceae family is given in Table 1. The elm family consists of 56 species, divided into 2 clades (13 in the tropical clade and 43 in the temperate clade) and 7 genera. In general, the information and published literature on the tropical members of the family are sparser than for the temperate species (Supplementary file S1). The following doubtful *Ulmus* species were not included: *U. chumlia*, treated as a synonym of *U. androssowii* [33,34]; *U. procera*, treated as a synonym of *U. minor*, introduced in North America [34,35]; *U. elliptica* (Caucasus), treated as a synonym of *U. glabra* [33,36]; and *U. densa* (Central Asia), treated as a synonym of *U. minor* [33,37].

### 2.2. Species and Genera Distribution

**Figure 1:**
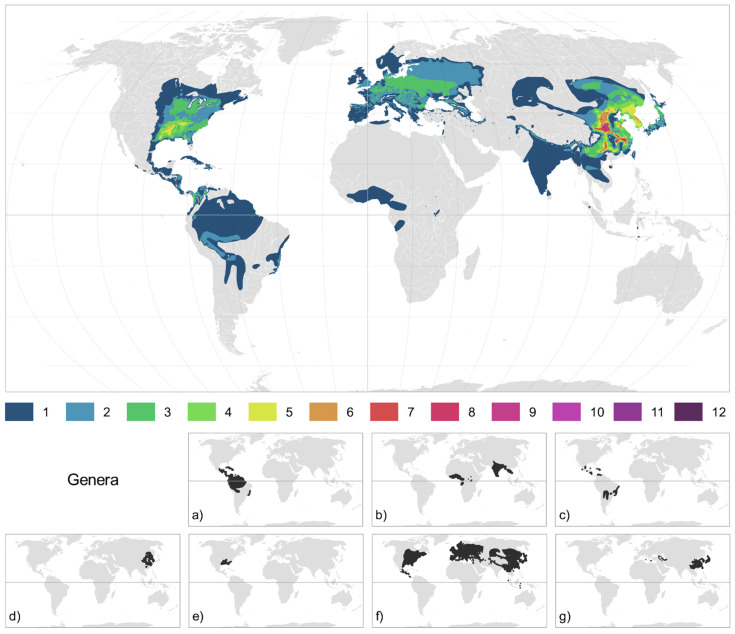
Global distribution of Ulmaceae. The color gradient shows the number of species with overlapping distribution. The small maps below indicate the global distribution of the different genera. Tropical clade: (**a**) *Ampelocera*, (**b**) *Holoptelea*, and (**c**) *Phyllostylon*; Temperate clade: (**d**) *Hemiptelea*, (**e**) *Planera*, (**f**) *Ulmus*, and (**g**) *Zelkova*.

An up-to-date global distribution map of Ulmaceae was produced, which corresponds to the most actual chorological knowledge of this family with the highest possible resolution (Figure 1). This map combines the individual distribution maps made for each species (see Methods). In many areas, the distributions of several species overlap. Several regions can be considered hotspots of Ulmaceae diversity, with numerous species co-occurring in the same area. This is especially true for Eastern Asia. China has the highest diversity worldwide, with 12 species and 3 genera. The main Chinese hotspots are in the following provinces: western Henan, Shanxi, Shaanxi, southern Anhui, western Zhejang, northern Jiangxi, and Hubei. At least three species can be found in all provinces of China (except western provinces and Hainan), in Taiwan, in North and South Korea, in Japan and in the Russian Far East. The southeastern United States is another important Ulmaceae hotspot, mainly in Arkansas (six species and two genera), Tennessee, Louisiana, Mississippi, Alabama, western Kentucky, and eastern Texas. In the majority of Central and Eastern Europe, three *Ulmus* species co-occur. In the Sub-Himalayan region, up to three *Ulmus* species can also be found together in India (Kashmir, Himachal Pradesh). In South and Central America, a maximum of three to four *Ampelocera* species occur together, mainly in eastern Colombia (Choco, Antioquia, Cordoba, and Zulia) and marginally in Brazil (Acre).

**Figure 2:**
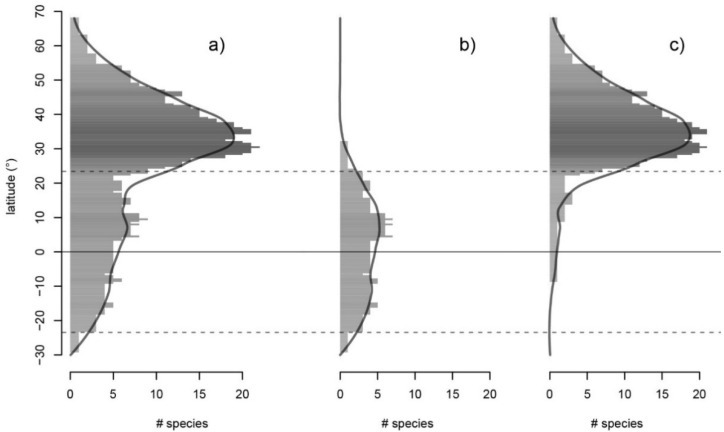
Latitudinal diversity gradient. Species richness by 0.5° latitudinal bin for (**a**) the complete Ulmaceae family, (**b**) the tropical clade and (**c**) the temperate clade. A smooth approximation is shown by the LOESS (locally estimated scatterplot smoothing) curve above the histogram. The equator is represented by the solid horizontal line, and the tropics are represented by the two dashed lines.

The two clades show very different latitudinal diversity patterns (Figure 2). The tropical clade is nearly entirely confined between the tropical lines, with a peak between 5° and 12° of N latitude. Only rare species of the temperate clade cross the Tropic of Cancer to the south. The diversity peak of the temperate clade (between 28° N and 38° N) is located in the subtropical zone. Ulmaceae extends to the south to approximately 24° S (*Phyllostylon rhamnoides* in South America) and to the north up to approximately 69° N (*Ulmus glabra* in Europe).

### 2.3. Species Macroclimatic Niche and Ecological Preferences

**Figure 3:**
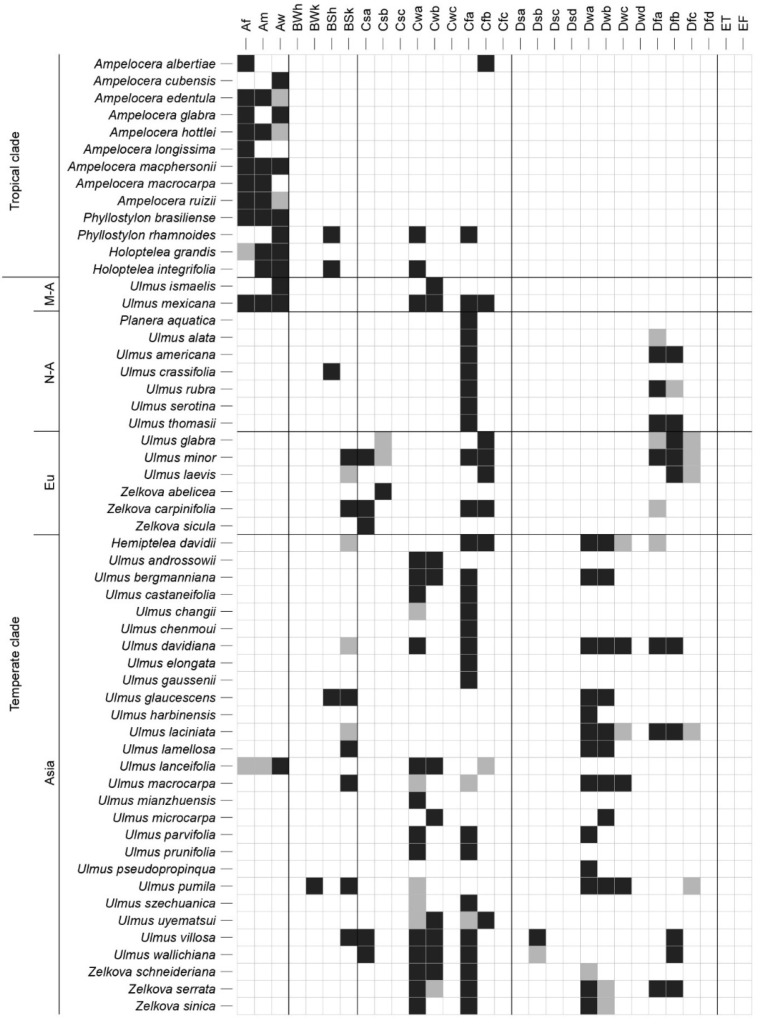
Realized (macro)climatic niches for each species of the Ulmaceae family, according to the Köppen–Geiger climate classification. A black square indicates that a species is largely distributed in the corresponding climate, and a grey square indicates that the species is only marginally distributed in the corresponding climate. The species are presented in the following order from top to bottom: tropical clade and temperate clade, further divided in Mesoamerica (M-A), North America (N-A), Europe (Eu), and Asia. The upper abbreviations indicate the type of climate: tropical rainforest climate (Af), tropical monsoon climate (Am), tropical savanna climate (Aw), arid climate (BW; h—hot, k—cold), semiarid (steppe) climate (BS; h—hot, k—cold), Mediterranean climate (Cs; a—hot summer, b—warm summer, c—cool summer), humid subtropical climate (Cfa), oceanic climate (Cfb), subpolar oceanic climate (Cfc), dry-winter humid subtropical climate (Cwa), dry-winter subtropical highland climate (Cwb), dry-winter subpolar oceanic climate (Cwc), continental climate (D; s—dry summer, w—dry winter, f—no dry season; a—hot summer, b—warm summer, c—cold summer, d—very cold winter), tundra climate (ET), and ice climate (EF).

**Figure 4:**
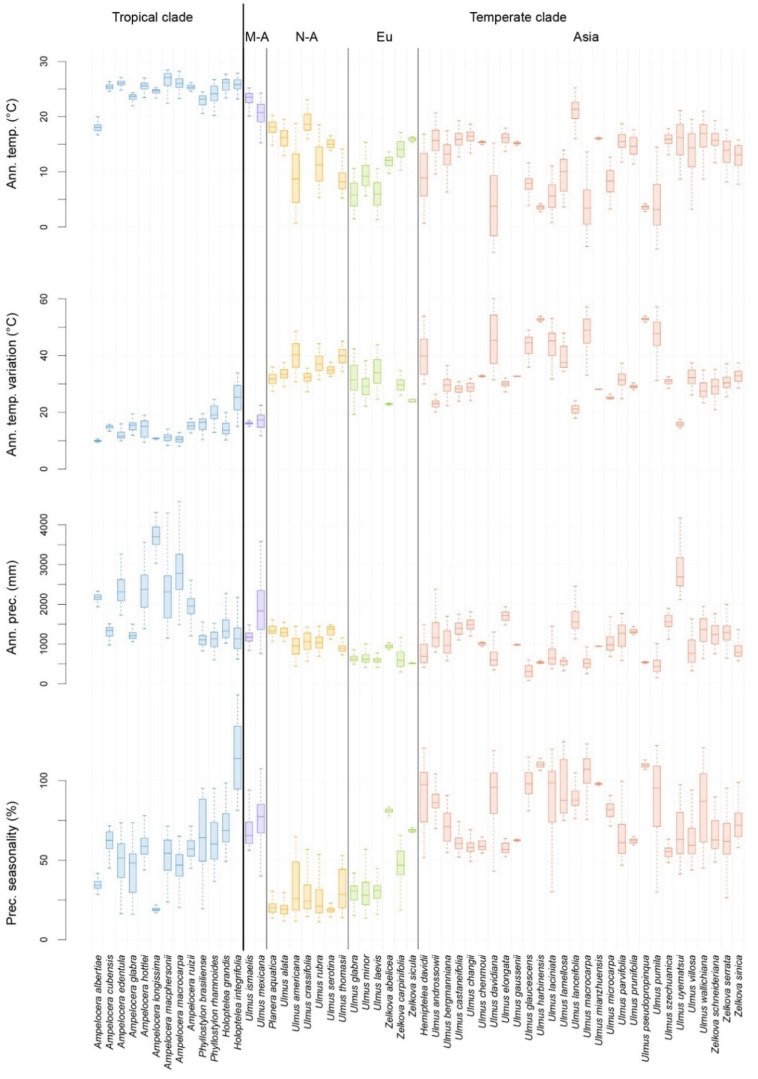
Macroclimatic preferences for each species of the Ulmaceae family according to their natural distribution and for a selection of four climatic variables: mean annual temperature (°C), mean annual temperature variation (maximum temperature of the warmest month—minimum temperature of the coldest month, °C), mean annual precipitation (mm) and precipitation seasonality (variation in monthly precipitation totals over the course of the year, %). The species are presented in the following order from left to right: tropical clade and temperate clade, further divided in Mesoamerica (M-A), North America (N-A), Europe (Eu), and Asia. These five groups are indicated by different colors.

**Figure 5:**
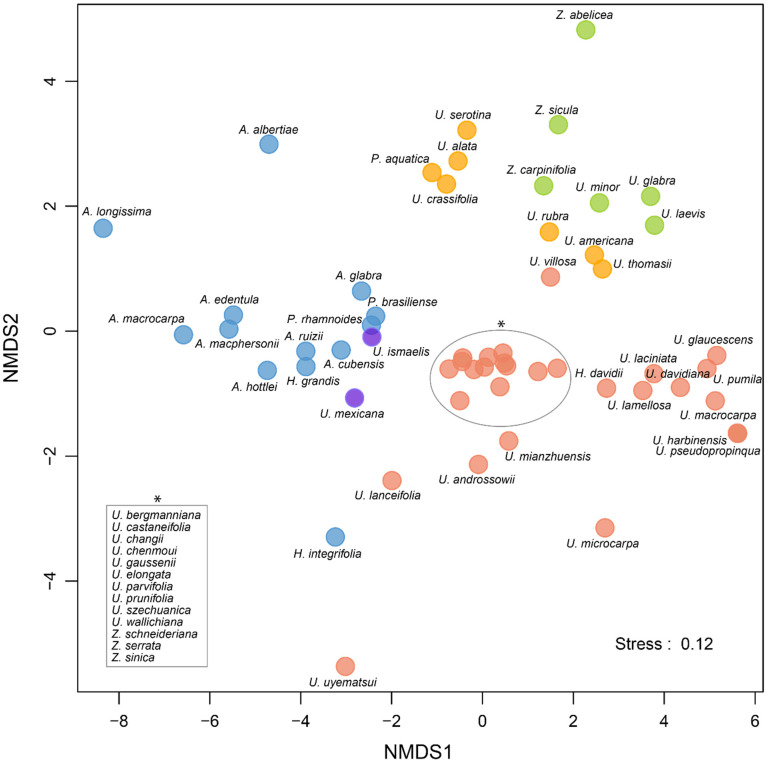
Nonmetric multidimensional scaling (NMDS) ordination plot representing the (macro)climatic similarities between species of the Ulmaceae family. A total of 19 climatic variables were included in the analysis (see Material and Methods). Species appearing close together in the plot share similar macroclimatic preferences. The species are divided into the following groups: tropical clade (blue), temperate clade divided further in Mesoamerica (purple), North America (yellow), Europe (green), and Asia (red).

A detailed overview of the realized macroclimatic niche of all the species of the Ulmaceae family is presented in Figure 3 and Figure 4. Additionally, the ordination plot (Figure 5) allows us to elucidate species that share similar macroclimatic preferences and to highlight outliers. Most of the species of the tropical clade are distributed in areas with tropical climates of (A)f—rainforest, (A)m—monsoon, and (A)w—savannah. The exception is *Ampelocera albertiae,* growing at a high elevation in the mountains in a rather temperate oceanic climate (Cfb), as well as *Phyllostylon rhamnoides* and *Holoptelea integrifolia* found in several climate types, such as BSh (semiarid hot climate), Cwa (dry-winter humid subtropical climate), and Cfa (humid subtropical climate).

The North American members of the temperate clade of Ulmaceae mainly occur in Cfa (humid subtropical climate). Among them, three species also occur in Dfa and Dfb (continental climate without dry season, with warm to hot summer). The European members are mainly in Cfb (oceanic climate), but some species are found in more Mediterranean and/or continental climates. The Asiatic members of the temperate clade are mainly typical elements of Cfa (humid subtropical climate) but are also very common in Cwa (dry-winter humid subtropical climate) and Cwb (dry-winter subtropical highland climate), as well as in Dwa and Dwb (continental climate, with dry winter and warm to hot summer). The niches of some Asiatic species (*Ulmus pumila*, *U. macrocarpa*, and *U. davidiana*) cover a large gradient of temperatures, including very cold areas, with mean annual temperatures close to or below 0 °C and sometimes with extreme annual temperature variations (Figure 4). Several species are clear outliers among the temperate clade: the Mexican and Mesoamerican *Ulmus* species, the East-Asiatic *U. lanceifolia* and *U. uyematsui* from Taiwan, and the Mediterranean *Zelkova* species.

We collected all available information on the relationship of Ulmaceae species with water and soil conditions (Table 2). Nearly 70% of species belonging to the elm family occur (obligatorily or facultatively) in wet habitats. A majority of species are typical elements of tropical humid forests (15 spp.) or are found exclusively in alluvial and riparian forests (11 spp.). Additionally, there were a relatively large number of Ulmaceae (13 spp.) that occur (not exclusively) in wet microhabitats. Finally, a significant proportion of Ulmaceae species seem to prefer rich, fertile soils.

### 2.4. Conservation Status

Thirty-eight Ulmaceae species are included to date on the IUCN Red List (i.e., 68% of the total species number). For one-third of all members of the elm family, no global assessment has been made (Table 3). Among the assessed species, two were critically endangered (IUCN category CR), four were endangered (EN), five were vulnerable (VU), and two were near threatened (NT). Thus, 34% of all assessed species and 23% of all Ulmaceae species are considered under threat. This group of threatened species includes all six *Zelkova* species, five *Ulmus* species, and two *Ampelocera* species.

## 3. Discussion

### 3.1. Diversity and Distribution

Our global synthesis corroborates recent molecular analyses [23,24] and thus strongly supports the division of Ulmaceae into two taxonomically, biogeographically, and ecologically well-differentiated groups: the so-called temperate clade with 43 species and four genera (*Hemiptelea*, *Zelkova*, *Planera,* and *Ulmus*) and the tropical clade with 13 species and three genera (*Holoptelea*, *Phyllostylon,* and *Ampelocera*). There exists an enormous discrepancy among the number of studies and thus with the exploration and understanding of the biological and evolutionary processes of the temperate species of Ulmaceae in comparison with the tropical species. Therefore, many aspects of the biology, ecology, and phylogenetic relationships of the tropical clade still need much scientific effort [3,6,7]. Furthermore, for many tropical species, in-depth fieldwork is still needed to collect sufficient data about their distribution (e.g., neotropical species with large and scattered distribution: *Phyllostylon rhamnoides* and *Ampelocera macrocarpa*) and to better understand and document their ecology.

Better scientific exploration of northern temperate Ulmaceae is closely linked with their use by humans [38,39,40]. For millennia, temperate Ulmaceae trees played an important role in rural and forested areas, being part of the traditional landscape as trees with multiple uses [36]. This is especially the case of various species of elm (*Ulmus*) and the keaki tree (*Zelkova serrata*) in Eastern Asia, which is widely used as an ornamental tree in silviculture and timber production [1]. Interestingly, many tropical species are locally commonly used for wood and tool production (e.g., *Phyllostylon brasiliensis*) and/or possess high potential for medicinal purposes (e.g., *Holoptelea*) [4,41] and thus would need an intensification of research efforts.

For similar reasons, the elm family is often described as a nearly exclusively temperate plant group, typical of the Northern Hemisphere [4,9]. Our global synthesis relativizes this assumption. First, the diversity peak of all Ulmaceae is located in the subtropical zone (between 28° and 38° N) and a non-negligible part of the family (ca. 23% of all species) is exclusively distributed in the tropics (the so-called tropical clade). Additionally, several members of the temperate clade are present or even endemic to tropical regions (e.g., *Ulmus ismaelis* and *U. mexicana* in Mexico; and *U. parvifolia*, *U. uyematsui,* or *Zelkova schneideriana* in Taiwan). One member of the temperate clade reaches the Southern Hemisphere (*Ulmus lanceifolia* distributed to the south on the Celebes and Flores islands).

One of the most famous large-scale patterns in biological diversity is the increase in the number of species from the poles to the equator, a trend that has been called the latitudinal diversity gradient (LDG) [29,42]. On a large scale, only a few plant groups do not follow this pattern, such as Poaceae [43] and gymnosperms [31]. Ulmaceae, as a whole, do not exhibit a typical LDG because they have a diversity peak between 28° and 38° of northern latitude (Figure 2). This does not seem to be an unusual pattern in small woody families, with predominantly temperate and relict species (e.g., Juglandaceae, [32]). However, the typical LDG with a peak close to the equator can be observed when only the tropical clade of Ulmaceae is taken into consideration (Figure 2b).

Interestingly, the elm family shows a very similar biogeographical pattern and distribution of diversity centers with other exclusively woody plant families that are rich in relict trees, such as the walnut family (Juglandaceae) [5,32]. Similar to Juglandaceae, the main diversity center of Ulmaceae lies in southeastern Asia (mainly in China) followed by a second center in the southeastern USA and a third in Mesoamerica and northern South America (Figure 1).

Moreover, the fossil record demonstrates prior extirpations and extinction in the history of Ulmaceae, possibly reflecting the response to prior climate change. For example, *Hemiptelea*, which is now endemic to eastern China and the Korean Peninsula, has been confirmed on the basis of its distinctive fossil fruits from the Miocene of Poland and Ukraine (see references in [44]). Although *Ulmus* is no longer native to west-coastal North America, the genus was well established and identifiable from fruits, as well as leaves, in the Eocene to Miocene of California, Oregon, Washington, and British Columbia [8,45]. *Cedrelospermum* was widespread in the Eocene of North America, Europe and Asia and extended well south into the later Cenozoic [46]. The cause of its global extinction remains uncertain.

### 3.2. Macroclimatic Niche and Ecological Preferences

Our study shows strong differentiation of the macroclimatic niche between the tropical and temperate clades of Ulmaceae (Figure 3, Figure 4 and Figure 5). Most of the species of the tropical clade are typical elements of tropical and monsoon forests or of savannah. They are thus distributed in tropical climates with a mean annual temperature generally between 20 °C and 28 °C, with very low seasonal variation and annual precipitation that are normally over 1000 mm per year and up to 4000 mm per year for some species in specific regions (e.g., *Ampelocera longissima*, *A. macphersonii,* and *A. macrocarpa*). There are several exceptions, however, with species occupying niches in temperate regions (e.g., in upland areas, *Ampelocera albertiae*) and/or semiarid hot regions (*Phyllostylon rhamnoides* and *Holoptelea integrifolia*). Interestingly, a majority of tropical Ulmaceae possess large leaves (often having entire margins), in contrast to the smaller and usually dentate leaves of temperate genera [6]. This is often interpreted as an adaptation to wet, tropical forest habitats [15]. However, due to the lack of fossil records of the disjunct genera and species of the tropical Ulmaceae clade, their origin and the history of adaptation to tropical climates are less well understood [8].

Similarly, there are also some species of the temperate clade occurring within tropical climates (not exclusively), such as the *Ulmus* species of Mexico and Mesoamerica (*U. mexicana* and *U. ismaelis*) and one species in Eastern Asia (*U. lanceifolia*). These three species are close to the tropical clade in terms of climatic preferences (Figure 4 and Figure 5). North American members of the temperate clade are mainly linked to the humid subtropical climate and in the north to a moderate and humid continental climate. Their climatic niches are generally close to those of European species, but the latter occur in slightly cooler oceanic and continental climates with less precipitation. In Europe, *Zelkova abelicea* and *Z. sicula* are the two notable exceptions among the members of the temperate clade (Figure 4 and Figure 5). They occur within a Mediterranean climate with high precipitation seasonality and dry summers. Recent detailed studies on *Z. sicula* show that this relict and narrow endemic species is isolated and restricted to the Mediterranean climate. The species survived in this region due to suitable microhabitats, but its distribution and dispersal are limited by the Mediterranean climate [47]. Our results highlight that *Zelkova* species found in Mediterranean climates are exceptions among the Ulmaceae.

In Asia, the numerous species of the temperate clade occupy diverse climatic niches with a large gradient of climatic preferences. There is one constant, however, namely, the high precipitation seasonality due to the East Asian monsoon regime, with dry winters. Some notable outliers are *Ulmus uyematsui* (distributed in the mountains of Taiwan, with precipitation between 2000 and 4000 mm per year), as well as *U. pumila*, *U. davidiana,* and *U. macrocarpa*. The last three species possess a broad climatic range, are cold resistant, and occur in monsoon-influenced climates with high seasonality of temperature and precipitation. Our results show that *U. pumila* has a particularly large climatic plasticity among Ulmaceae in terms of temperature and seasonality. It is also one of the most drought-resistant species among the families. These characteristics make this species well adapted for many anthropogenic habitats and probably explain why *U. pumila* is the main species of Ulmaceae reported as invasive [48].

Reliable and precise information about the ecology of the different species of Ulmaceae is scarce, especially for species growing in the tropics or in Asia. Our synthetized compilation (Table 3) demonstrates, however, that a high proportion of Ulmaceae (70%) is linked to humid macro- or microhabitats. This proportion may even be higher, as precise information is missing for many species. Notably, more than 26% of Ulmaceae occur exclusively in tropical humid forests with large amounts of precipitation, higher than 1500 mm per year (e.g., several *Ampelocera* and tropical *Ulmus* species). Moreover, nearly 20% of Ulmaceae are trees exclusive to alluvial and riparian forests or found growing only along river and stream banks. The best examples are North American *Planera aquatica* and *Ulmus americana*, growing exclusively in swamps, along the shores and banks of lakes and rivers and in alluvial flood plains, thus supporting well-waterlogged conditions [49]. In Europe, similar ecological preferences are found for *Ulmus laevis*, which occurs exclusively in riparian forests along large rivers [36]. The two other European *Ulmus* species are also frequently found in alluvial woods, although not exclusively. Furthermore, all six *Zelkova* species are linked to humid habitat conditions to a certain level. East Asiatic *Zelkova* species are particularly common in forests in mountainous regions with high precipitation, as well as in humid ravines and along streams and rivers [1]. This ecological preference is even more pronounced for the Mediterranean members of the genus, with *Z. sicula* growing exclusively along a thalweg, filled in winter with water [47]; and to a lesser extent with *Z. abelicea*, forming large populations only in mountainous areas around winter-moist dolines, summer dry riverbeds or on northern slopes [50].

### 3.3. Threats and Conservation Status

Nearly one-fourth of all the Ulmaceae species are threatened according to the IUCN Red List (Table 3). Two Ulmaceae species are on the brink of extinction: the Anhui elm (*Ulmus gaussenii*) and the Sicilian zelkova (*Zelkova sicula*) [51,52]. *Ulmus gaussenii* is a narrow endemic species growing in Langya Mountain in Anhui Province of Eastern China [53]. Due to low fertility and habitat destruction, the number of individuals has drastically decreased in recent decades. This is probably the rarest Ulmaceae of the world, since only 26 mature and senescing individual trees are known, growing in a single population and covering an area of less than 10 hectares [54]. *Zelkova sicula*, discovered only in 1991, is also a narrow endemic, occurring only in the Iblei Mountains on the Mediterranean island of Sicily in Italy [52]. The species is known from only two populations, 17 km apart, consisting together of ca. 1860 individuals and covering an extremely small total area of only 0.68 hectares [55]. Furthermore, recent molecular investigations revealed that each population is clonal and considered to be issuing centuries-long sprouting of two single surviving genetic individuals [56,57].

More generally, the genus *Zelkova* is the most endangered group of the elm family, since, in addition to *Z. sicula*, all other members of the genus are endangered (IUCN category EN, *Z. abelicea*), vulnerable (VU, *Z. carpinifolia*, *Z. sinica,* and *Z. schneideriana*) or nearly threatened (NT, *Z. serrata*) according to the IUCN Red List [28,58]. Particularly worrying is the situation of *Z. abelicea*, an endemic species of the Mediterranean island of Crete in Greece [1]. In this species, only well-developed trees can produce fruits. However, the overwhelming majority of individuals are dwarfed and nonfruiting due to overbrowsing by goats [50]. Additionally, the majority of fruit are empty, which is probably due to unfavorable climatic conditions such as pronounced and recurrent droughts [5]. The regeneration of populations via seedlings is nearly impossible due to overgrazing, trampling and soil erosion caused by omnipresent large caprine and ovine flocks [59].

In the genus *Ulmus*, two species are endangered (EN, *U. americana* and *U. chenmoui*), and two species are vulnerable (VU, *U. elongata* and *U. wallichiana*). Although American elm (*U. americana*) still forms large populations in the western parts of the USA and Canada, it is the most susceptible North American elm species to introduced and invasive Dutch elm disease (DED), caused by the fungi *Ophiostoma ulmi* and *O. novo-ulmi* [58,60]. The population decline during the next 100 years is supposed to reach up to 80% due to the continuing threat of DED and due to the destruction of its preferred habitat [60]. European elms were also severely impacted by the DED with a severe mortality [38]. Their conservation status is unclear and should be studied in detail. The other three threatened *Ulmus* species occur in Asia. *Ulmus chenmoui* and *U. elongata* are endemic to China, the number of their populations is very restricted, and their original habitat has been largely destroyed [58,61,62]. *Ulmus wallichiana* is widely distributed in the Himalayan region (Afghanistan, Pakistan, India, and Nepal). However, the species is excessively exploited for fodder and fuel wood, and mature reproducing individuals are extremely rare [63].

Among the neotropical Ulmaceae, *Ampelocera albertiae* (IUCN category endangered, EN, [64]) is the most threatened species. This species is a narrow endemic of Colombia, known from only five populations and is heavily affected by cattle ranching, mining activities, and artificial forest plantations.

Several additional Ulmaceae species that have not yet been assessed and thus are not included on the IUCN Red List [58] have a high probability of being globally threatened. This is probably the case for Mexican/Mesoamerican *Ulmus ismaelis* [65]. The taxon is known only from very few and highly isolated populations in Mexico, Salvador, and Honduras [66]. Similarly, numerous Chinese *Ulmus* species possess very restricted distribution areas (e.g., *U. harbinensis*, *U. lamellosa*, *U. mianzhuensis*, *U. prunifolia*, and *U. pseudopropinqua*), and/or their populations are decreasing due to environmental degradation and habitat loss [67].

Ulmaceae is an evolutionarily ancient family possessing high scientific and conservation value. Much more fieldwork, research, and conservation efforts should be undertaken, especially within the tropical clade and for threatened species with restricted distributions and/or weak biological and ecological knowledge.

## 4. Materials and Methods

### 4.1. Taxonomic Division and Species List

The generic division of Ulmaceae was based on recent taxonomic treatments and molecular analyses [10,15,18,19,20,21,23,24,68]. The order of the genera in our study follows the phylogenetic trees of Manchester and Tiffney [68] and Jia et al. [10], dividing the elm family into two clades: (1) a tropical clade with *Ampelocera*, *Phyllostylon,* and *Holoptelea* and (2) a temperate clade with *Hemiptelea*, *Zelkova*, *Planera,* and *Ulmus*.

The species numbers of the poorly studied neotropical genera *Ampelocera* and *Phyllostyllon* were extracted from two detailed monographies by Todzia [6,7], whereas for the third tropical genus, *Holoptelea*, information was obtained from Boratynska [3] and Todzia [9]. Two genera of the temperate clade, *Planera* and *Hemiptelea*, are monotypic [3,9,49,69]. The species number of the genus *Zelkova* was based on taxonomic and biogeographic compilations and recent molecular studies [1,28,57,70]. The most challenging issue is the taxonomic division of the species-rich genus *Ulmus*. In our study, we extracted taxonomic information from eFloras.org: Flora of China, Flora of North America, Flora of Pakistan, Flora Mesoamericana [49,69,71,72], and several other biogeographic treatments and molecular studies [3,18,36,65,73,74]. We worked at the species level and did not distinguish between subspecies or varieties.

### 4.2. Data Collection

A literature review was performed to explore the available data about the distribution, ecology, and conservation status of all extant species. Our review protocol was based on Xiao and Watson [75]. We took advantage of open-access online resources, recently published monographs and articles in diverse fields, where useful information could be found. The grey literature was also occasionally consulted. For online research, we used the species names as keyword, alone or accompanied by words like “distribution”, “map”, “ecology”, “habitat”, etc. References with inaccurate data (e.g., commercial horticultural websites, personal websites) were excluded in the process. We assessed the quality of the data mainly by crossing them. When the information was concordant and apparently not of the same origin, the references were not excluded. When there was no concordance, we only kept the most reliable sources (known institutions, peer reviewed articles, monographs). All kind of data were taken into account and extracted (maps, tables, texts). The full list of references for each species can be found in Supplementary file S1 in Supplementary material. The authors of all Latin names for Ulmaceae species included in this study, are given in Supplementary file S2. The most important resources are cited here [6,7,12,28,33,36,49,58,69,76,77,78,79,80,81,82,83]. The conservation status assessments and information on threats were taken from the IUCN Red List [58].

### 4.3. Species Distribution

Distribution maps for each species were georeferenced and produced on GIS (geographic information system) software [84]. The map background comes from different sources [85,86]. The distribution area of each species was determined using literature data, but to improve the reliability and to obtain the most parsimonious results, we also crossed the data with other information such as altitude or climate. The latter was considered by using the Köppen–Geiger climate classification system [87], which was recently made available at a 1 km resolution [88]. The altitudinal data (30 arc-second resolution) were downloaded from WorldClim version 2.1 [89] and are derived from the Shuttle Radar Topography Mission (SRTM) [90]. Finally, for some species, local experts were asked to examine the distribution maps. Only the natural range was considered, although for some species that have been widely planted, this delimitation was not obvious (Supplementary file S2).

### 4.4. Species Macroclimatic Niche

The distribution of each species was used to assess the realized climatic niche of natural populations. Global data of 19 bioclimatic variables (e.g., mean annual temperature and precipitation, seasonality; the full list of variables is shown in Supplementary file S3) were downloaded from WorldClim at a high resolution (30 arc-seconds) for the 1970–2000 period [91]. For each species, we generated 1000 random points in the area of its distribution, where climatic data were extracted. For each variable, we only kept data between the 5th and 95th percentiles to perform the analyses to remove unwanted outliers due to imprecision in species distributions and for more caution in the evaluation of species climatic preferences. The resolution of the climatic data (30 arc-seconds, ~1 km) does not capture microclimates (e.g., in ravines and slopes), which have been found to be important for some species (e.g., *Zelkova abelicea*, [92]). We therefore must consider that our analyses represent macroclimatic preferences.

The different Köppen–Geiger climates were also recorded for each species, according to its distribution. The Köppen–Geiger climate classification system uses a 2- or 3-letter abbreviation to designate each climate type [87,88]. The first letter indicates the main climate: A—tropical, B—arid, C—temperate, D—cold, and E—polar. The second letter indicates the seasonal precipitation type: m—monsoon, w—savannah, W—desert, S—steppe, s—dry summer, w—dry winter, and f—no dry season. The third letter gives precision regarding the temperature (h—hot, k—cold, a—hot summer, b—warm summer, c—cold summer, and d—very cold winter).

### 4.5. Statistical Analyses

All data analyses and graphs were performed with R [93]. Ordination was performed with the nonmetric multidimensional scaling procedure (NMDS) using the package vegan [94,95,96]. The 19 climatic variables available in WorldClim were included in the analysis (see Supplementary file S3). Precipitation data were square-root transformed, and all climatic data were standardized before performing NMDS, with the Euclidean distance as the distance measure.

## Figures and Tables

**Table 1:** Summary of the taxonomic division, genera and species number, and general distribution of the elm family (Ulmaceae). # Species: number of species. Parenthesis: number of species by region.

Clade	Genus	# Species	General Distribution
Tropical clade	*Ampelocera*	9	South America and Mesoamerica (8), Caribbean (1)
	*Holoptelea*	2	Africa (1) Asia (1)
	*Phyllostylon*	2	South America (1) South America, Mesoamerica and Caribbean (1)
Temperate clade	*Hemiptelea*	1	Eastern Asia
	*Planera*	1	North America
	*Ulmus*	35	North America (6) Mesoamerica (2) Europe and Western Asia (3) Asia (24)
	*Zelkova*	6	Mediterranean Europe and Western Asia (3) Eastern Asia (3)
Total		56	

**Table 2:** Division of Ulmaceae according to their macro- and microhabitats, with a focus on humid habitats. # Species: number of species.

Category	# Species	%
Species of humid macrohabitats (equatorial and tropical humid rainforests, large amount (>1500 mm) of annual precipitation)	15	26.8
Species of moist microhabitats (alluvial and riparian forests, moist ravines), generally exclusively	11	19.6
Species often occurring in moist microhabitats but also in other habitat types	13	23.2
Species occurring in other habitat types or scarce information available	17	30.4

**Table 3:** Conservation status of the Ulmaceae species. IUCN categories: CR—critically endangered, EN—endangered, VU—vulnerable, NT—nearly threatened, LC—least concerned, DD—data deficiency (IUCN 2020). # Species: number of species.

IUCN Category	# Species	Species
CR	2	*Ulmus gaussenii*, *Zelkova sicula*
EN	4	*Ampelocera albertiae*, *Ulmus americana*, *U. chenmoui*, *Zelkova abelicea*
VU	5	*Ulmus elongata*, *U. wallichiana*, *Zelkova carpinifolia*, *Z. sinica*, *Z. schneideriana*
NT	2	*Ampelocera longissima*, *Zelkova serrata*
LC	22	*Ampelocera edentula*, *A. hottlei*, *A. macphersonii*, *A. macrocarpa*, *A. ruizii*, *Hemiptelea davidii*, *Holoptelea grandis*, *Phyllostylon rhamnoides*, *Planera aquatica*, *Ulmus alata*, *U. castaneifolia*, *U. crassifolia*, *U. davidiana*, *U. laciniata*, *U. rubra*, *U. macrocarpa*, *U. parvifolia*, *U. pumila*, *U. serotina*, *U. szechuanica*, *U. thomasii*, *U. mexicana*
DD	3	*Ulmus glabra*, *U. laevis*, *U. minor*
Not assessed	18	*Ampelocera cubensis*, *A. glabra*, *Holoptelea integrifolia*, *Phyllostylon brasiliense*, *Ulmus androssowii*, *U. bergmanniana*, *U. changii*, *U. glaucescens*, *U. harbinensis*, *U. ismaelis*, *U. lamellosa*, *U. lanceifolia*, *U. mianzhuensis*, *U. microcarpa*, *U. prunifolia*, *U. pseudopropinqua*, *U. uyematsui*, *U. villosa*

## Data Availability

Data available from the Zenodo open-access repository: https://doi.org/10.5281/zenodo.4600469, (accessed on 1 April 2021).

## Supplementary Materials

The following are available online at https://www.mdpi.com/article/10.3390/plants10061111/s1, Supplementary file S1: Full list of references for the distribution of each species, Supplementary file S2: Chorological maps of all Ulmaceae species, Supplementary file S3: List of bioclimatic variables.
