# Supplementary material for: Biogeographic Overview of Ulmaceae: Diversity, Distribution, Ecological Preferences, and Conservation Status
Source: Plants (Basel). 2021 May 31;10(6):1111. doi: 10.3390/plants10061111 (PMC8227750; doi:10.3390/plants10061111)
Supplement: Supplementary file 1 [file plants-10-01111-s001.zip › Supplementary file S1 Full list of references for each species.pdf]

Biogeographic Overview of Ulmaceae: Diversity, Distribution, Ecological Preferences, and Conservation Status

Fragnière et al.

Supplementary file S1

Full list of references for each species

In the following table, all the references used to map the distribution of each species are listed.

Species	References
Ampelocera albertiae	[1–5]
Ampelocera cubensis	[1,2,4–6]
Ampelocera edentula	[1–5,7–9]
Ampelocera glabra	[1,2,4,5,7,10,11]
Ampelocera hottlei	[1–5,12–15]
Ampelocera longissima	[1–5,16]
Ampelocera macphersonii	[1–5]
Ampelocera macrocarpa	[1–5,17]
Ampelocera ruizii	[1,2,4,5,18]
Hemiptelea davidii	[2,4,19–23]
Holoptelea grandis	[2,4,5,24–26]
Holoptelea integrifolia	[2,4,5,27–31]
Phyllostylon brasiliense	[2,4,7,11,32–34]
Phyllostylon rhamnoides	[2–6,32,33,35]
Planera aquatica	[2,4,36–40]
Ulmus alata	[2,4,36,41–44]
Ulmus americana	[2,4,36,41,42,44–46]
Ulmus androssowii	[2,4,20,22,47–50]
Ulmus bergmanniana	[2,4,20,22,48]
Ulmus castaneifolia	[2,4,20,22,48,51]
Ulmus changii	[2,4,20,22,48]
Ulmus chenmoui	[2,4,20,22,48,52]
Ulmus crassifolia	[2,4,36,41,42,44,53]
Ulmus davidiana	[2,4,20,22,48,54]
Ulmus elongata	[2,4,20,22,48,55,56]
Ulmus gaussenii	[2,4,20,22,48,57,58]
Ulmus glabra	[2,4,59–67]
Ulmus glaucescens	[2,4,20,22,48,68,69]
Ulmus harbinensis	[2,4,20,22,48]
Ulmus ismaelis	[2,4,70–72]
Ulmus laciniata	[2,4,20,22,48,73,74]
Ulmus lamellosa	[2,4,20,22,48,75,76]
Ulmus laevis	[2,4,59–62,64,66,67,77,78]
Ulmus lanceifolia	[2,4,5,20,22,47,79–81]
Ulmus macrocarpa	[2,4,20,22,48,82]
Ulmus mexicana	[2,4,5,70,83,84]
Ulmus mianzhuensis	[20,85]
Ulmus microcarpa	[2,4,20,22,48,86]
Ulmus minor	[2,4,59,60,62,64,66,67,87,88]
Ulmus parvifolia	[2,4,20,22,48,89–91]
Ulmus prunifolia	[2,4,5,20,22,48]
Ulmus pseudopropinqua	[4,20,22,48]
Ulmus pumila	[2,4,20,22,47,92–97]
Ulmus rubra	[2,4,36,41,42,44,98]
Ulmus serotina	[2,4,36,41,42,99]
Ulmus szechuanica	[2,4,20,22,48,100,101]
Ulmus thomasii	[2,4,36,41,42,44,102]
Ulmus uyematsui	[2,4,5,20,22,48]
Ulmus villosa	[2,4,47,103]
Ulmus wallichiana	[2,4,47,104–106]
Zelkova abelicea	[2,4,107–114]
Zelkova carpinifolia	[2,4,111,115–118]
Zelkova schneideriana	[2,4,20,22,111,119,120]

Zelkova serrata	[2,4,20,22,111,120–122]
Zelkova sicula	[2,4,111,123,124]
Zelkova sinica	[2,4,20,22,111,120,125]

References

1. Todzia, C.A. A Revision of *Ampelocera* (Ulmaceae). *Annals of the Missouri Botanical Garden* **1989**, *76*, 1087–1102, doi:10.2307/2399693.
2. GBIF GBIF: The Global Biodiversity Information Facility Available online: https://www.gbif.org (accessed on 29 September 2020).
3. Gradstein, S.R. *Catálogo de Plantas y Líquenes de Colombia.*; Instituto de Ciencias Naturales, Universidad Nacional de Colombia.; En Bernal, R., S.R. Gradstein & M. Celis (eds.). 2015.: Bogotá, 2020;
4. Hassler, M. *World Plants: Synonymic Checklists of the Vascular Plants of the World (Version Nov 2018). In: Species 2000 & ITIS Catalogue of Life, 2020-09-01 Beta (Roskov Y.; Ower G.; Orrell T.; Nicolson D.; Bailly N.; Kirk P.M.; Bourgoin T.; DeWalt R.E.; Decock W.; Nieukerken E. van; Penev L.; Eds.).*; Species 2000: Naturalis.; Leiden, the Netherlands, 2020;
5. Royal Botanic Gardens Kew Plants of the World Online Available online: http://www.plantsoftheworldonline.org (accessed on 29 September 2020).
6. Acevedo-Rodríguez, P.; Strong, M.T. Catalogue of Seed Plants of the West Indies. **2012**.
7. Carauta, J.P.P. Índice Das Espécies de Ulmaceae Do Brasil. *Rodriguésia* **1974**, *27*, 99–134.
8. Boggan, J.; Funk, V.; Kelloff, C.; Hoff, M.; Cremers, G.; Feuillet, C. *Checklist of the Plants of the Guianas (Guyana, Surinam, French Guiana)*; National Museum of Natural History.; Washington, DC, 1997;
9. Funk, V.; Hollowell, T.; Berry, P.; Kelloff, C.; Alexander, S.N. *Checklist of the Plants of the Guiana Shield (VENEZUELA: Amazonas, Bolivar, Delta Amacuro; GUYANA, SURINAM, FRENCH GUIANA)*; National Museum of Natural History.; Washington, DC, 2007;
10. Pederneiras, L.C.; Costa, A.F. da; Araujo, D.S.D. de; Carauta, J.P.P.; Pederneiras, L.C.; Costa, A.F. da; Araujo, D.S.D. de; Carauta, J.P.P. Ulmaceae, Cannabaceae e Urticaceae das restingas do estado do Rio de Janeiro. *Rodriguésia* **2011**, *62*, 299–313, doi:10.1590/2175-7860201162207.
11. Pederneiras, L.C.; Machado, A.F.P.; Pederneiras, L.C.; Machado, A.F.P. Flora do estado do Rio de Janeiro: Ulmaceae. *Rodriguésia* **2017**, *68*, 541–543, doi:10.1590/2175-7860201768216.
12. Nevling, L.I. Flora of Panama. Part IV. Fascicle II. *Annals of the Missouri Botanical Garden* **1960**, *47*, 81–203, doi:10.2307/2394704.
13. Cowan, C.P. Listados Floristicos de Mexico, I. Flora de Tabasco Available online: http://www.ibiologia.unam.mx/BIBLIO68/fulltext/lfl1.html (accessed on 29 September 2020).
14. Nee, M.; Gama, L.C. *Flora de Veracruz: Ulmaceae*; Instituto de Investigaciones sobre Recursos Bioticos, 1984;
15. Tropicos.org. Missouri Botanical Garden Tropicos | Name - Ampelocera Hottlei (Standl.) Standl. Available online: http://legacy.tropicos.org/Name/33300107?projectid=7 (accessed on 15 November 2020).
16. León-Yánez, S.& P. IUCN Red List of Threatened Species: Ampelocera Longissima Available online: https://www.iucnredlist.org/en (accessed on 29 September 2020).
17. Condit, R. IUCN Red List of Threatened Species: Ampelocera Macrocarpa Available online: https://www.iucnredlist.org/en (accessed on 29 September 2020).
18. Botanic Gardens Conservation International (BGCI); IUCN SSC Global Tree Specialist Group IUCN Red List of Threatened Species: Ampelocera Ruizii Available online: https://www.iucnredlist.org/en (accessed on 29 September 2020).

19. Minaki, M.; Noshiro, S.; Suzuki, M. Hemiptelea Mikii Sp. Nov. (Ulmaceae), Fossil Fruits and Woods from the Pleistocene of Central Japan. *Bot. Mag. Tokyo* **1988**, *101*, 337–351, doi:10.1007/BF02488081.
20. Wu, Z.; Raven, P.H.; Hong, D. Flora of China. Volume 5: Ulmaceae through Basellaceae. *Flora of China. Volume 5: Ulmaceae through Basellaceae.* **2003**.
21. Yun-peng, B.; Da-yong, H.; Yan-hong, D.; Yu-jing, Z.; Jian-dong, L. Structural Characteristics of Hemiptelea Davidii Community on Kerqin Sandy Land. *Chinese Journal of Applied Ecology* **2008**, *19*, 257.
22. Fang, J.; Wang, Z.; Tang, Z. *Atlas of Woody Plants in China: Distribution and Climate*; Springer Science & Business Media, 2011; Vol. 1; ISBN 3-642-15017-9.
23. Lin, Q.-W. IUCN Red List of Threatened Species: Hemiptelea Davidii Available online: https://www.iucnredlist.org/en (accessed on 3 October 2020).
24. PROTA Holoptelea Grandis (Hutch.) Mildbr. Available online: https://www.prota4u.org/database/protav8.asp?fr=1&g=pe&p=Holoptelea+grandis+(Hutch.)+Mildbr. (accessed on 18 November 2020).
25. Conservatoire et Jardin botaniques de la Ville de Genève CJB - African Plant Database - Holoptelea Grandis Available online: http://www.ville-ge.ch/musinfo/bd/cjb/africa/details.php?langue=an&id=95089 (accessed on 16 August 2020).
26. Barstow, M. IUCN Red List of Threatened Species: Holoptelea Grandis Available online: https://www.iucnredlist.org/en (accessed on 16 August 2020).
27. Ganie, S.A.; Yadav, S.S. Holoptelea Integrifolia (Roxb.) Planch: A Review of Its Ethnobotany, Pharmacology, and Phytochemistry. *Biomed Res Int* **2014**, *2014*, 401213–401213, doi:10.1155/2014/401213.
28. Fern, K. Tropical Plants Database, Holoptelea Integrifolia - Useful Tropical Plants Available online: http://tropical.theferns.info/viewtropical.php?id=Holoptelea+integrifolia (accessed on 17 October 2020).
29. Sankara Rao, K.; Raja, K.S.; Deepak, K.; Arun Singh, R.K.; Gopalakrishna, B. Flora of Peninsular India Available online: http://peninsula.ces.iisc.ac.in/plants.php?name=Holoptelea integrifolia. (accessed on 19 November 2020).
30. efloras.org Holoptelea Integrifolia in Annotated Checklist of the Flowering Plants of Nepal Available online: http://www.efloras.org/florataxon.aspx?flora_id=110&taxon_id=220006469 (accessed on 17 October 2020).
31. India Biodiversity Portal Holoptelea Integrifolia Planch. Available online: https://indiabiodiversity.org/species/show/31452 (accessed on 17 October 2020).
32. Taubert, P. Die Gattung Phyllostylon Capan. Und Ihre Beziehungen Zu Samaroceltis Poiss. *Österreichische Botanische Zeitschrift* **1890**, 406–410.
33. Todzia, C.A. A Reevaluation of the Genus *Phyllostylon* (Ulmaceae). *SIDA, Contributions to Botany* **1992**, *15*, 263–270.
34. Prado, D. IUCN Red List of Threatened Species: Phyllostylon Orthopterum Available online: https://www.iucnredlist.org/en (accessed on 29 September 2020).
35. Dottori, N.; Hunziker, A.T. Flora Fanerogámica Argentina, Ulmaceae 2019.
36. Sherman-Broyles, S.L.; Barker, W.T.; Schulz, L.M. Ulmaceae. *Flora of North America* **1997**, *3*, 368–380.
37. Wunderlin, R.P.; Hansen, B.F.; Franck, A.R.; Essig, F.B. Atlas of Florida Vascular Plants. *Atlas of Florida vascular plants.* **2016**.
38. Botanic Gardens Conservation International (BGCI) IUCN Red List of Threatened Species: Planera Aquatica Available online: https://www.iucnredlist.org/en (accessed on 6 October 2020).
39. United States Department of Agriculture - Forest Service Water Elm (Planera Aquatica) - Climate Change Atlas Available online: https://www.fs.fed.us/nrs/atlas/tree/722 (accessed on 6 October 2020).

40. USDA PLANTS database Plants Profile for Planera Aquatica (Planertree) Available online: https://plants.usda.gov/core/profile?symbol=PLAQ (accessed on 6 October 2020).
41. Little, J.; Elbert, L. Atlas of United States Trees, Vol. 1. *Conifers and important hardwoods. USDA Forest Service Miscellaneous Publication* **1971**, *1146*.
42. Elbert, L.; Little, J. *Checklist of United States Trees (Native and Naturalized)*; DC: Forest Service, US Department of Agriculture, 1979;
43. Stritch, L. IUCN Red List of Threatened Species: Ulmus Alata Available online: https://www.iucnredlist.org/en (accessed on 20 October 2020).
44. United States Department of Agriculture - Forest Service Northern Research Station, Climate Change Atlas Available online: https://www.nrs.fs.fed.us/ (accessed on 20 October 2020).
45. Bey, C.F. Ulmus Americana L. American Elm. *Silvics of North America* **1990**, *2*, 801–807.
46. Stritch, L.; Rivers, M.C.; Barstow, M. IUCN Red List of Threatened Species: *Ulmus Americana* Available online: https://www.iucnredlist.org/en (accessed on 20 October 2020).
47. Melville, R.; Heybroek, H.M. The Elms of the Himalaya. *Kew Bulletin* **1971**, 5–28.
48. Fu, L.; Xin, Y. Elms of China. In *The Elms*; Springer, 2000; pp. 21–44.
49. Akhter, R. Ulmus Chumlia in Flora of Pakistan @ Efloras.Org Available online: http://www.efloras.org/florataxon.aspx?flora_id=5&taxon_id=242353343 (accessed on 5 November 2020).
50. efloras.org Ulmus Chumlia in Annotated Checklist of the Flowering Plants of Nepal @ Efloras.Org Available online: http://www.efloras.org/florataxon.aspx?flora_id=110&taxon_id=242353343 (accessed on 5 November 2020).
51. Lin, Q.-W.; Botanic Gardens Conservation International (BGCI) IUCN Red List of Threatened Species: Ulmus Castaneifolia Available online: https://www.iucnredlist.org/en (accessed on 5 November 2020).
52. World Conservation Monitoring Centre IUCN Red List of Threatened Species: Ulmus Chenmoui Available online: https://www.iucnredlist.org/en (accessed on 5 November 2020).
53. Barstow, M. IUCN Red List of Threatened Species: Ulmus Crassifolia Available online: https://www.iucnredlist.org/en (accessed on 20 October 2020).
54. Botanic Gardens Conservation International (BGCI) IUCN Red List of Threatened Species: Ulmus Davidiana Available online: https://www.iucnredlist.org/en (accessed on 5 November 2020).
55. World Conservation Monitoring Centre IUCN Red List of Threatened Species: Ulmus Elongata Available online: https://www.iucnredlist.org/en (accessed on 4 November 2020).
56. Gao, J.-G.; Wu, Y.-H.; Xu, G.-D.; Li, W.-Q.; Yao, G.-H.; Ma, J.; Liu, P. Phylogeography of *Ulmus Elongata* Based on Fourier Transform-Infrared Spectroscopy (FTIR), Thermal Gravimetric and Differential Thermal Analyses. *Biochemical Systematics and Ecology* **2012**, *40*, 184–191.
57. World Conservation Monitoring Centre IUCN Red List of Threatened Species: *Ulmus Gaussenii* Available online: https://www.iucnredlist.org/en (accessed on 4 November 2020).
58. Zhang, Q.; Zhang, H.; Li, Q.; Bai, R.; Ning, E.; Cai, X. Characterization of the Complete Chloroplast Genome Sequence of an Endangered Elm Species, *Ulmus Gaussenii* (Ulmaceae). *Conservation Genetics Resources* **2019**, *11*, 71–74.
59. Hultén, E.; Fries, M. *Atlas of North European Vascular Plants North of the Tropic of Cancer*; Koeltz Scientific, 1986; ISBN 3-87429-263-0.
60. Naturhistoriska Riksmuseet Den Virtuella Floran: Ulmus L. - Almar Available online: http://linnaeus.nrm.se/flora/di/ulma/ulmus/welcome.html (accessed on 13 September 2020).
61. Afonin, A.N.; Greene, S.L.; Dzyubenko, N.I.; Frolov, A.N.; Afanasenko, O.S.; Berim, M.N.; Bilder, I.V.; Budrevskaya, I.A.; Vershinin, A.P.; Gagkaeva, T.Y. Interactive Agricultural Ecological Atlas of Russia and Neighboring Countries. Economic Plants and Their Diseases, Pests and Weeds Available online: http://www.agroatlas.ru (accessed on 15 October 2020).
62. Caudullo, G.; De Rigo, D. *Ulmus*-Elms in Europe: Distribution, Habitat, Usage and Threats. *European atlas of forest tree species* **2016**, 186–188.

63. Barstow, M.; Rivers, M.C. IUCN Red List of Threatened Species: Ulmus Glabra Available online: https://www.iucnredlist.org/en (accessed on 15 October 2020).
64. Caudullo, G.; Welk, E.; San-Miguel-Ayanz, J. Chorological Maps for the Main European Woody Species. *Data Brief* **2017**, *12*, 662–666, doi:10.1016/j.dib.2017.05.007.
65. Thomas, P.A.; Stone, D.; Porta, N.L. Biological Flora of the British Isles: Ulmus Glabra. *Journal of Ecology* **2018**, *106*, 1724–1766, doi:https://doi.org/10.1111/1365-2745.12994.
66. Babac, M.T.; Uslu, E.; Bakis, Y. TÜBİVES - Turkish Plants Data Service Available online: www.tubives.com (accessed on 15 October 2020).
67. Botanical Society of Britain & Ireland BSBI Big Database Available online: bsbidb.org.uk (accessed on 15 October 2020).
68. Ware, G.H. Little-Known Asian Elms: Urban Tree Possibilities. *Journal of Arboriculture* **1980**, *6*, 197–199.
69. Jiang, Y.; Kang, M.; Zhu, Y.; Xu, G. Plant Biodiversity Patterns on Helan Mountain, China. *Acta Oecologica* **2007**, *32*, 125–133.
70. Todzia, C.A.; Panero, J.L. A New Species of *Ulmus* (Ulmaceae) from Southern Mexico and a Synopsis of the Species in Mexico. *Brittonia* **1998**, *50*, 343–347.
71. Linares, J.L. Primer Registro de *Ulmus Ismaelis* (Ulmaceae) Para Centroamérica. *Revista mexicana de biodiversidad* **2005**, *76*, 95–96.
72. López-Cruz, A.; Gómez-Domínguez, H.; Pérez-Farrera, M.Á.; Espinoza-Jiménez, J.A. Ulmus Ismaelis (Ulmaceae) y Pilocarpus Racemosus Var. Racemosus (Rutaceae), Nuevos Registros Para La Flora de Chiapas, México. *Revista mexicana de biodiversidad* **2013**, *84*, 985–988.
73. Chang, C.S.; Kim, S. The Woody Plants of Korea Available online: http://florakorea.myspecies.info/en/file/1240 (accessed on 30 October 2020).
74. Lin, Q.-W.; Botanic Gardens Conservation International (BGCI) IUCN Red List of Threatened Species: Ulmus Laciniata Available online: https://www.iucnredlist.org/en (accessed on 30 October 2020).
75. Liu, L.; Chen, W.; Zheng, X.; Li, J.; Yan, D.-T.; Liu, L.; Liu, X.; Wang, Y.-L. Genetic Diversity of Ulmus Lamellosa by Morphological Traits and Sequence-Related Amplified Polymorphism (SRAP) Markers. *Biochemical Systematics and Ecology* **2016**, *66*, 272–280.
76. Yan, D.; Chen, W.; Liu, L.; Li, J.; Liu, L.; Wang, Y. Change in Current and Future Geographic Distributions of Ulmus Lamellosa in China. *Journal of Forestry Research* **2018**, *29*, 1147–1156.
77. Koskela, J. European Information System on Forest Genetic Resources (EUFGIS). *URL: http://portal. eufgis. org/maps. html, Stand* **2012**, *20*, 2007.
78. Harvey-Brown, Y. IUCN Red List of Threatened Species: Ulmus Laevis Available online: https://www.iucnredlist.org/en (accessed on 15 October 2020).
79. Maloney, B.K. That Elm Again! *BLUMEA* **1998**, *43*, 121–127.
80. Newman, M.; Ketphanh, S.; Svengsuksa, B.; Thomas, P.; Sengdala, K.; Lamxay, V.; Armstrong, K. *A Checklist of the Vascular Plants of Lao PDR.*; Royal Botanic Garden Edinburgh, 2007; ISBN 1-906129-04-5.
81. Flora Malesiana Ulmus Lanceaefolia | Flora Malesiana Available online: http://portal.cybertaxonomy.org/flora-malesiana/node/4450#distribution (accessed on 30 October 2020).
82. Botanic Gardens Conservation International (BGCI) IUCN Red List of Threatened Species: Ulmus Macrocarpa Available online: https://www.iucnredlist.org/en (accessed on 30 October 2020).
83. Moreira-González, I.; Arnáez-Serrano, E. Ulmus Mexicana (Liebm.) Planch., Tropical Tree Seed Manual. *RNGR, USDA Forest Service, USA* **2003**, 770–771.
84. Nugent, J. INaturalist: Citizen Science for 21st-Century Naturalists. *Science Scope* **2018**, *41*, 12.
85. Tongpei, Y.; Lin, Y. Ulmus Mianzhuensis, a New Species of Ulmaceae from Sichuan, China. *Zhi wu yan jiu (2001)* **2006**, *26*, 641–643.
86. Mackenthun, G. Handbuch Der Ulmengewächse Available online: https://www.ulmen-handbuch.de/handbuch/home.html (accessed on 27 October 2020).

87. Tela Botanica Smart'Flore Available online: https://www.tela-botanica.org/eflore/consultation/index_mobile.php?module=mobile&referentiel=bdtfx&num_nom=70296 (accessed on 22 November 2020).
88. Barstow, M.; Rivers, M.C.; Harvey-Brown, Y. IUCN Red List of Threatened Species: Ulmus Minor Available online: https://www.iucnredlist.org/en (accessed on 15 October 2020).
89. Akimoto, S. Phylogeny, Evolution and Biogeography of Gall-Forming Aphids (Insecta: Homoptera): A Case Study from the Eriosomatini. In Proceedings of the Neo-Science of Natural History: Integration of Geoscience and Biodiversity Studies: Proceedings of International Symposium on" Dawn of a New Natural History-Integration of Geoscience and Biodiversity Studies" March 5-6, 2004, Sapporo; Graduate School of Science, Hokkaido University, 2004; pp. 19–26.
90. Lin, Q.-W.; Botanic Gardens Conservation International (BGCI) IUCN Red List of Threatened Species: Ulmus Parvifolia Available online: https://www.iucnredlist.org/en (accessed on 27 October 2020).
91. United States Department of Agriculture - Natural Resources Conservation Service Plants Profile for Ulmus Parvifolia (Chinese Elm) Available online: https://plants.usda.gov/core/profile?symbol=ULPA (accessed on 27 November 2020).
92. Hilbig, W.; Knapp, H.D. Vegetation Mosaic and Floristic Elements on the Zonal Forest-Steppe-Border in the Chentej Mountains (Mongolia). *Flora* **1983**, *174*, 1–89.
93. Geng, M.C. A Provenance Test of White Elm (Ulmus Pumila L.) in China. *Silvae Genetica* **1989**, *38*, 37.
94. Ghelardini, L. *Bud Burst Phenology, Dormancy Release and Susceptibility to Dutch Elm Disease in Elms (Ulmus Spp.)*; 2007; Vol. 2007; ISBN 91-85913-33-2.
95. Wesche, K.; Walther, D.; Von Wehrden, H.; Hensen, I. Trees in the Desert: Reproduction and Genetic Structure of Fragmented Ulmus Pumila Forests in Mongolian Drylands. *Flora-Morphology, Distribution, Functional Ecology of Plants* **2011**, *206*, 91–99.
96. Chen, X.; Xu, L. Phenological Responses of Ulmus Pumila (Siberian Elm) to Climate Change in the Temperate Zone of China. *International Journal of Biometeorology* **2012**, *56*, 695–706.
97. Barstow, M. IUCN Red List of Threatened Species: Ulmus Pumila Available online: https://www.iucnredlist.org/en (accessed on 27 October 2020).
98. Stritch, L. IUCN Red List of Threatened Species: Ulmus Rubra Available online: https://www.iucnredlist.org/en (accessed on 20 October 2020).
99. Botanic Gardens Conservation International (BGCI); IUCN SSC Global Tree Specialist Group IUCN Red List of Threatened Species: Ulmus Serotina Available online: https://www.iucnredlist.org/en (accessed on 20 October 2020).
100. Lin, Q.-W.; Botanic Gardens Conservation International (BGCI) IUCN Red List of Threatened Species: Ulmus Szechuanica Available online: https://www.iucnredlist.org/en (accessed on 27 October 2020).
101. Shufang, Y.; Yichao, L.; Shuxiang, F.; Xiaoxu, H.; Yinran, H. The Complete Chloroplast Genome Sequence of Ulmus Szechuanica (Ulmaceae) and Its Phylogenetic Analysis. *Mitochondrial DNA Part B* **2020**, *5*, 2186–2187, doi:10.1080/23802359.2020.1768958.
102. Stritch, L. IUCN Red List of Threatened Species: Ulmus Thomasii Available online: https://www.iucnredlist.org/en (accessed on 20 October 2020).
103. Akhter, R. Ulmus Villosa in Flora of Pakistan @ Efloras.Org Available online: http://www.efloras.org/florataxon.aspx?flora_id=5&taxon_id=250081108 (accessed on 20 October 2020).
104. Akhter, R. Ulmus Wallichiana in Flora of Pakistan @ Efloras.Org Available online: http://www.efloras.org/florataxon.aspx?flora_id=5&taxon_id=242426077 (accessed on 20 October 2020).
105. World Conservation Monitoring Centre IUCN Red List of Threatened Species: Ulmus Wallichiana Available online: https://www.iucnredlist.org/en (accessed on 20 October 2020).

106. Mughal, A.H.; Mugloo, J.A. Elm (*Ulmus Wallichiana*): A Vulnerable Lesser Known Multipurpose Tree Species of Kashmir Valley. *SKUAST Journal of Research* **2016**, *18*, 73–79.
107. Egli, B. A Projeet for the Preservation of Zelkova Abelicea (Ulmaceae), a Threatened Endemie Tree Species from the Mountains of Crete. *Bocconea* **1997**, 506–510.
108. Søndergaard, P.; Egli, B.R. Zelkova Abelicea (Ulmaceae) in Crete: Floristics, Ecology, Propagation and Threats. *will* **2006**, *36*, 317–322, doi:10.3372/wi.36.36126.
109. Kozlowski, G.; Frey, D.; Fazan, L.; Egli, B.; Pirintsos, S. IUCN Red List of Threatened Species: Zelkova Abelicea Available online: https://www.iucnredlist.org/en (accessed on 6 October 2020).
110. Kozlowski, G.; Frey, D.; Fazan, L.; Egli, B.; Bétrisey, S.; Gratzfeld, J.; Garfì, G.; Pirintsos, S. The Tertiary Relict Tree *Zelkova Abelicea* (Ulmaceae): Distribution, Population Structure and Conservation Status on Crete. *Oryx* **2014**, *48*, 80–87.
111. Kozlowski, G.; Gratzfeld, J. Zelkova—*An Ancient Tree. Global Status and Conservation Action.*; Natural History Museum Fribourg: Switzerland, 2013;
112. Bosque, M.; Adamogianni, M.-I.; Bariotakis, M.; Fazan, L.; Stoffel, M.; Garfî, G.; Gratzfeld, J.; Kozlowski, G.; Pirintsos, S. Fine-Scale Spatial Patterns of the Tertiary Relict *Zelkova Abelicea* (Ulmaceae) Indicate Possible Processes Contributing to Its Persistence to Climate Changes. *Reg Environ Change* **2014**, *14*, 835–849, doi:10.1007/s10113-013-0544-1.
113. Fazan, L.; Guillet, S.; Corona, C.; Kozlowski, G.; Stoffel, M. Imprisoned in the Cretan Mountains: How Relict *Zelkova Abelicea* (Ulmaceae) Trees Cope with Mediterranean Climate. *Science of The Total Environment* **2017**, *599–600*, 797–805, doi:10.1016/j.scitotenv.2017.04.047.
114. Goedecke, F.; Bergmeier, E. Ecology and Potential Distribution of the Cretan Endemic Tree Species Zelkova Abelicea. *J Mediterr Ecol* **2018**, *16*, 15–26.
115. Ansin, R.; Gercek, Z. A New Zelkova Taxon for the Flora of Turkey: Zelkova Carpinifolia (Pall.) C. Koch Subsp. Yomraensis Anșin & Gercek, Subsp. Nova. *Turkish Journal of Agriculture and Forestry* **1991**, *15*, 564–575.
116. Kvavadze, E.V.; Connor, S.E. Zelkova Carpinifolia (Pallas) K. Koch in Holocene Sediments of Georgia—an Indicator of Climatic Optima. *Review of Palaeobotany and Palynology* **2005**, *133*, 69–89.
117. Maharramova, E.H.; Safarov, H.M.; Kozlowski, G.; Borsch, T.; Muller, L.A. Analysis of Nuclear Microsatellites Reveals Limited Differentiation between Colchic and Hyrcanian Populations of the Wind-Pollinated Relict Tree Zelkova Carpinifolia (Ulmaceae). *American Journal of Botany* **2015**, *102*, 119–128, doi:10.3732/ajb.1400370.
118. Bétrisey, S.; Yousefzadeh, H.; Kozlowski, G. IUCN Red List of Threatened Species: Zelkova Carpinifolia Available online: https://www.iucnredlist.org/en (accessed on 6 October 2020).
119. Song, Y.-G.; Bétrisey, S.; Kozlowski, G. The IUCN Red List of Threatened Species: Zelkova Schneideriana Available online: https://www.iucnredlist.org/en (accessed on 6 October 2020).
120. Naciri, Y.; Christe, C.; Bétrisey, S.; Song, Y.-G.; Deng, M.; Garfì, G.; Kozlowski, G. Species Delimitation in the East Asian Species of the Relict Tree Genus *Zelkova* (Ulmaceae): A Complex History of Diversification and Admixture among Species. *Molecular Phylogenetics and Evolution* **2019**, *134*, 172–185, doi:10.1016/j.ympev.2019.02.010.
121. Fukatsu, E.; Watanabe, A.; Nakada, R.; Isoda, K.; Hirao, T.; Ubukata, M.; Koyama, Y.; Kodani, J.; Saito, M.; Miyamoto, N.; et al. Phylogeographical Structure in Zelkovaserrata in Japan and Phylogeny in the Genus Zelkova Using the Polymorphisms of Chloroplast DNA. *Conserv Genet* **2012**, *13*, 1109–1118, doi:10.1007/s10592-012-0358-6.
122. Bétrisey, S.; Song, Y.-G.; Kozlowski, G. IUCN Red List of Threatened Species: Zelkova Serrata Available online: https://www.iucnredlist.org/en (accessed on 6 October 2020).
123. Garfì, G.; Carimi, F.; Pasta, S.; Rühl, J.; Trigila, S. Additional Insights on the Ecology of the Relic Tree *Zelkova Sicula* Di Pasquale, Garfì et Quézel (Ulmaceae) after the Finding of a New Population. *Flora - Morphology, Distribution, Functional Ecology of Plants* **2011**, *206*, 407–417, doi:10.1016/j.flora.2010.11.004.

124. Garfì, G.; Pasta, S.; Fazan, L.; Kozlowski, G. IUCN Red List of Threatened Species: *Zelkova Sicula* Available online: https://www.iucnredlist.org/en (accessed on 6 October 2020).
125. Bétrisey, S.; Song, Y.-G.; Liu, Z.; Kozlowski, G. IUCN Red List of Threatened Species: Zelkova Sinica Available online: https://www.iucnredlist.org/en (accessed on 6 October 2020).
