# Supplementary material for: Biogeographic Overview of Ulmaceae: Diversity, Distribution, Ecological Preferences, and Conservation Status
Source: Plants (Basel). 2021 May 31;10(6):1111. doi: 10.3390/plants10061111 (PMC8227750; doi:10.3390/plants10061111)
Supplement: Supplementary file 1 [file plants-10-01111-s001.zip › Supplementary file S2 Distribution maps of all Ulmaceae species.pdf]

Biogeographic Overview of Ulmaceae: Diversity, Distribution, Ecological Preferences, and Conservation Status

Fragnière et al.

Supplementary file S2

Distribution maps of all Ulmaceae species

All species presented in alphabetical order

Ampelocera albertiae

ULMACEAE

Todzia

Ann. Missouri Bot. Gard. 76: 1089, fig. 1 (1989)

IUCN Red list status : ne
*
habitat-ecology : wet tropical mountains
*
climate - Köppen classification : Cfb, Af
*
indicative altitudinal range : 1750 - 1900 m
*
min. latitude : 4.3, max. latitude : 5,
min. longitude : -76.3, max. longitude : -75.5

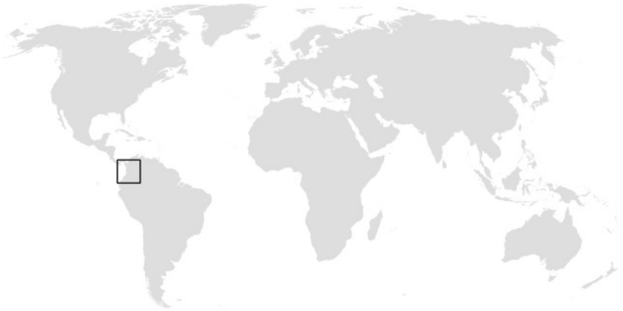

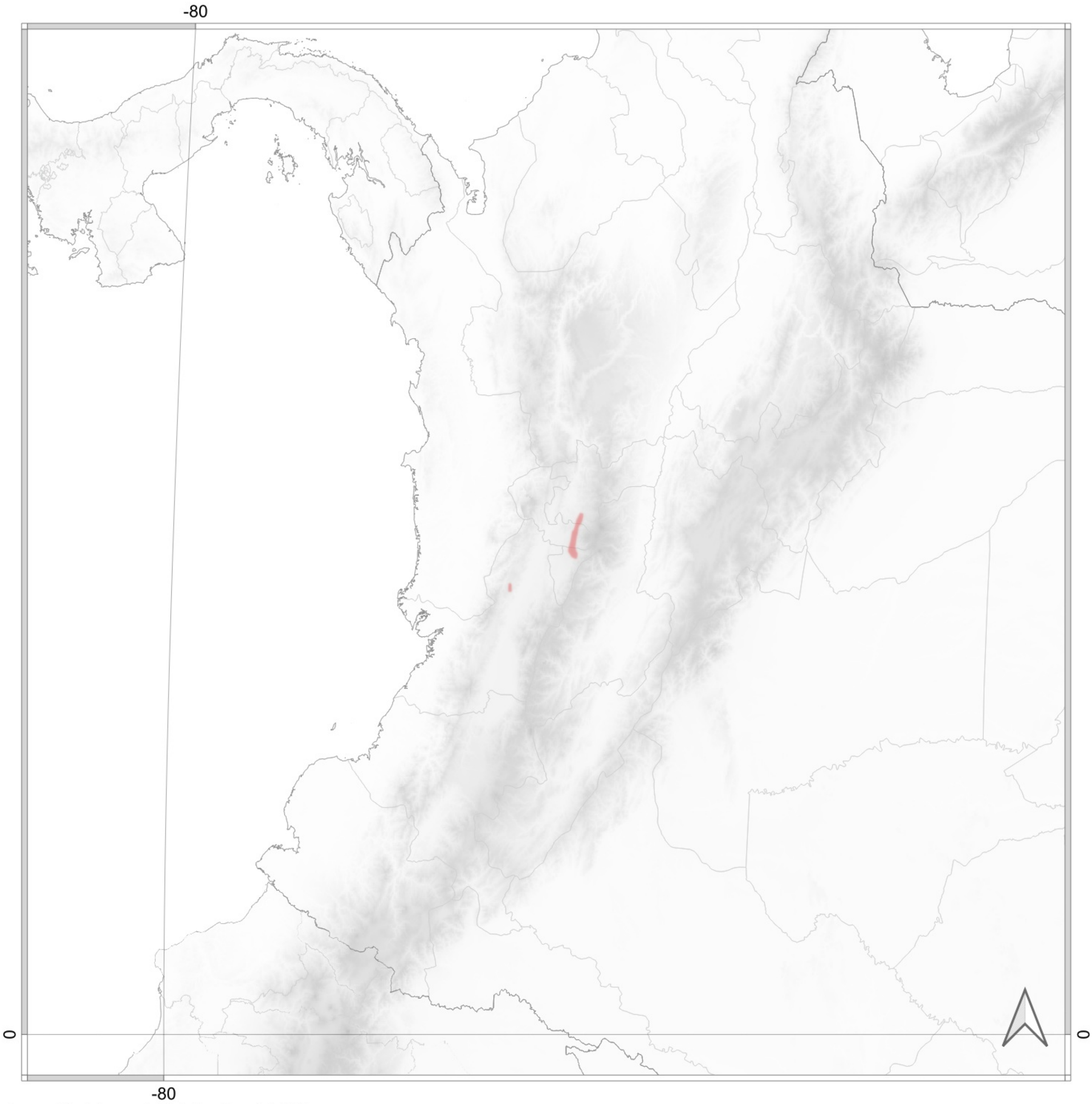

Source of the data : see details in Fragnière et al., 2021

Ampelocera cubensis

ULMACEAE

Griseb.

Cat. Pl. Cub. [Grisebach] 57 (1866)

IUCN Red list status : ne
*
habitat-ecology : costal thickets, limestone cliffs, rocky woods
*
climate - Köppen classification : Aw
*
indicative altitudinal range : 0 - 300 m
*
min. latitude : 17.5, max. latitude : 23.3,
min. longitude : -84.9, max. longitude : -70.9

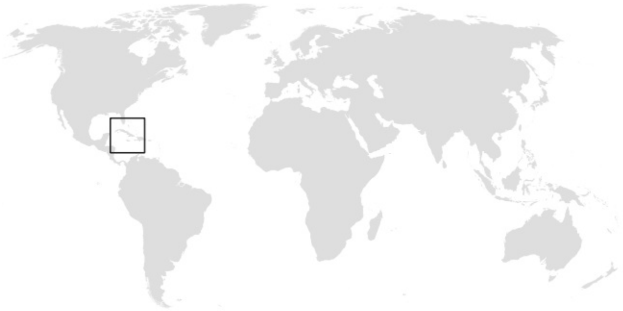

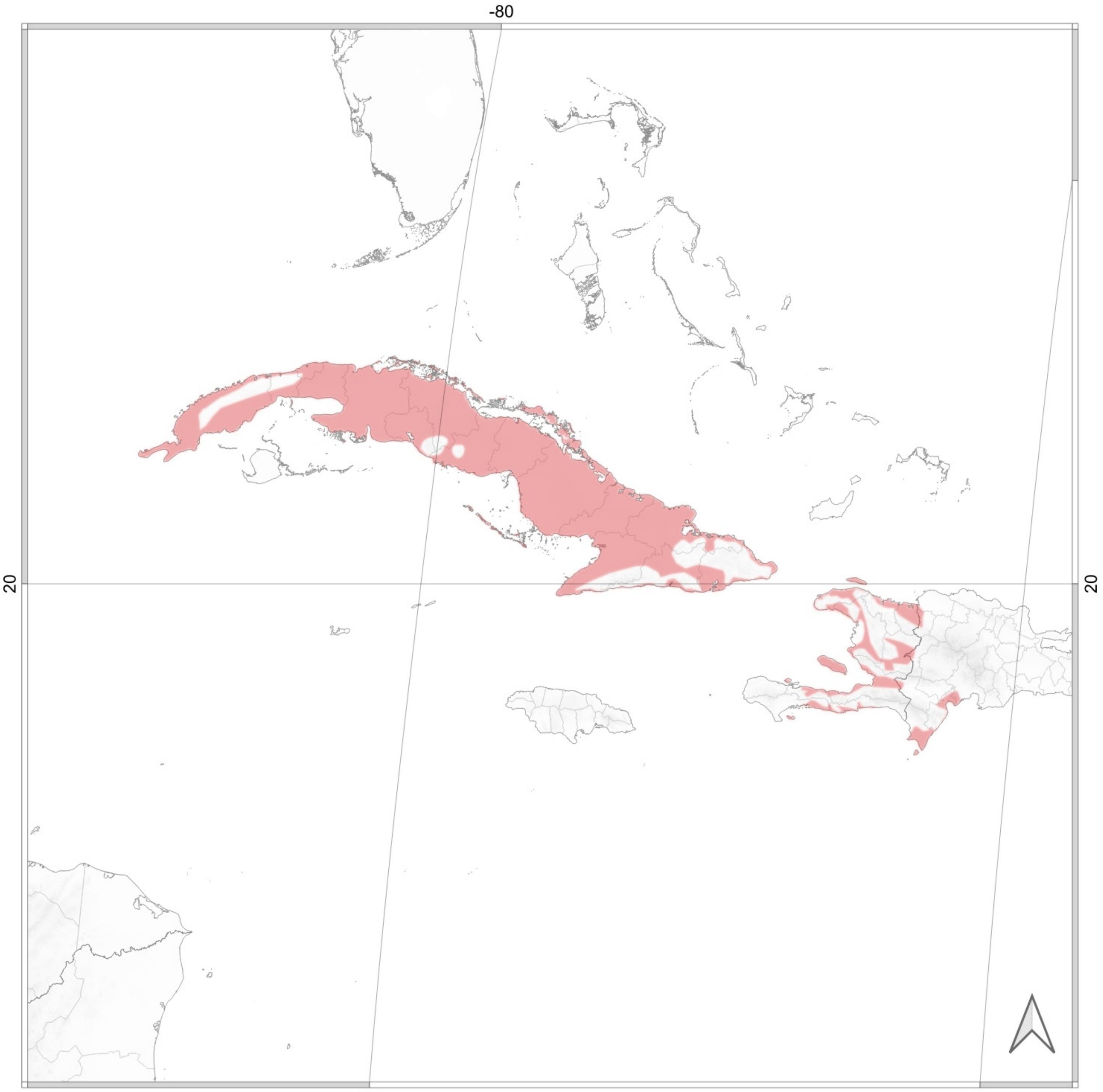

Source of the data : see details in Fragnière et al., 2021

Ampelocera edentula

ULMACEAE

Kuhlm.

Anais Reunião Sul-Amer. Bot. 3: 75, tab. 1 (1940)

IUCN Red list status : ne
*
habitat-ecology : primary tropical moist forests, nonflooded and flooded riverine forests
*
climate - Köppen classification : Af, Am, (Aw)
*
indicative altitudinal range : 0 - 750 m
*
min. latitude : -16.5, max. latitude : 9.8,
min. longitude : -80.1, max. longitude : -46.4

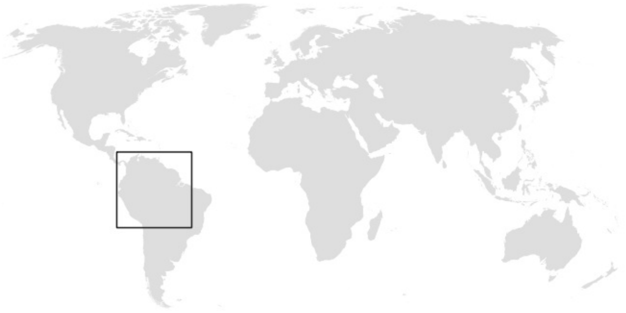

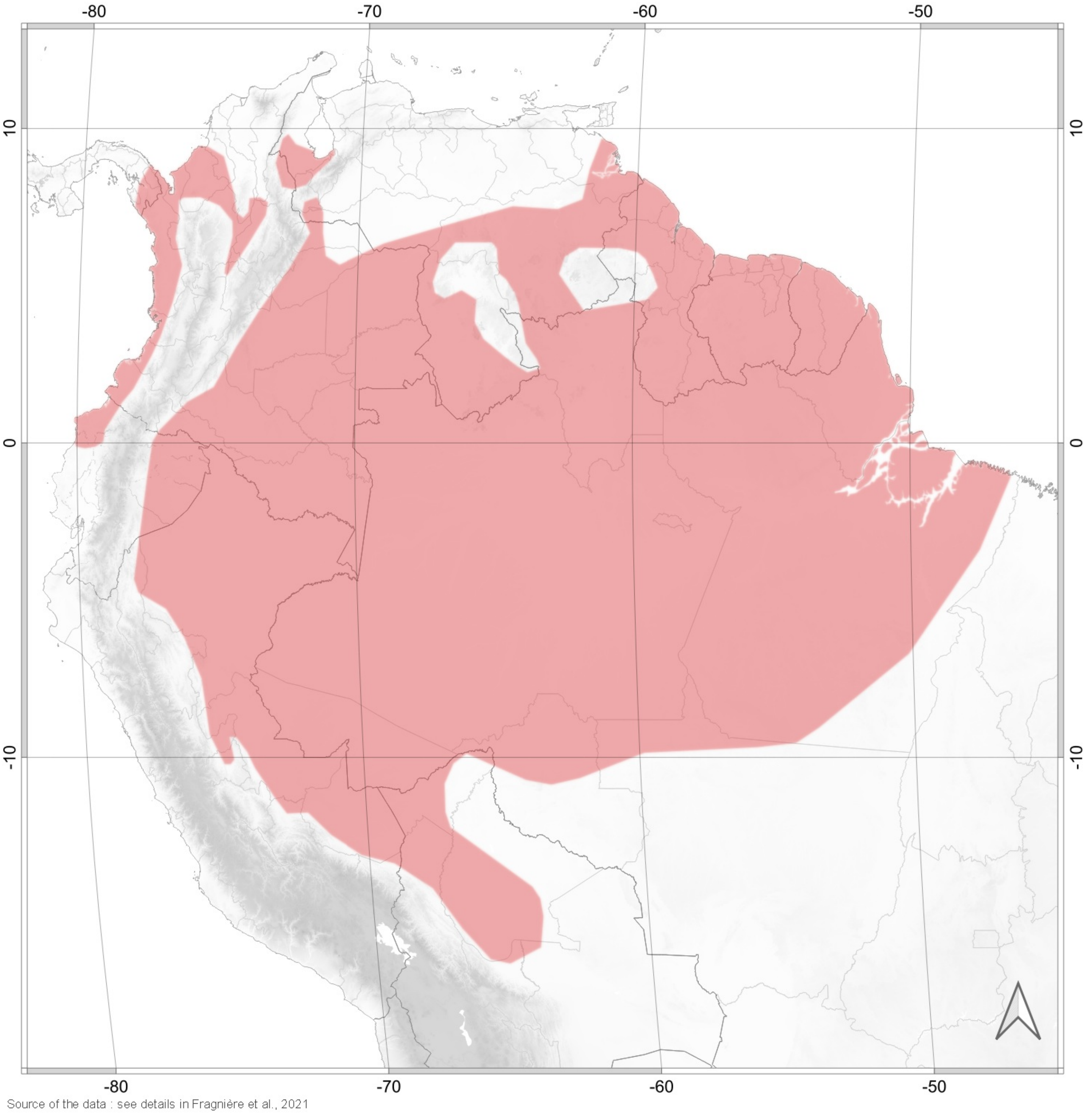

Source of the data : see details in Fragnière et al., 2021

Ampelocera glabra

Kuhlm.

Arch. Jard. Bot. Rio de Janeiro 4: 351, pl. 28 (1925)

ULMACEAE

IUCN Red list status : ne
*
habitat-ecology : dense primary forests
*
climate - Köppen classification : Af, Aw
*
indicative altitudinal range : 0 - 800 m
*
min. latitude : -23.1, max. latitude : -14.8,
min. longitude : -43.9, max. longitude : -38.9

min+ max elevation estimated from distribution

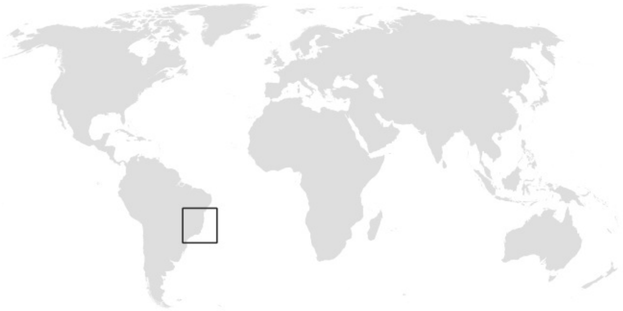

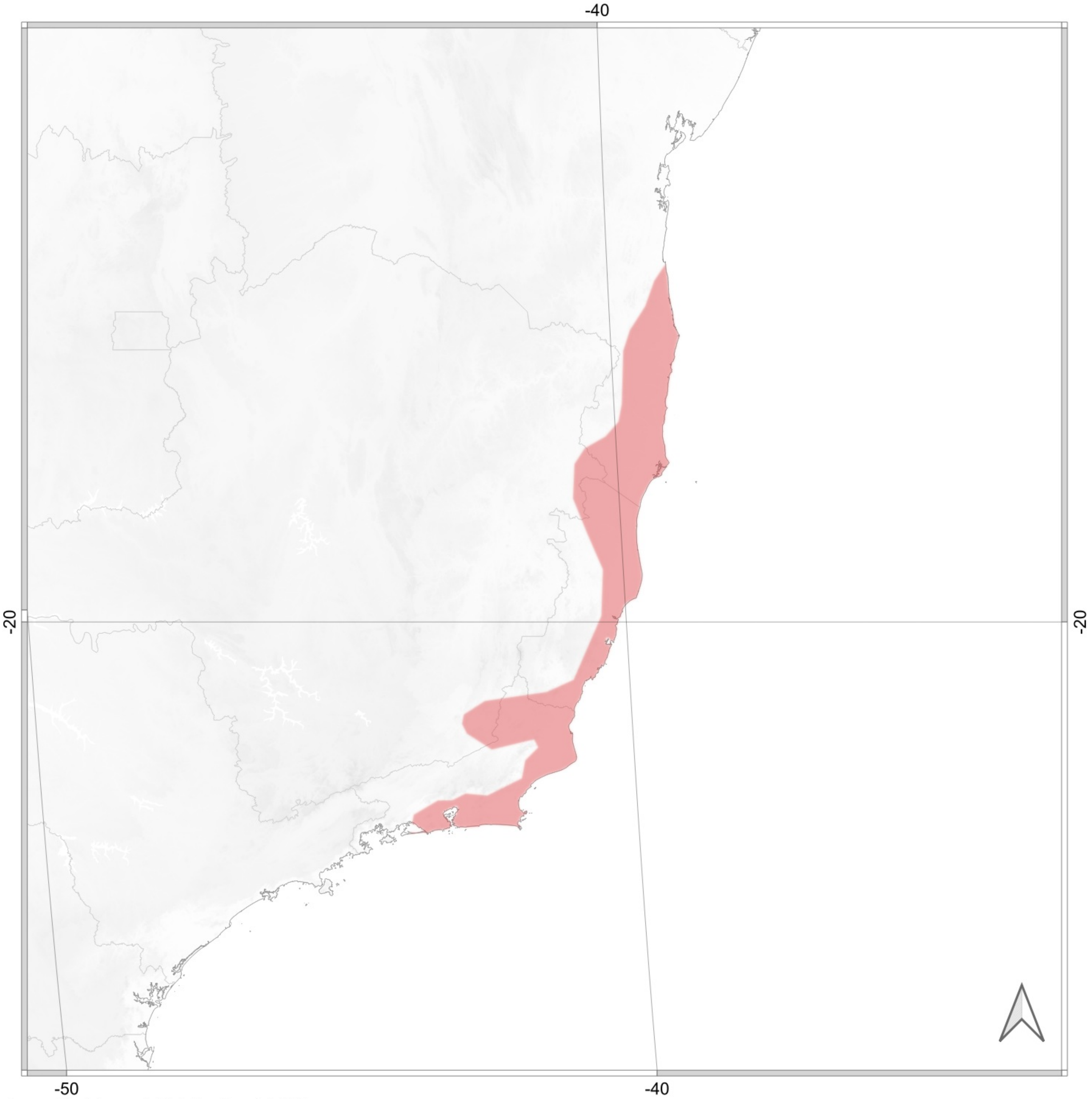

Source of the data : see details in Fragnière et al., 2021

Ampelocera hottlei

Standl.

Trop. Woods 51: 11 (1937)

ULMACEAE

IUCN Red list status : LC
*
habitat-ecology : well developed humid forests
*
climate - Köppen classification : Af, Am (Aw)
*
indicative altitudinal range : 0 - 800 m
*
min. latitude : 9.3, max. latitude : 20.9,
min. longitude : -97.8, max. longitude : -82.3

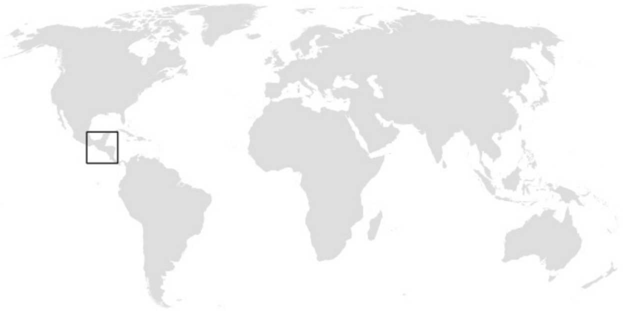

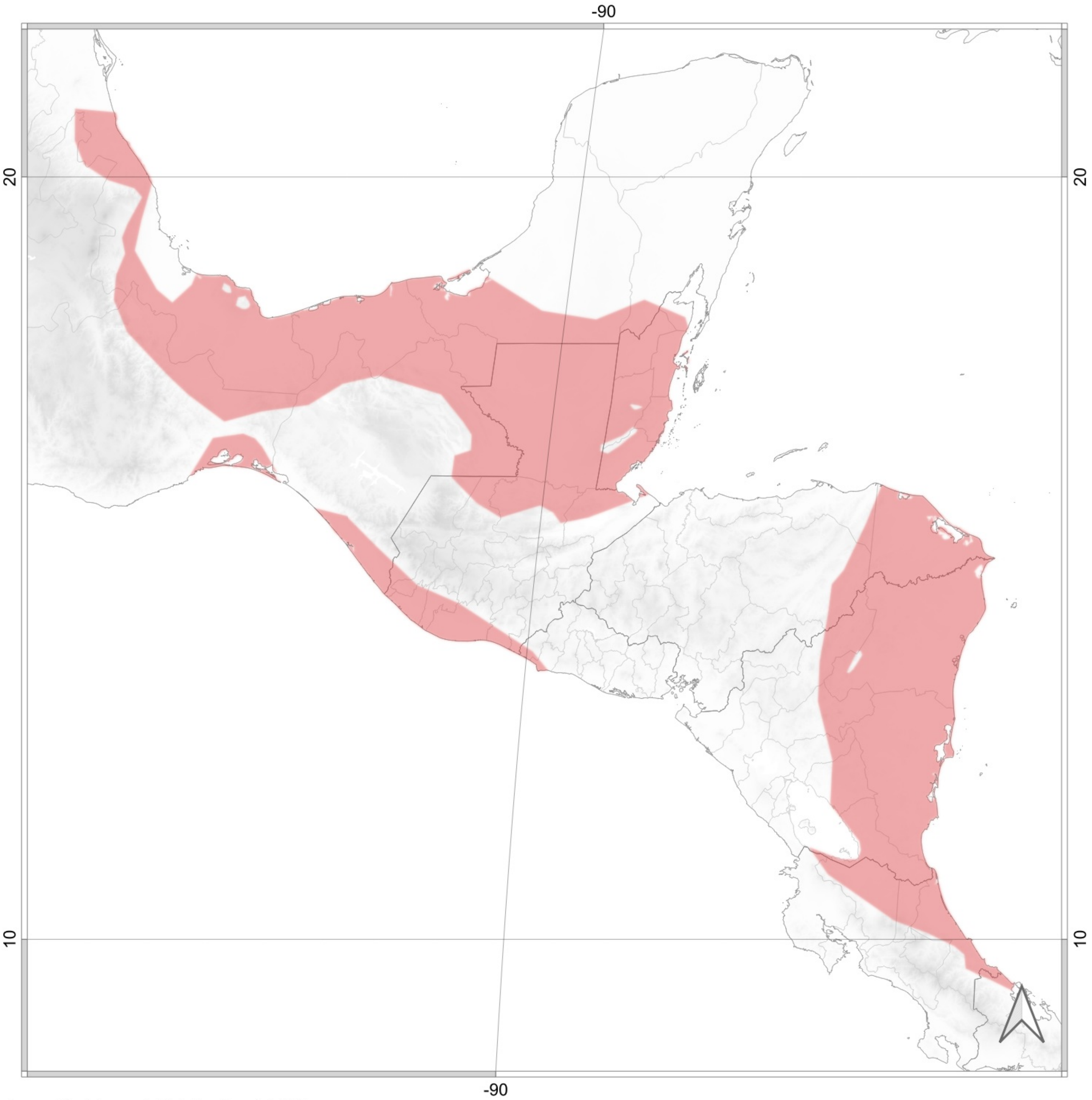

Source of the data : see details in Fragnière et al., 2021

Ampelocera longissima

ULMACEAE

Todzia

Ann. Missouri Bot. Gard. 76: 1096 (-1097), fig. 5 (1989)

IUCN Red list status : NT
*
habitat-ecology : primary forests
*
climate - Köppen classification : Af
*
indicative altitudinal range : 250 - 1500 m
*
min. latitude : -9.1, max. latitude : 8.2,
min. longitude : -77.9, max. longitude : -72.8

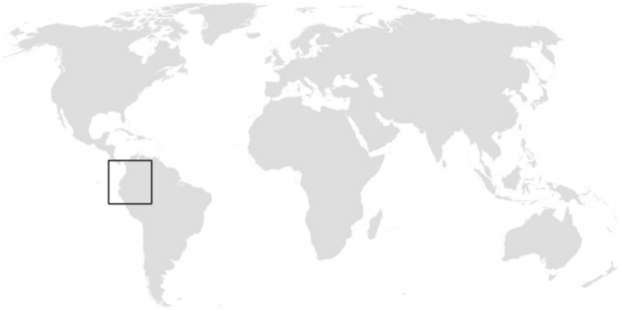

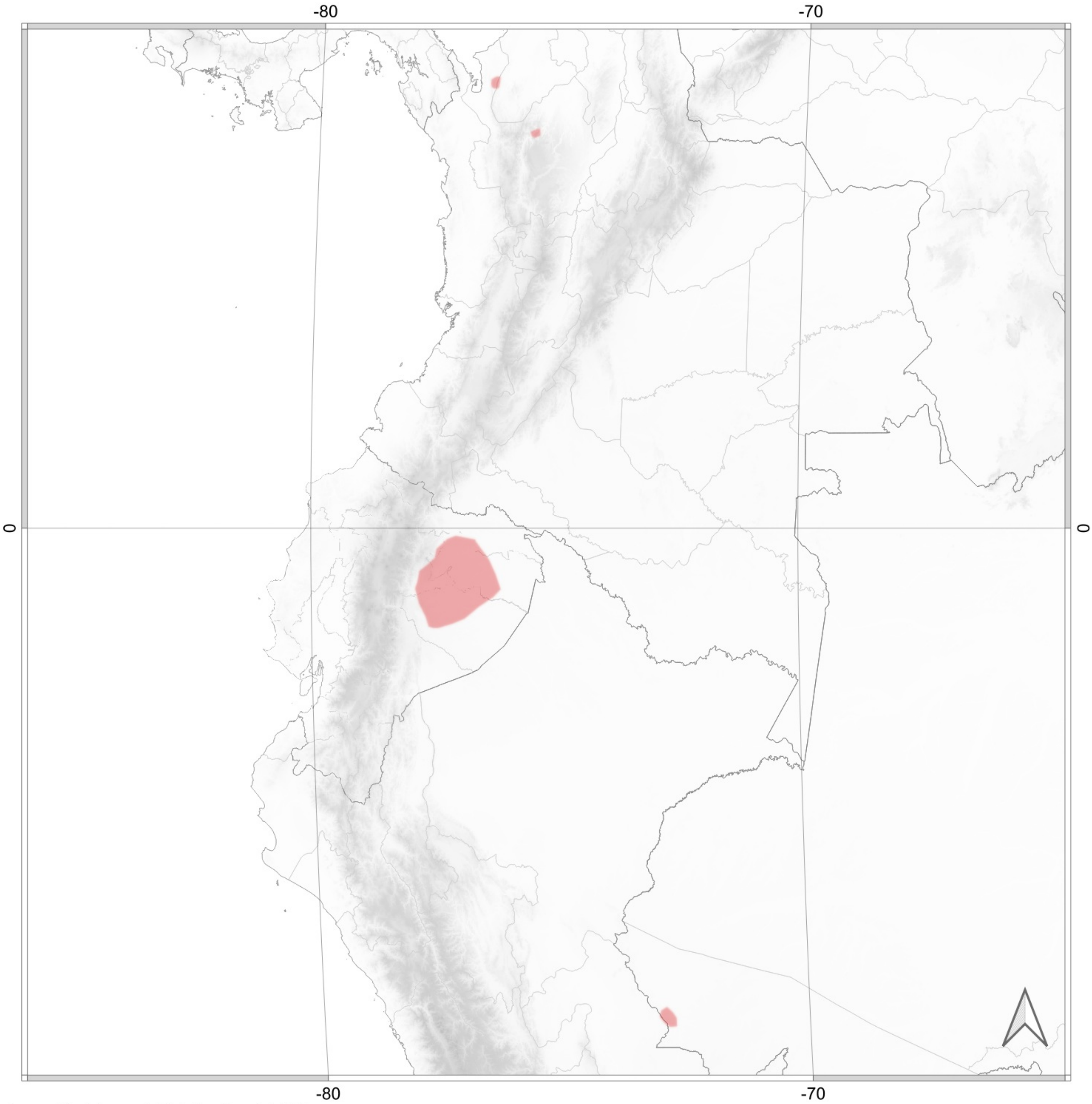

Source of the data : see details in Fragnière et al., 2021

Ampelocera macphersonii

ULMACEAE

Todzia

Ann. Missouri Bot. Gard. 76: 1099, fig. 6 (1989)

IUCN Red list status : LC
*
habitat-ecology : undisturbed deciduous forests and moist forests
*
climate - Köppen classification : Af, Am, Aw
*
indicative altitudinal range : 0 - 1400 m
*
min. latitude : 4.3, max. latitude : 11.5,
min. longitude : -80.8, max. longitude : -70.8

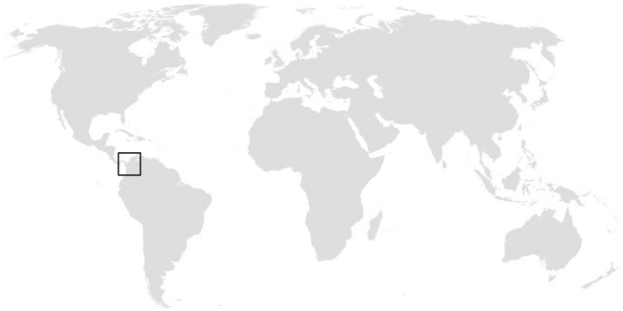

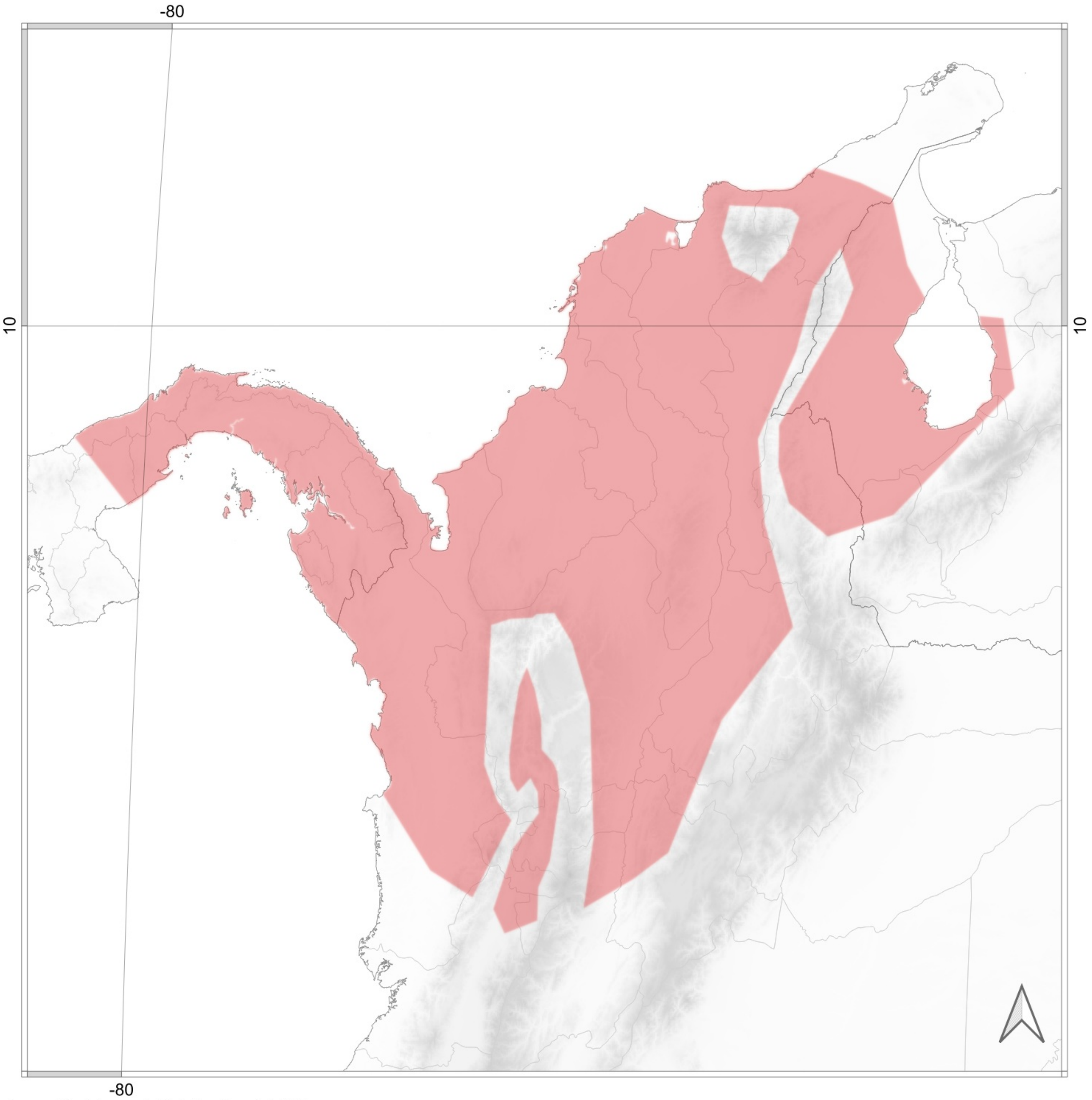

Source of the data : see details in Fragnière et al., 2021

Ampelocera macrocarpa

ULMACEAE

Ferero & Gentry

Phytologia 55: 365 (1984)

IUCN Red list status : LC
*
habitat-ecology : evergreen forests and limestones
*
climate - Köppen classification : Af, Am
*
indicative altitudinal range : 0 - 775 m
*
min. latitude : 3.2, max. latitude : 16,
min. longitude : -87.9, max. longitude : -65.8

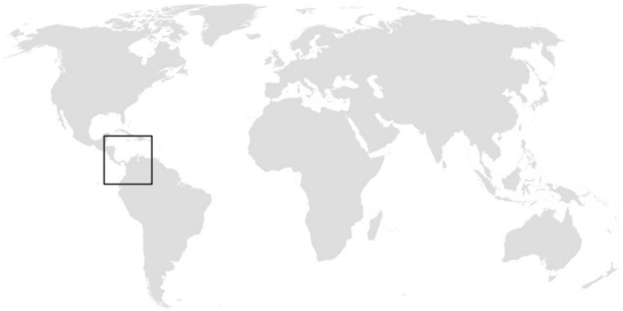

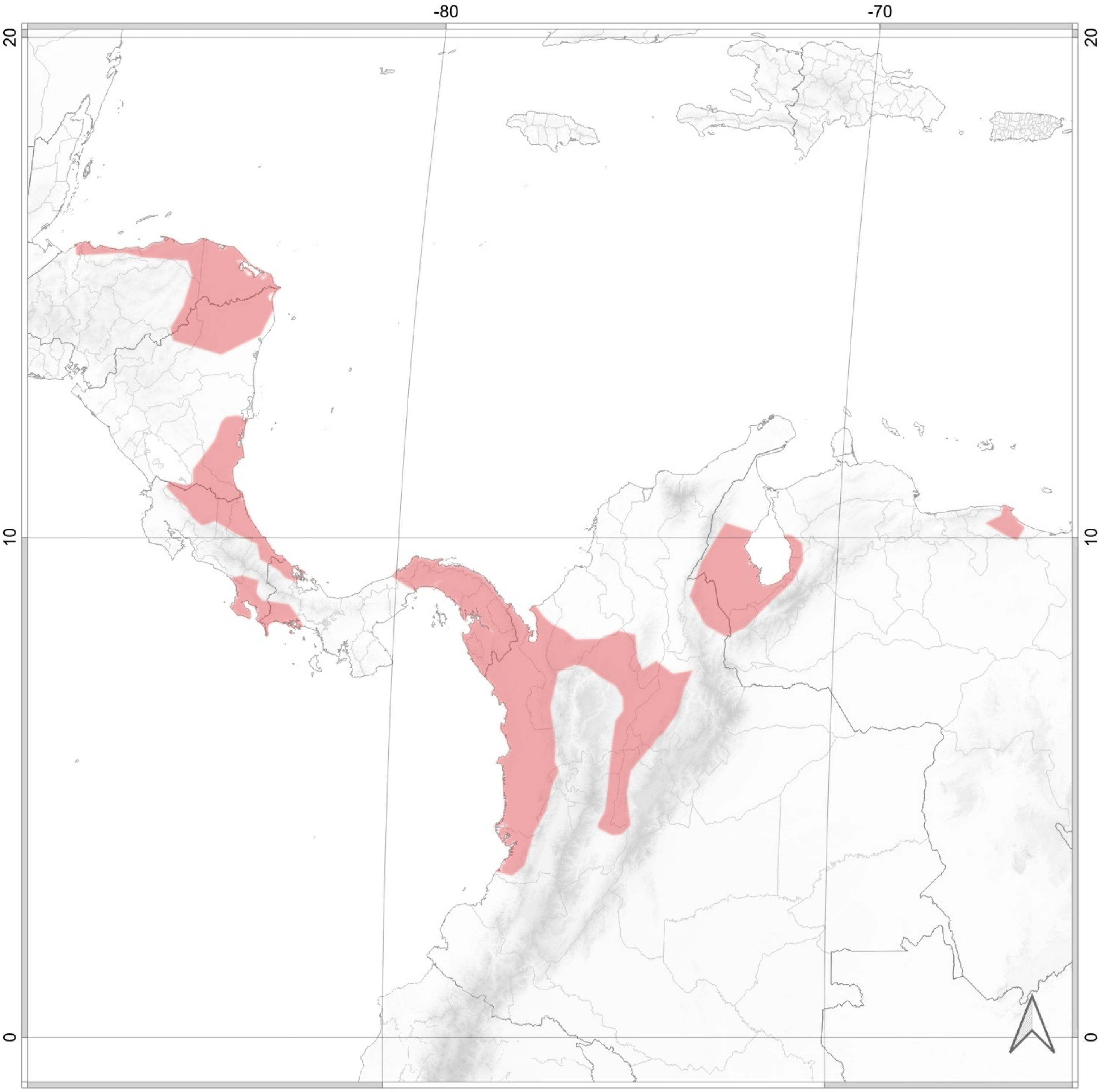

Source of the data : see details in Fragnière et al., 2021

Ampelocera ruizii

ULMACEAE

Klotzsch

Linnaea 20(5): 542 (1847)

IUCN Red list status : LC
*
habitat-ecology : lowland rainforests
*
climate - Köppen classification : Af, Am, (Aw)
*
indicative altitudinal range : 0 - 500 m
*
min. latitude : -18.1, max. latitude : -6.5,
min. longitude : -74.6, max. longitude : -56.8

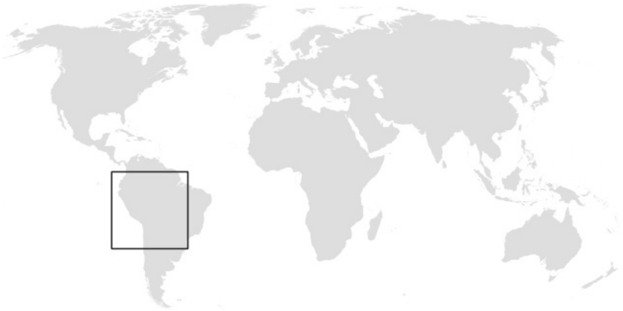

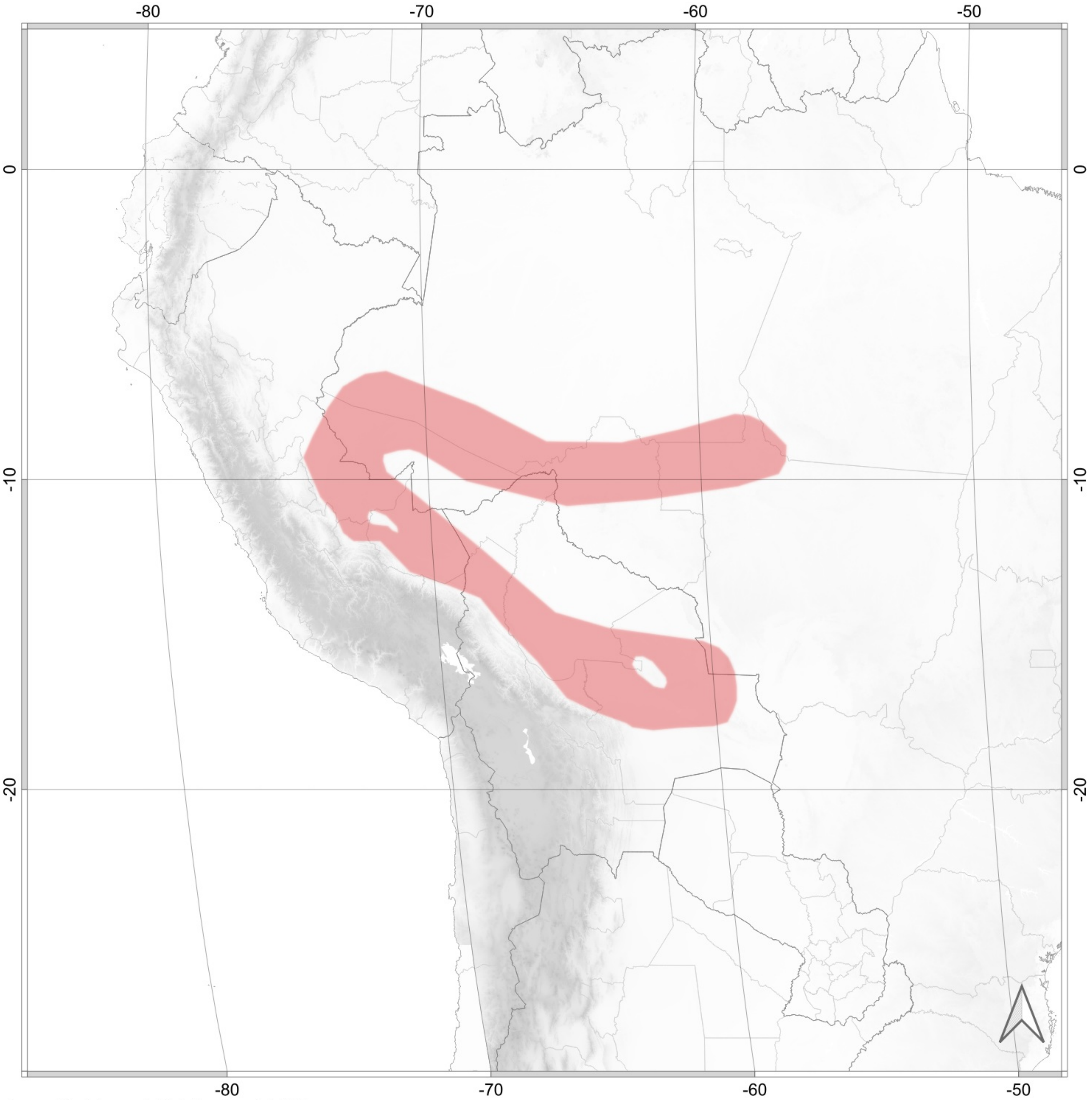

Source of the data : see details in Fragnière et al., 2021

Hemiptelea davidii

(Hance) Planch.

Compt. Rend. Hebd. Séances Acad. Sci. 74: 131 (1872)

ULMACEAE

IUCN Red list status : LC
*
habitat-ecology : hill slopes, trail sides, planted around houses
*
climate - Köppen classification : Dwa, Dwb, Cfa, Cfb, (BSk), (Dfa), (Dwc)
*
indicative altitudinal range : 0 - 2000 m
*
min. latitude : 24.5, max. latitude : 49.4,
min. longitude : 105.2, max. longitude : 130.7

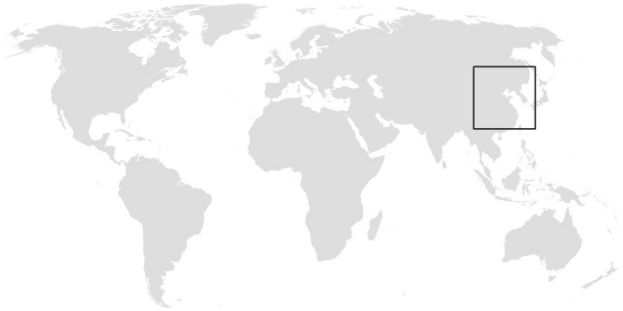

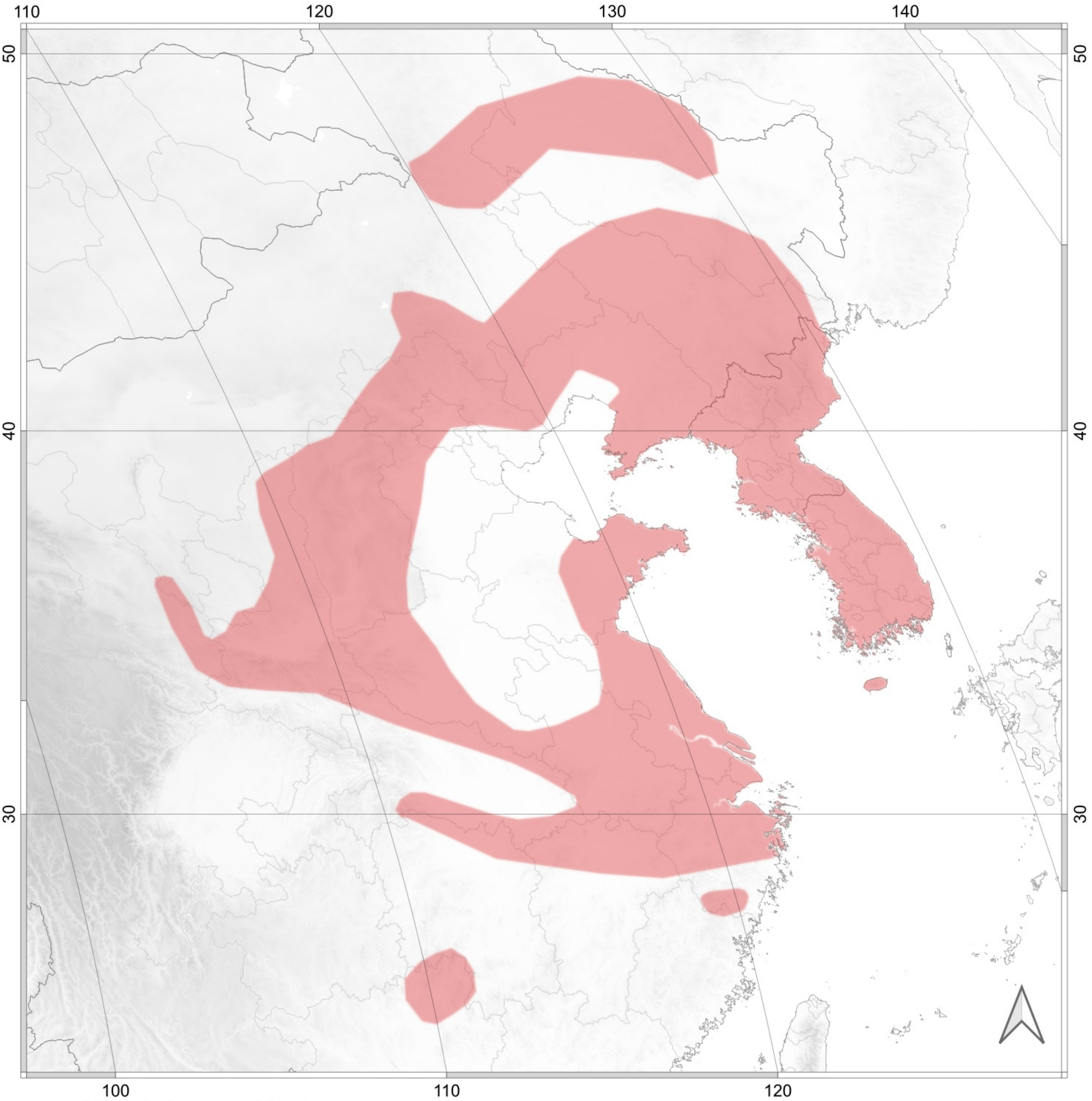

Source of the data : see details in Fragnière et al., 2021

Holoptelea grandis

(Hutch.) Mildbr.

Notizbl. Bot. Gart. Berlin-Dahlem 8: 53 (1921)

ULMACEAE

IUCN Red list status : LC

*

habitat-ecology : rain-forests, drier deciduous and riverine forests, patches of rain-forests in savanna zone

*

climate - Köppen classification : Aw, Am, (Af)

*

indicative altitudinal range : 0 - 1200 m

*

min. latitude : -6.3, max. latitude : 11.4,
min. longitude : -9.3, max. longitude : 31.9

higher elevation in Uganda (>1400m)

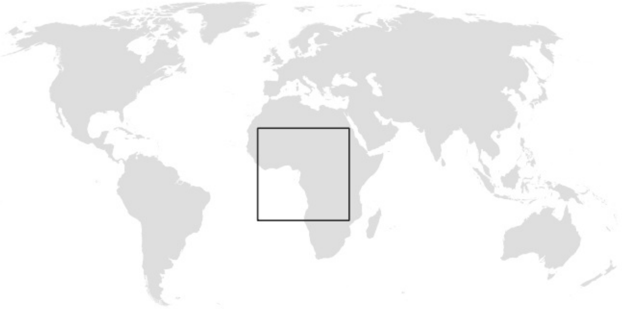

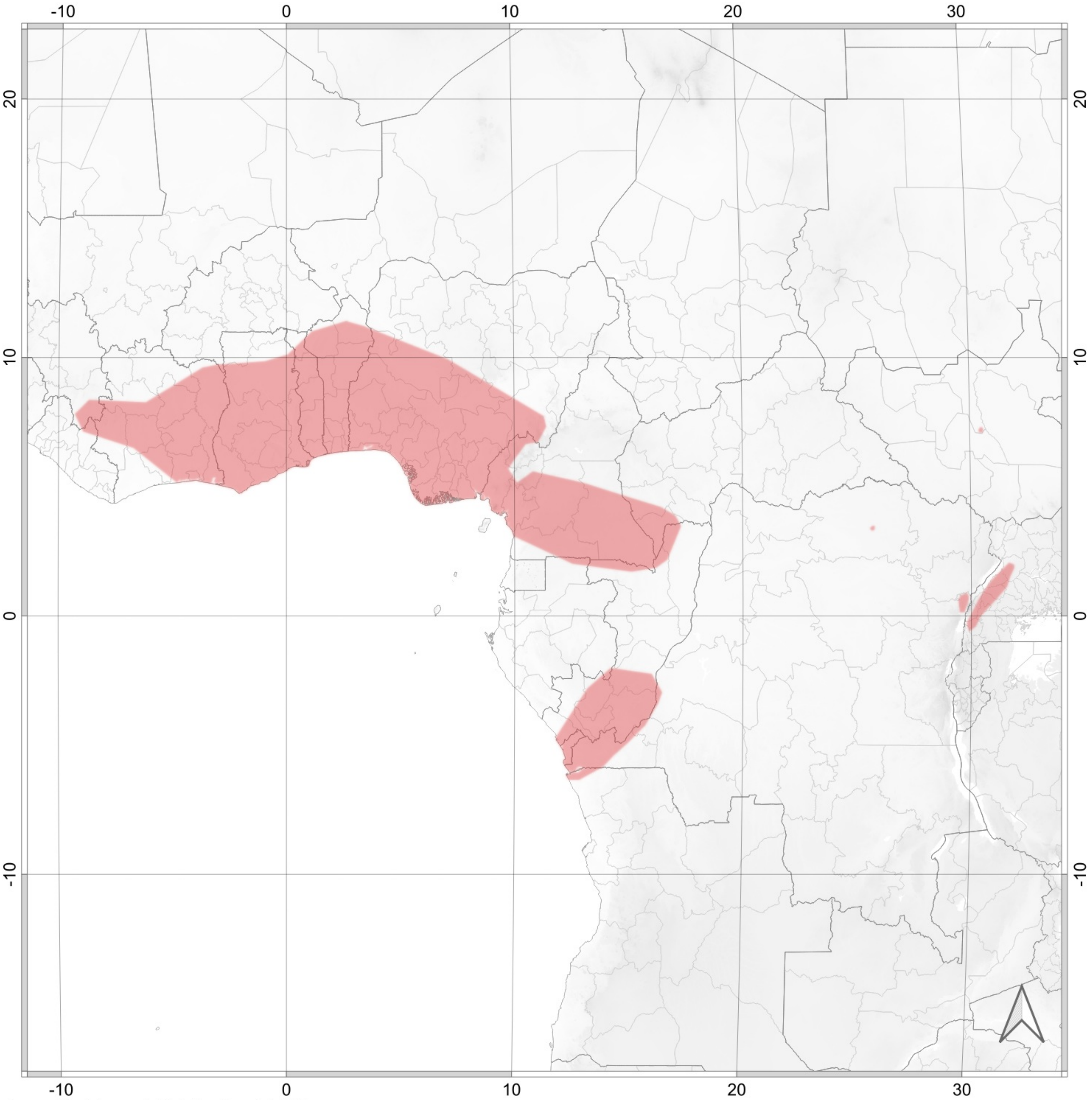

Source of the data : see details in Fragnière et al., 2021

Holoptelea integrifolia

(Roxb.) Planch.

Ann. Sci. Nat., Bot. sér. 3, 10: 259 (1848)

ULMACEAE

IUCN Red list status : ne

*

habitat-ecology : dry deciduous forests, deforested areas, near town and villages, cultivated areas

*

climate - Köppen classification : Aw, BSh, Cwa, Am

*

indicative altitudinal range : 0 - 800 m

*

min. latitude : 7.5, max. latitude : 32.5,
min. longitude : 70.2, max. longitude : 107.9

distribution not precise, lacking good references

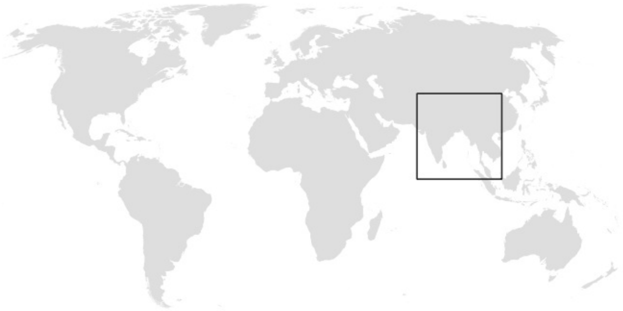

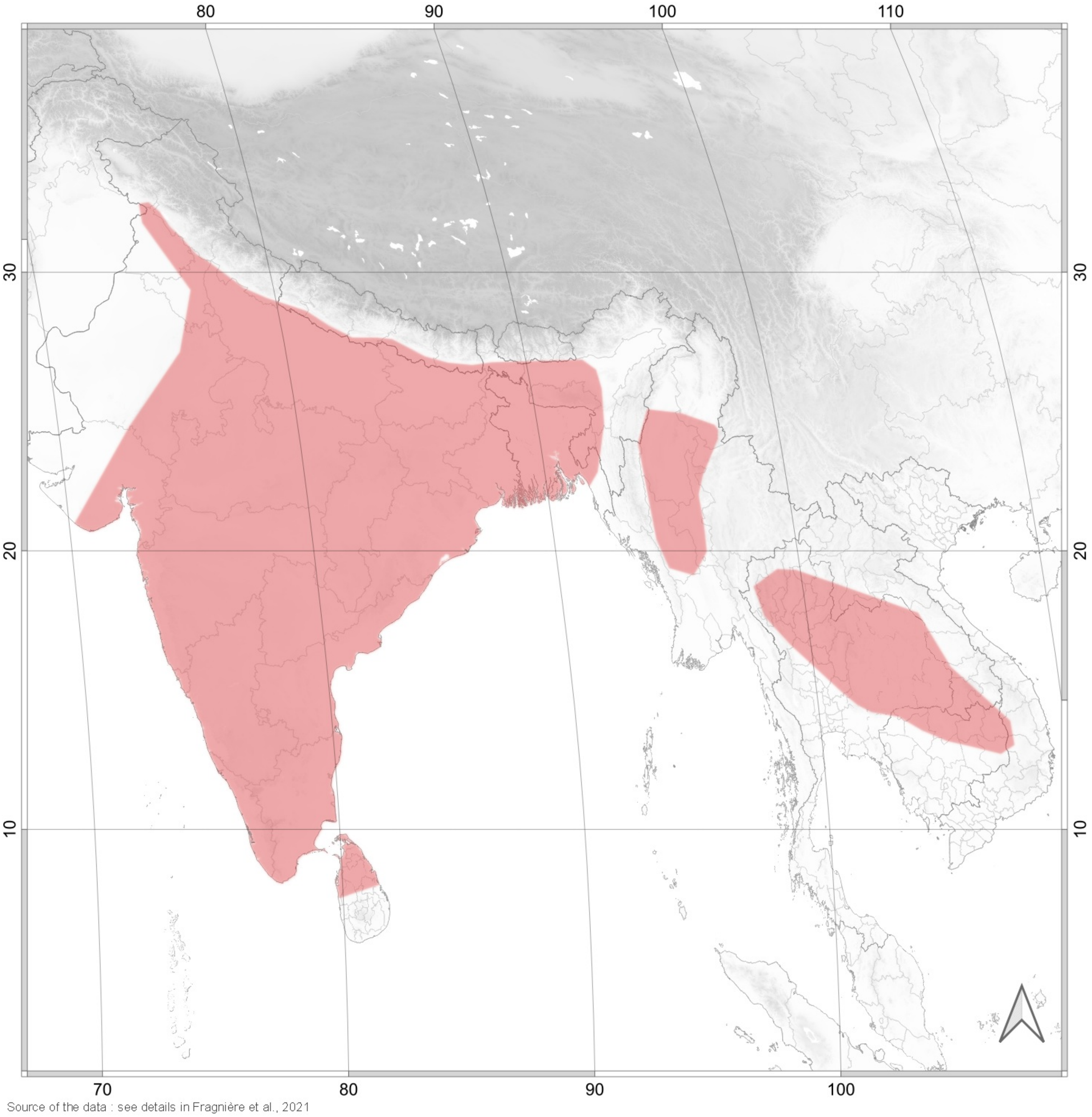

Source of the data : see details in Fragnière et al., 2021

Phyllostylon brasiliense

Capan. ex Benth. & Hook. f.

Gen. Pl. 3: 352 (1880)

ULMACEAE

IUCN Red list status : ne
*
habitat-ecology : ombrophylous forests
*
climate - Köppen classification : Af, Am, Aw
*
indicative altitudinal range : 0 - 1300 m
*
min. latitude : -23.4, max. latitude : -7.9,
min. longitude : -45.8, max. longitude : -34.8

distribution varies between sources. min+ max elevation
estimated from distribution

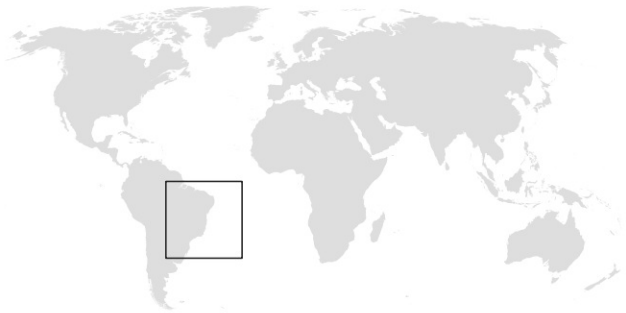

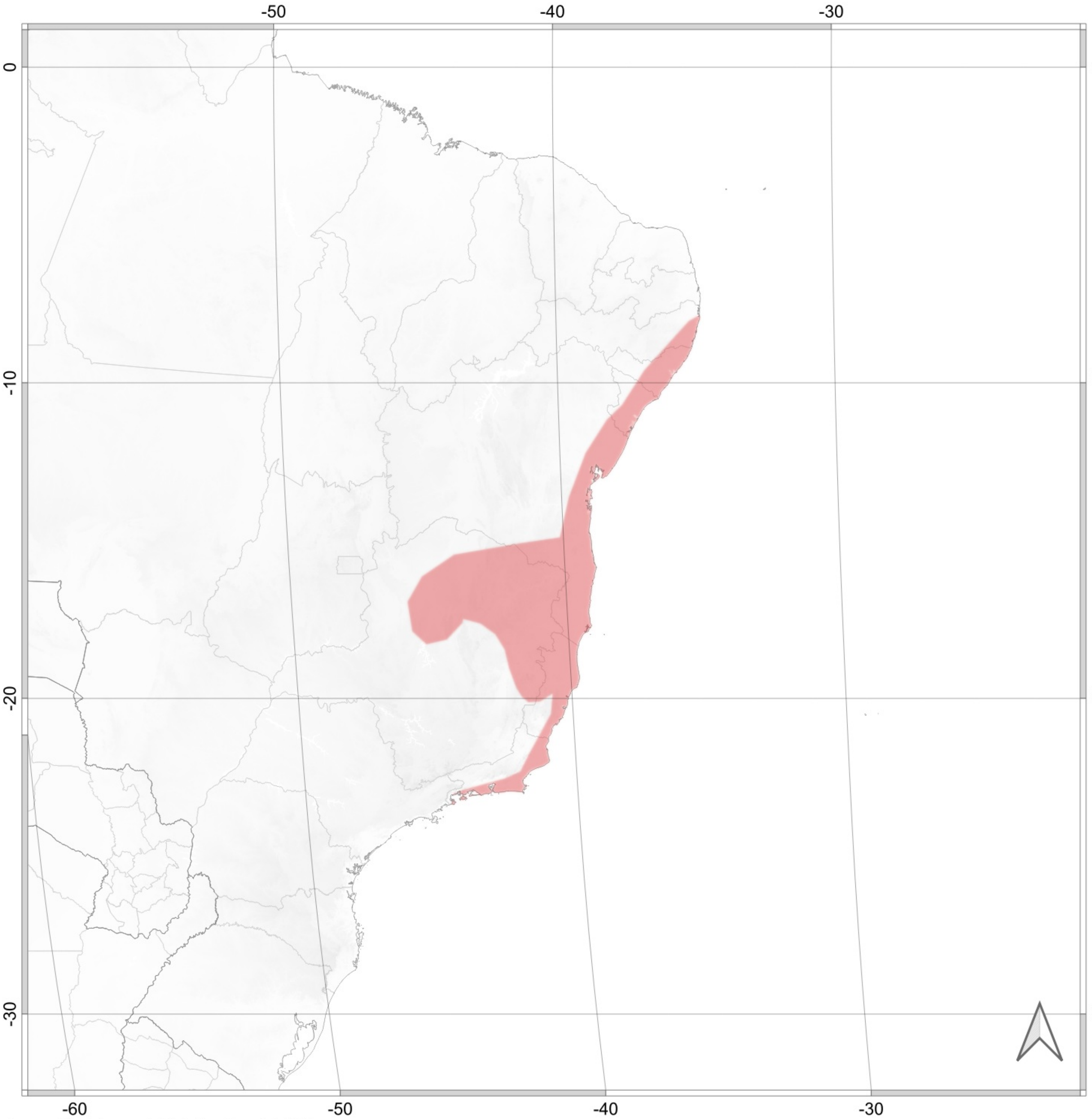

Source of the data : see details in Fragnière et al., 2021

Phyllostylon rhamnoides

(J.Poiss.) Taub

Oesterr. Bot. Z. 40: 409 (1890)

ULMACEAE

IUCN Red list status : LC
*
habitat-ecology : variety of habitat in dry tropical forest zones.
Scrub forests, moist forests, open savannas
*
climate - Köppen classification : Aw, BSh, (Cwa, Cfa)
*
indicative altitudinal range : 0 - 1500 m
*
min. latitude : -29.5, max. latitude : 24.4,
min. longitude : -105.2, max. longitude : -48.7

min+ max elevation estimated from distribution

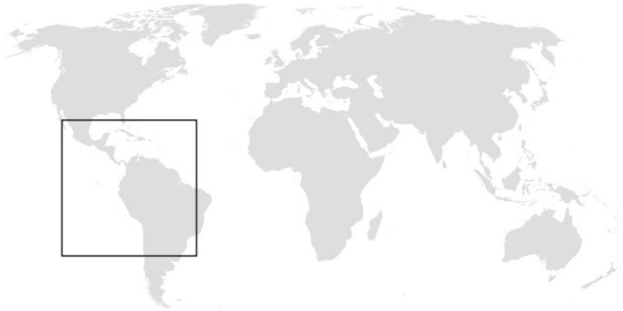

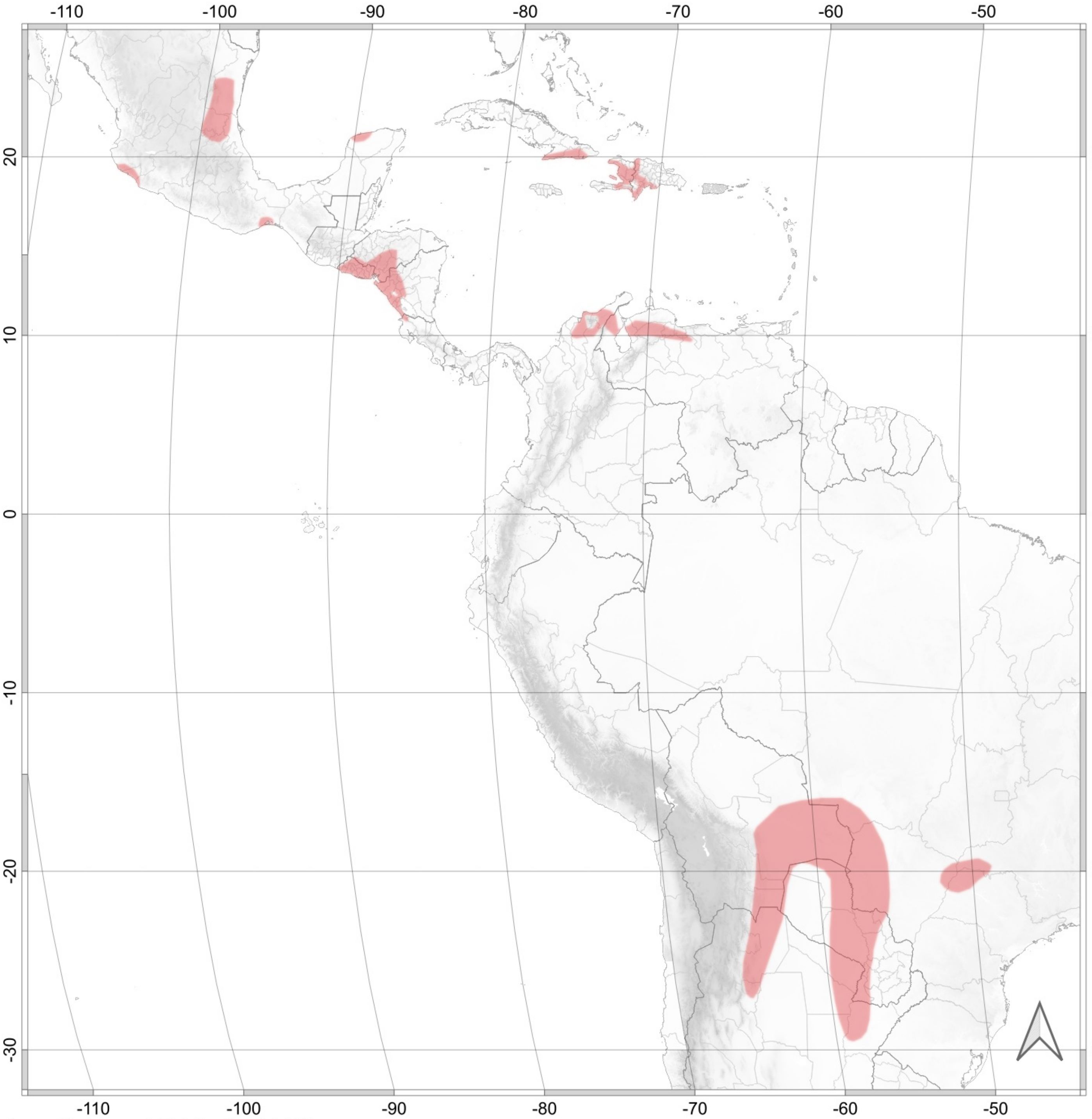

Source of the data : see details in Fragnière et al., 2021

Planera aquatica

ULMACEAE

J.F.Gmel.

Syst. Nat., ed. 13bis. 2(1): 150 (1791)

IUCN Red list status : LC
*
habitat-ecology : swamps, streams, lakes, alluvial flood plains
*
climate - Köppen classification : Cfa
*
indicative altitudinal range : 0 - 200 m
*
min. latitude : 29, max. latitude : 37.6,
min. longitude : -97.1, max. longitude : -78.2

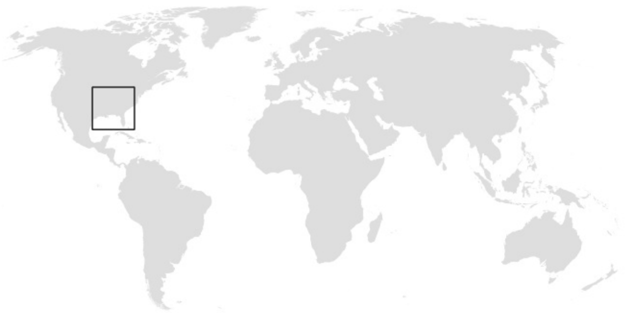

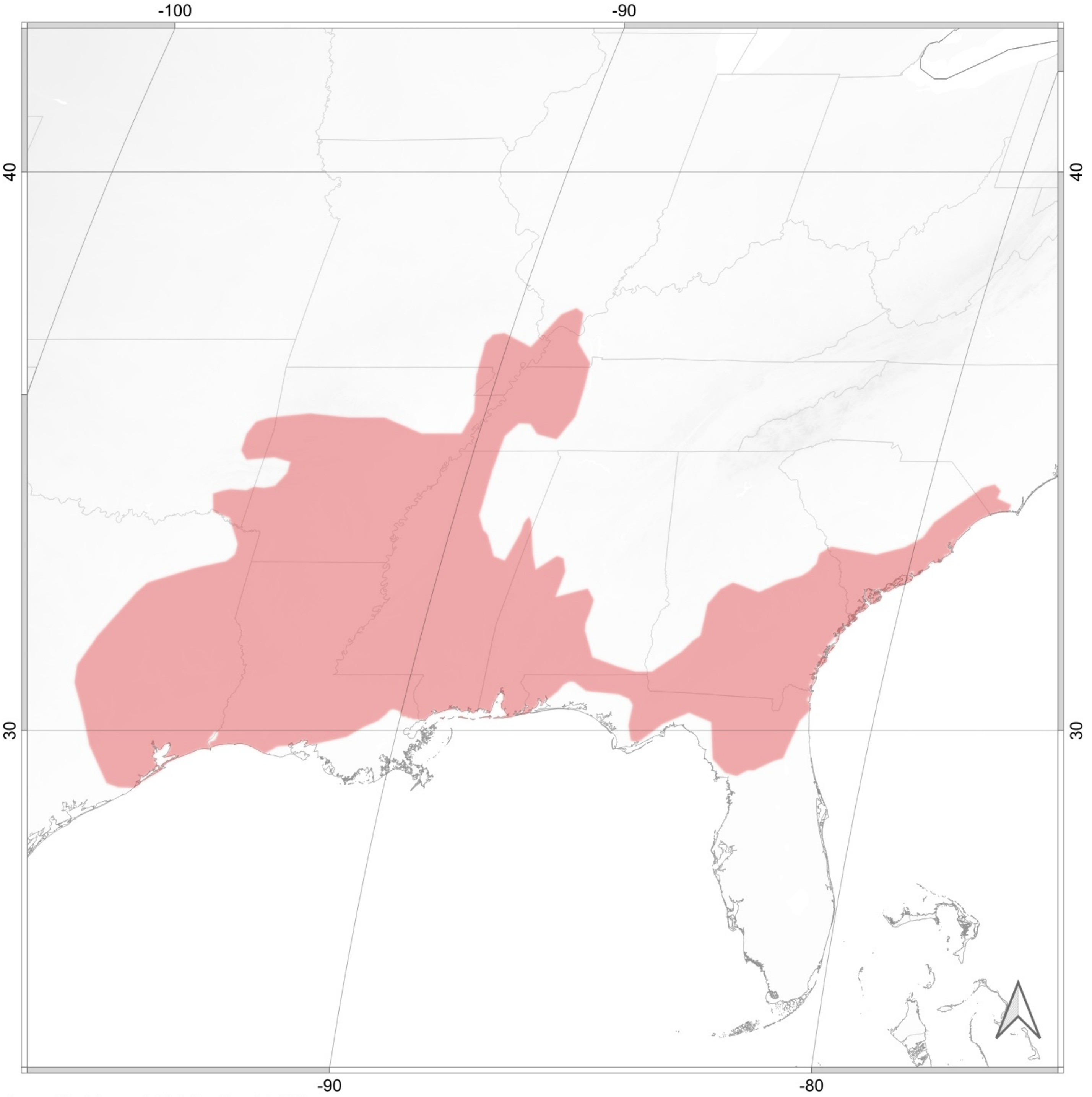

Source of the data : see details in Fragnière et al., 2021

Ulmus alata

ULMACEAE

Michx.

Fl. Bor.-Amer. (Michaux) 1: 173 (1803)

IUCN Red list status : LC
*
habitat-ecology : alluvial woods and deciduous woodlands, especially dry, acidic woodlands and glades, along fencerows, waste areas
*
climate - Köppen classification : Cfa, (Dfa)
*
indicative altitudinal range : 0 - 600 m
*
min. latitude : 27.1, max. latitude : 38.9,
min. longitude : -97.7, max. longitude : -75.5

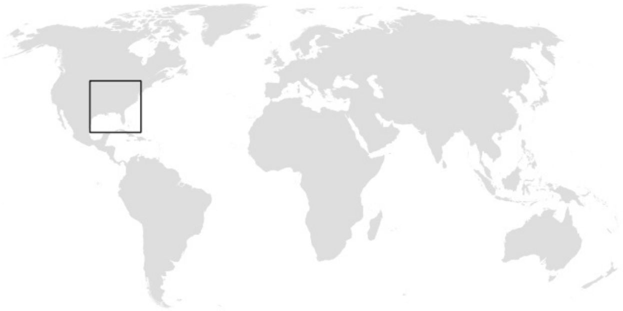

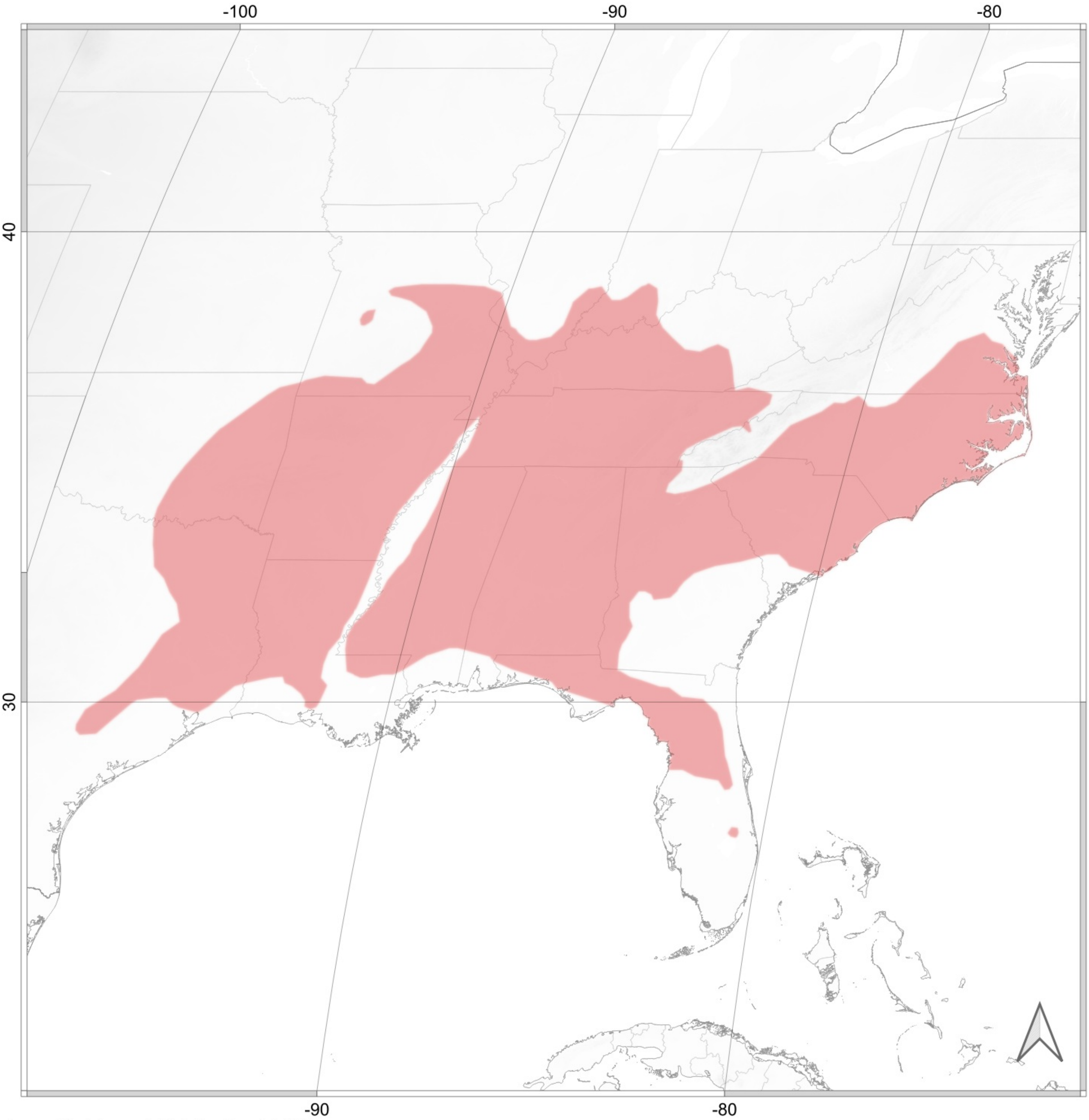

Source of the data : see details in Fragnière et al., 2021

Ulmus americana

L.

Sp. Pl. 1: 226 (1753)

ULMACEAE

IUCN Red list status : EN

*

habitat-ecology : alluvial woods, swamp forests, deciduous woodlands, fencerows, pastures, old fields

*

climate - Köppen classification : Cfa, Dfa, Dfb

*

indicative altitudinal range : 0 - 1400 m

*

min. latitude : 27.1, max. latitude : 54.1,
min. longitude : -105.8, max. longitude : -59.7

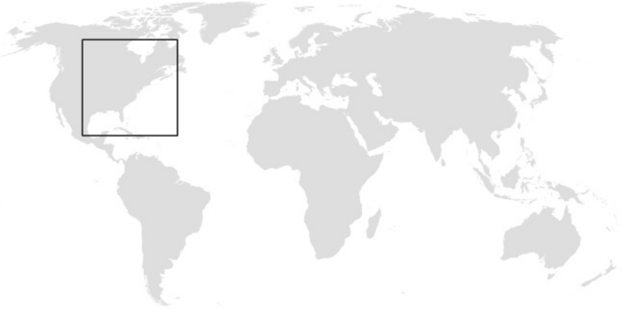

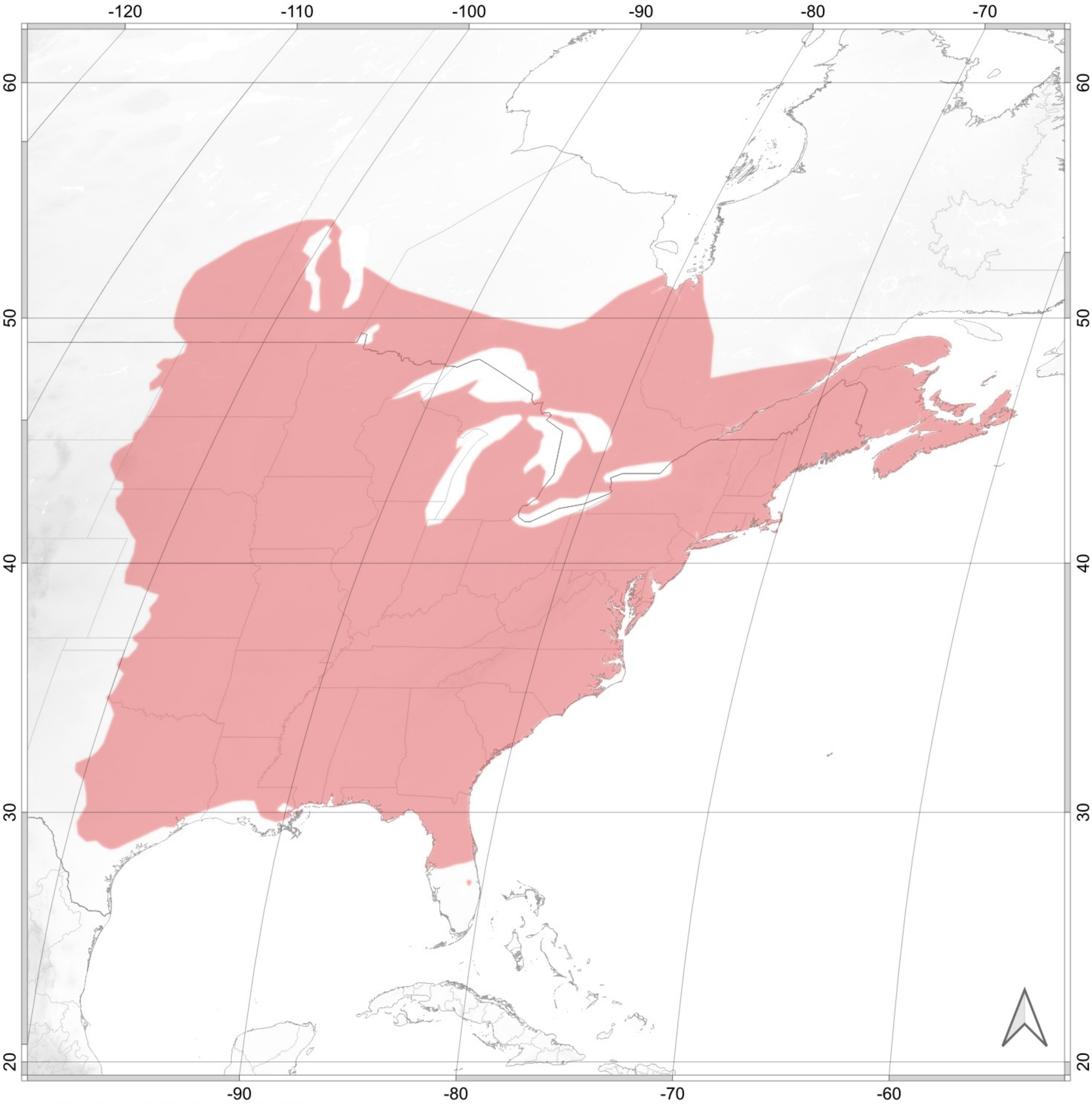

Source of the data : see details in Fragnière et al., 2021

Ulmus androssowii

ULMACEAE

Litv.

Sched. Herb. Fl. Ross. viii. 23 (1922)

IUCN Red list status : ne
*
habitat-ecology : broad-leaved forests on mountain slopes and in valleys
*
climate - Köppen classification : Cwb, Cwa
*
indicative altitudinal range : 1200 - 2800 m
*
min. latitude : 22.7, max. latitude : 33.2,
min. longitude : 75.1, max. longitude : 104.7

synonym : U. chumlia

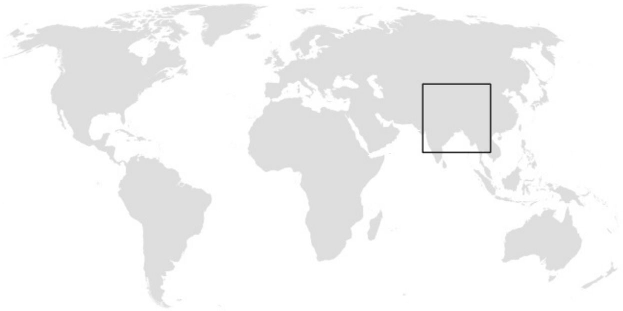

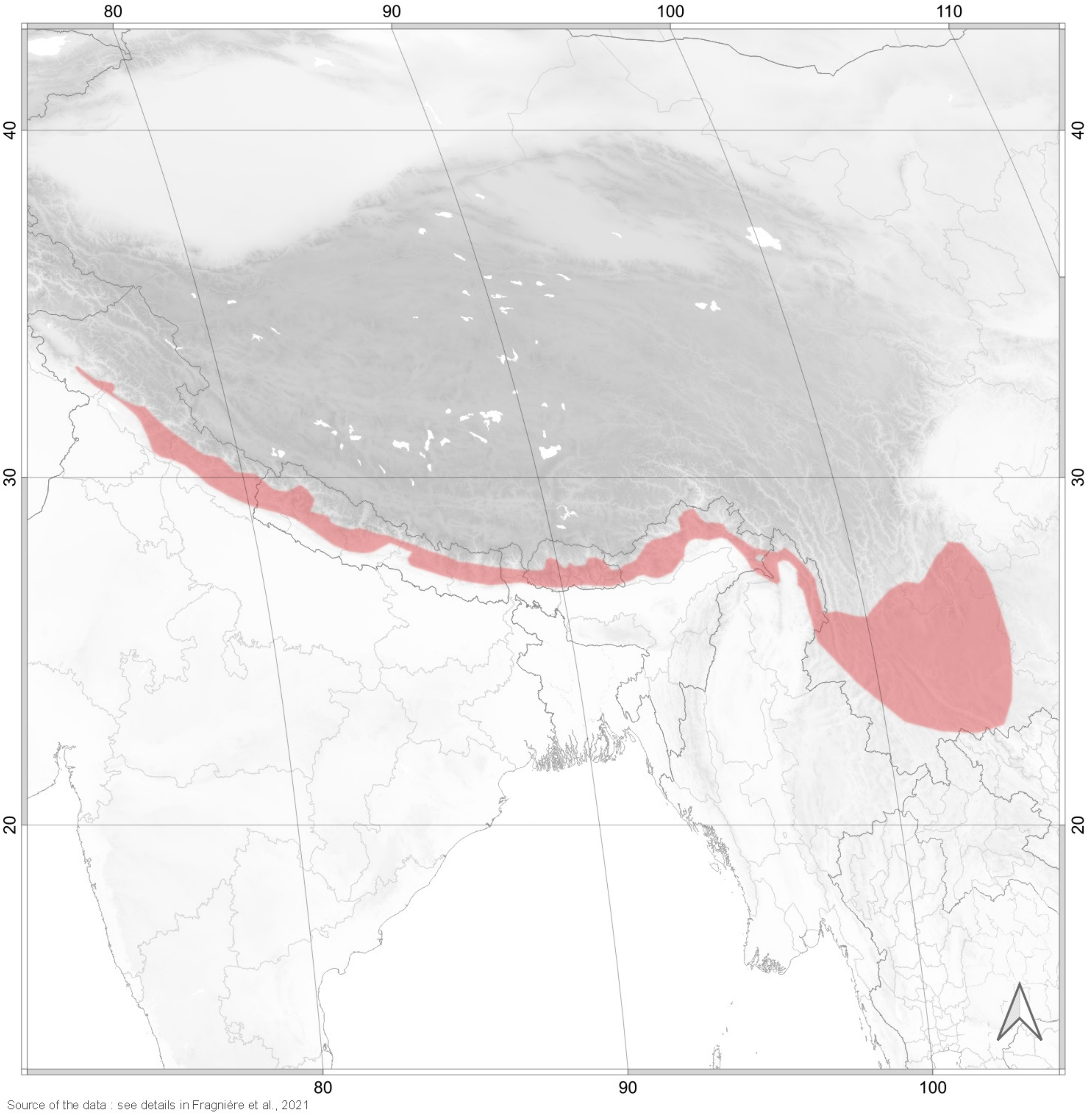

Source of the data : see details in Fragnière et al., 2021

Ulmus bergmanniana

C.K.Schneid.

Ill. Handb. Laubholzk. ii 902 (1912)

ULMACEAE

IUCN Red list status : ne
*
habitat-ecology : forests
*
climate - Köppen classification : Cwb, Cwa, Cfa, Dwa, Dwb
*
indicative altitudinal range : 1500 - 2900 m
*
min. latitude : 24.2, max. latitude : 36.3,
min. longitude : 91, max. longitude : 119.4

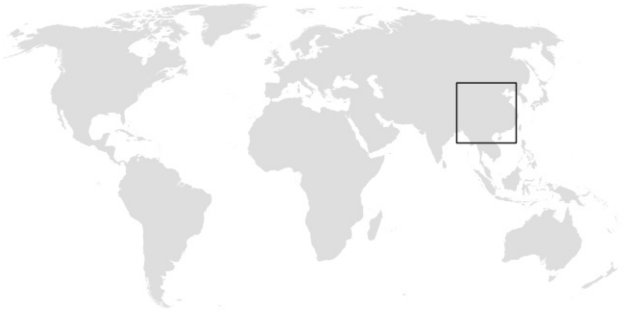

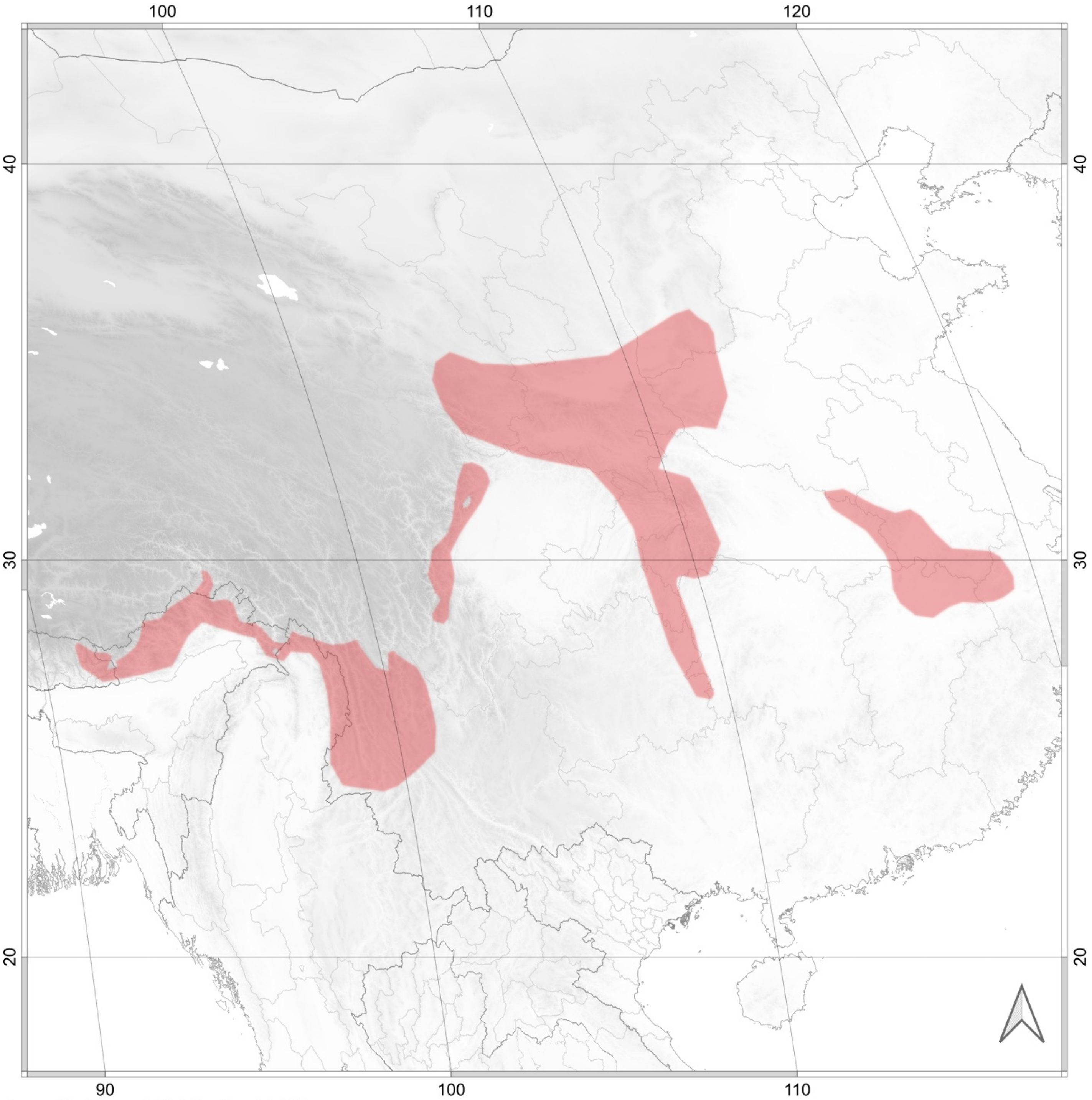

Source of the data : see details in Fragnière et al., 2021

Ulmus castaneifolia

ULMACEAE

Hemsl.

J. Linn. Soc., Bot. 26(177): 446–447, pl. 10 (1894)

IUCN Red list status : LC
*
habitat-ecology : broad-leaved forests
*
climate - Köppen classification : Cfa, Cwa
*
indicative altitudinal range : 500 - 1600 m
*
min. latitude : 23.4, max. latitude : 31,
min. longitude : 104.2, max. longitude : 119.5

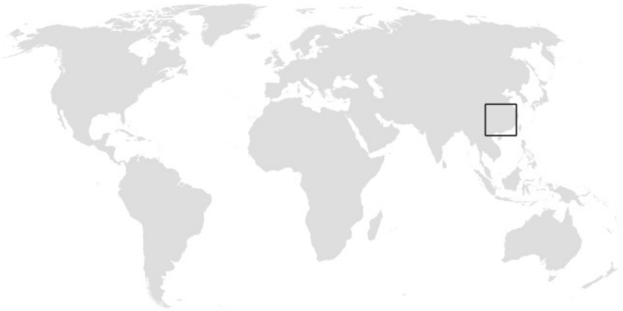

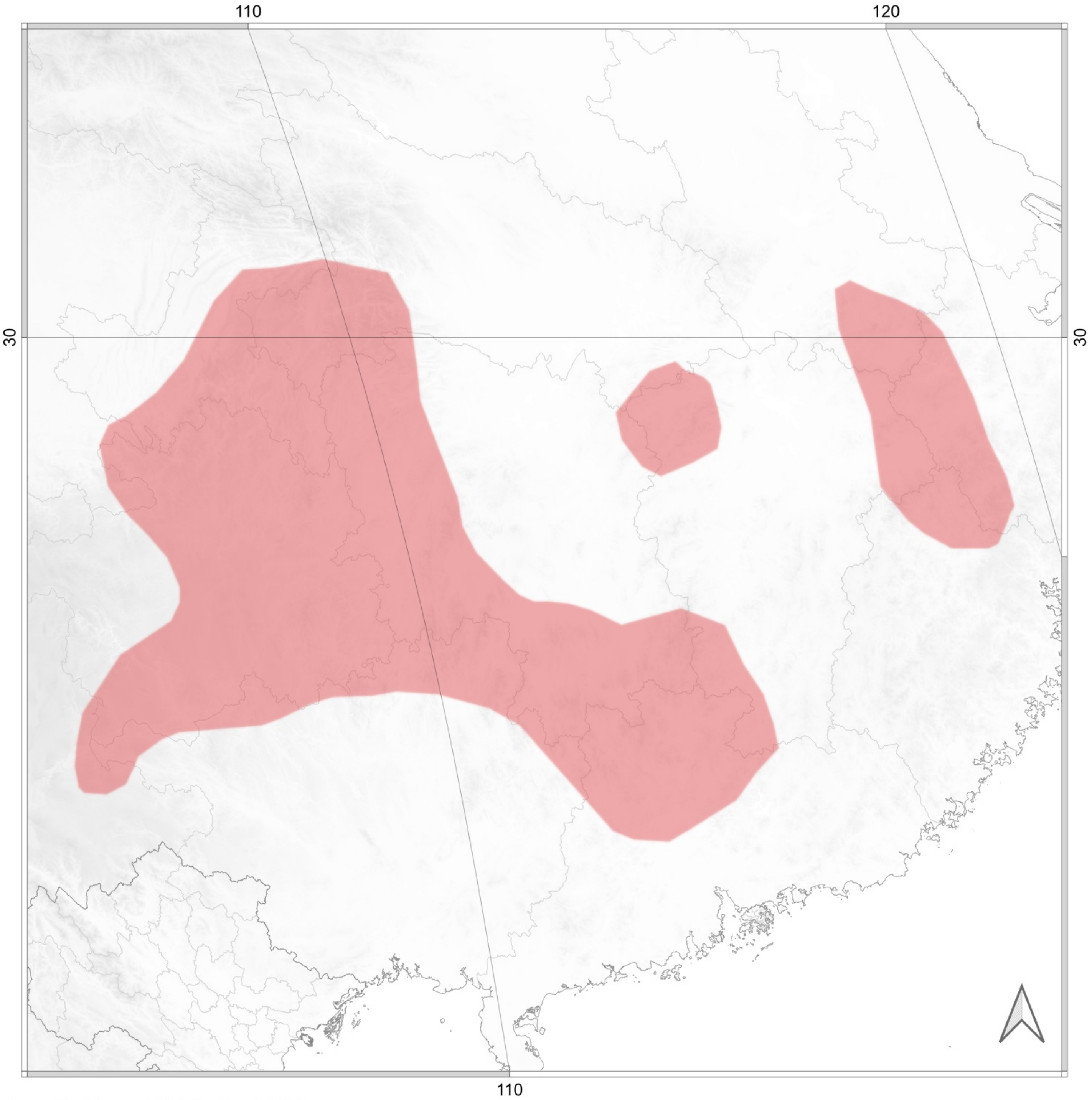

Source of the data : see details in Fragnière et al., 2021

Ulmus changii

W.C.Cheng

Contr. Biol. Lab. Sci. Soc. China, Bot. Ser. 10: 94 (1936)

ULMACEAE

IUCN Red list status : ne
*
habitat-ecology : montane forests
*
climate - Köppen classification : Cfa, (Cwa)
*
indicative altitudinal range : 200 - 1800 m
*
min. latitude : 24.4, max. latitude : 31.6,
min. longitude : 101.9, max. longitude : 119.9

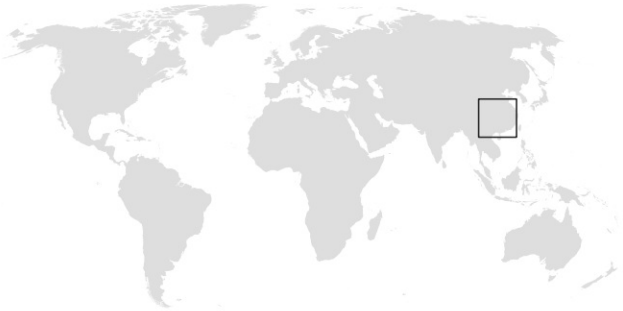

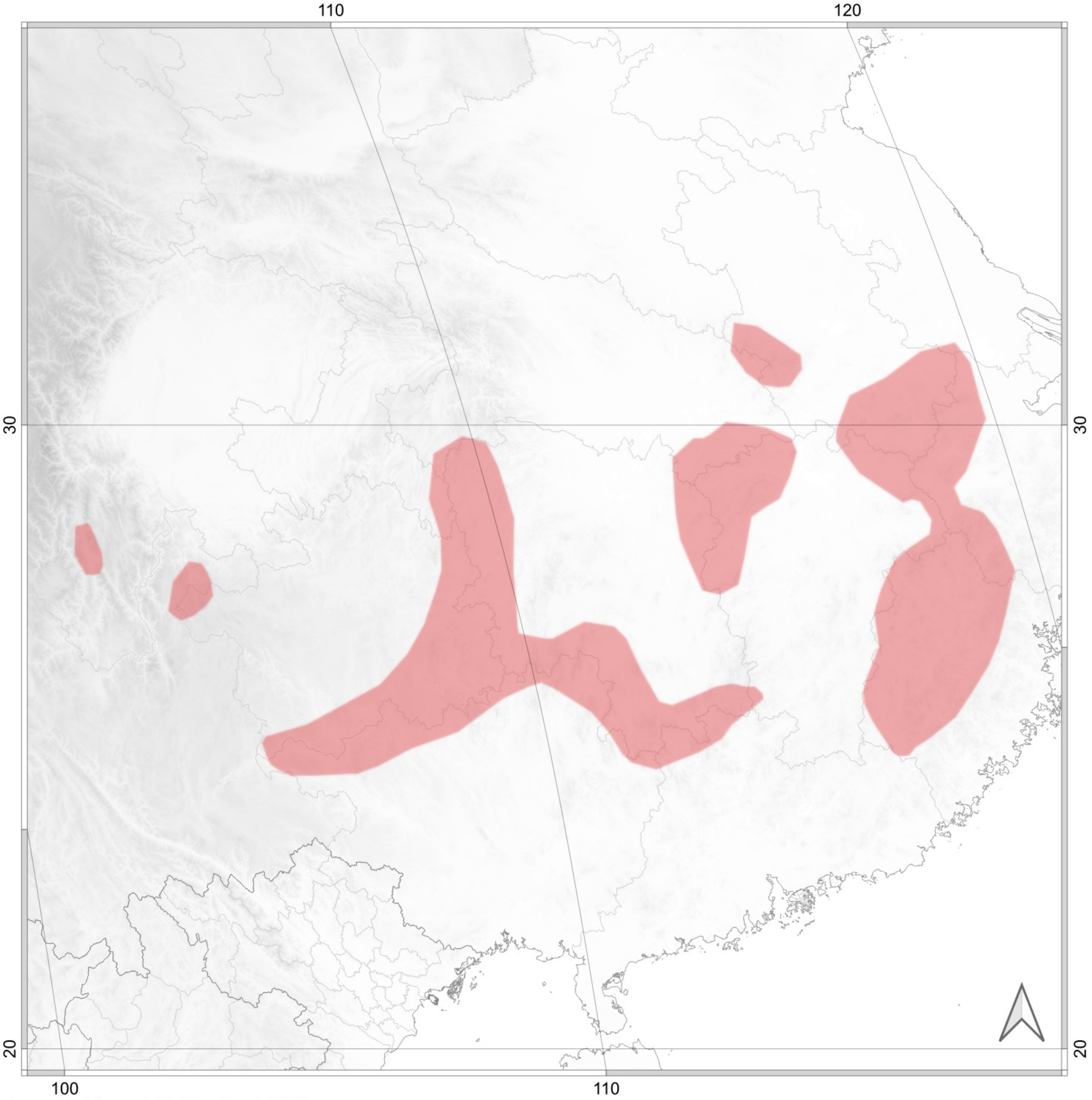

Source of the data : see details in Fragnière et al., 2021

Ulmus chenmoui

W.C.Cheng

J. Nanjing Forest. Coll. 1: 69 (68-70) (1958)

ULMACEAE

IUCN Red list status : EN
*
habitat-ecology : deciduous broadleaved forest on limestone hills
*
climate - Köppen classification : Cfa
*
indicative altitudinal range : 100 - 200 m
*
min. latitude : 31.8, max. latitude : 32.4,
min. longitude : 118.1, max. longitude : 119.3

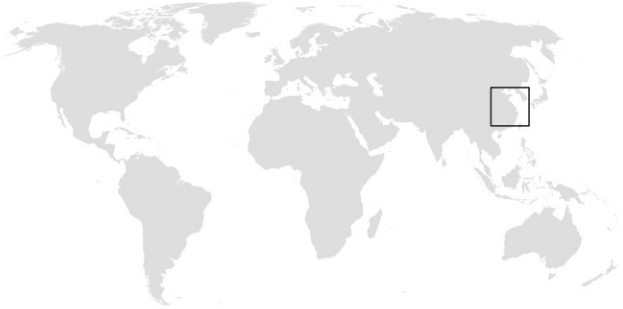

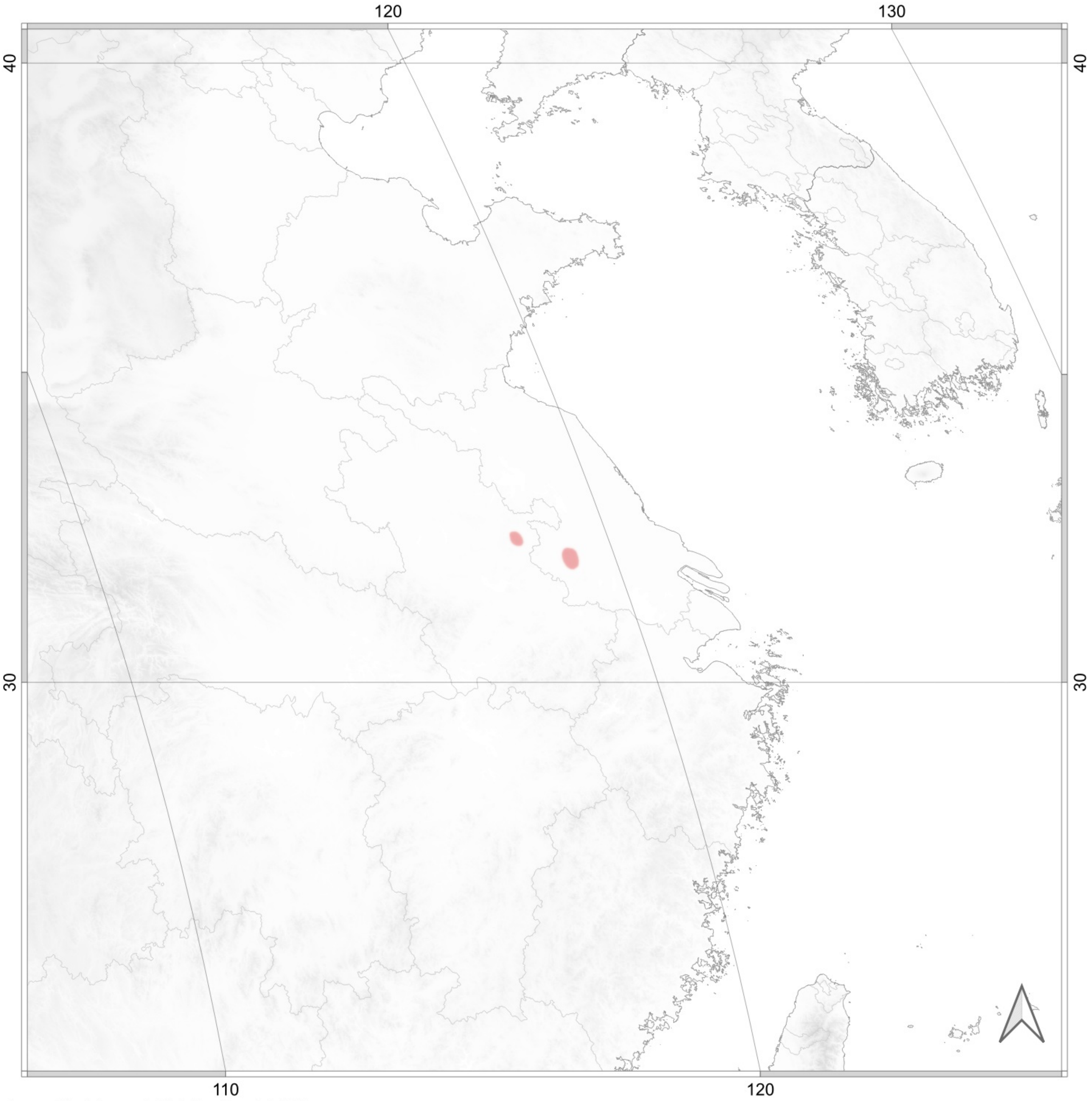

Source of the data : see details in Fragnière et al., 2021

Ulmus crassifolia

Nutt.

Trans. Amer. Philos. Soc. ser. 2, 5: 169 (1835)

ULMACEAE

IUCN Red list status : LC
*
habitat-ecology : stream banks, low woods, low hillsides, roadsides, waste places
*
climate - Köppen classification : Cfa, BSh
*
indicative altitudinal range : 0 - 500 m
*
min. latitude : 25.1, max. latitude : 35.8,
min. longitude : -100.1, max. longitude : -82.8

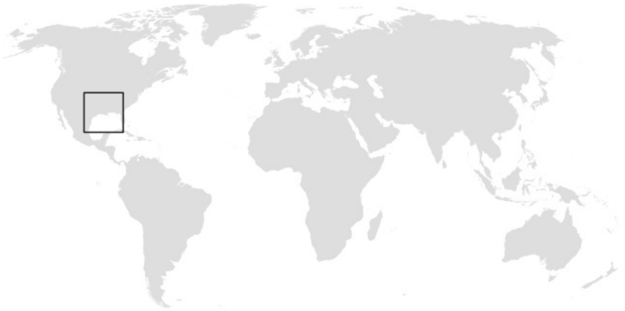

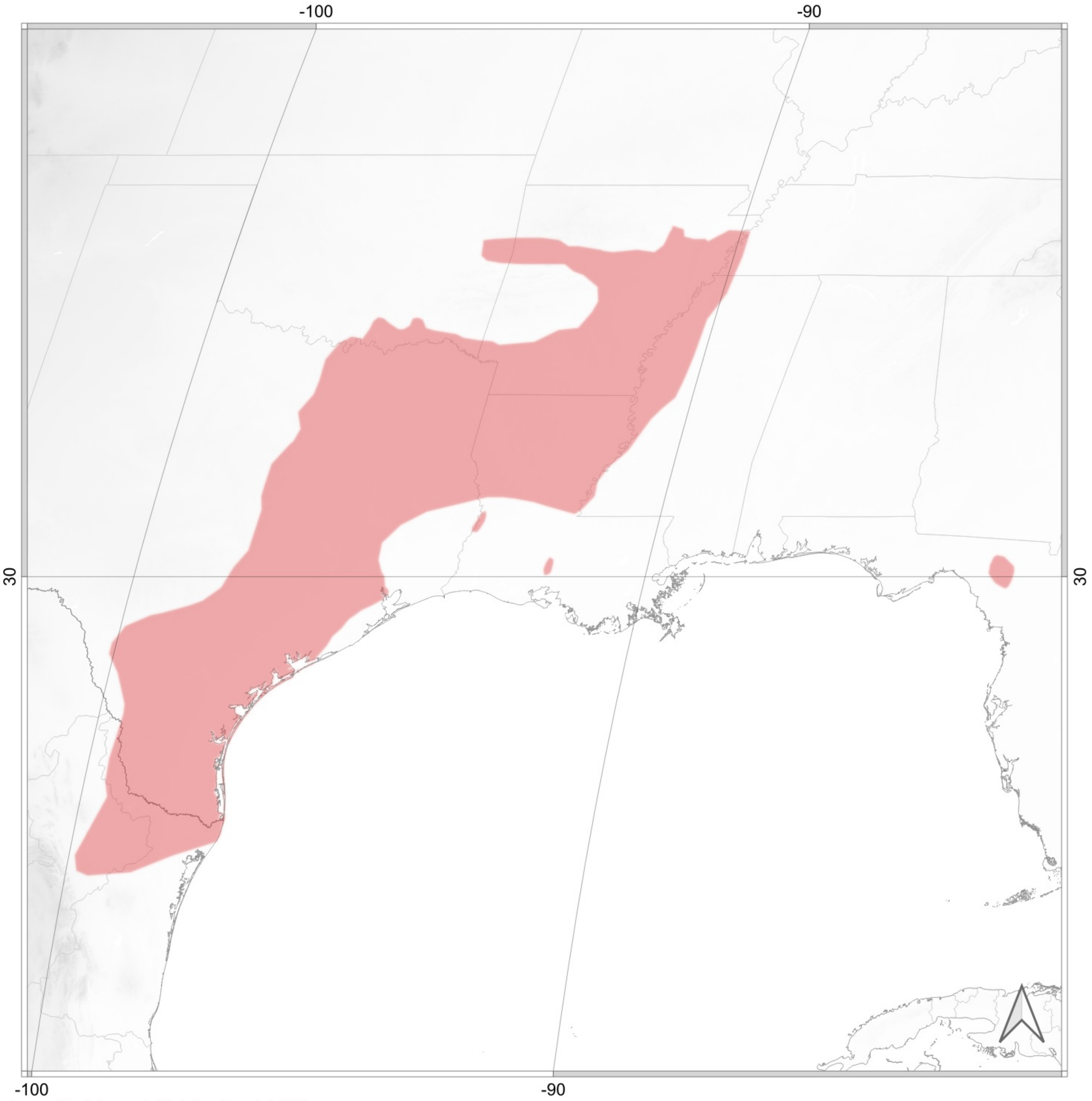

Source of the data : see details in Fragnière et al., 2021

Ulmus davidiana

Planch.

Prodr. 17: 158 (1873)

ULMACEAE

IUCN Red list status : LC

*

habitat-ecology : slopes, wetlands near streams, valleys

*

climate - Köppen classification : Dwa, Dwb, Dwc, Cwa, Cfa, Dfa, Dfb, (BSk)

*

indicative altitudinal range : 0 - 2300 m

*

min. latitude : 28.6, max. latitude : 54.8,
min. longitude : 104.7, max. longitude : 145.8

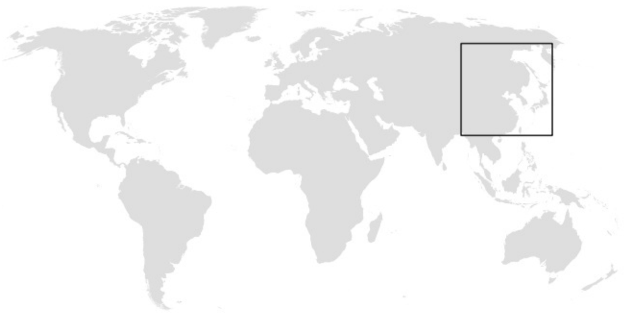

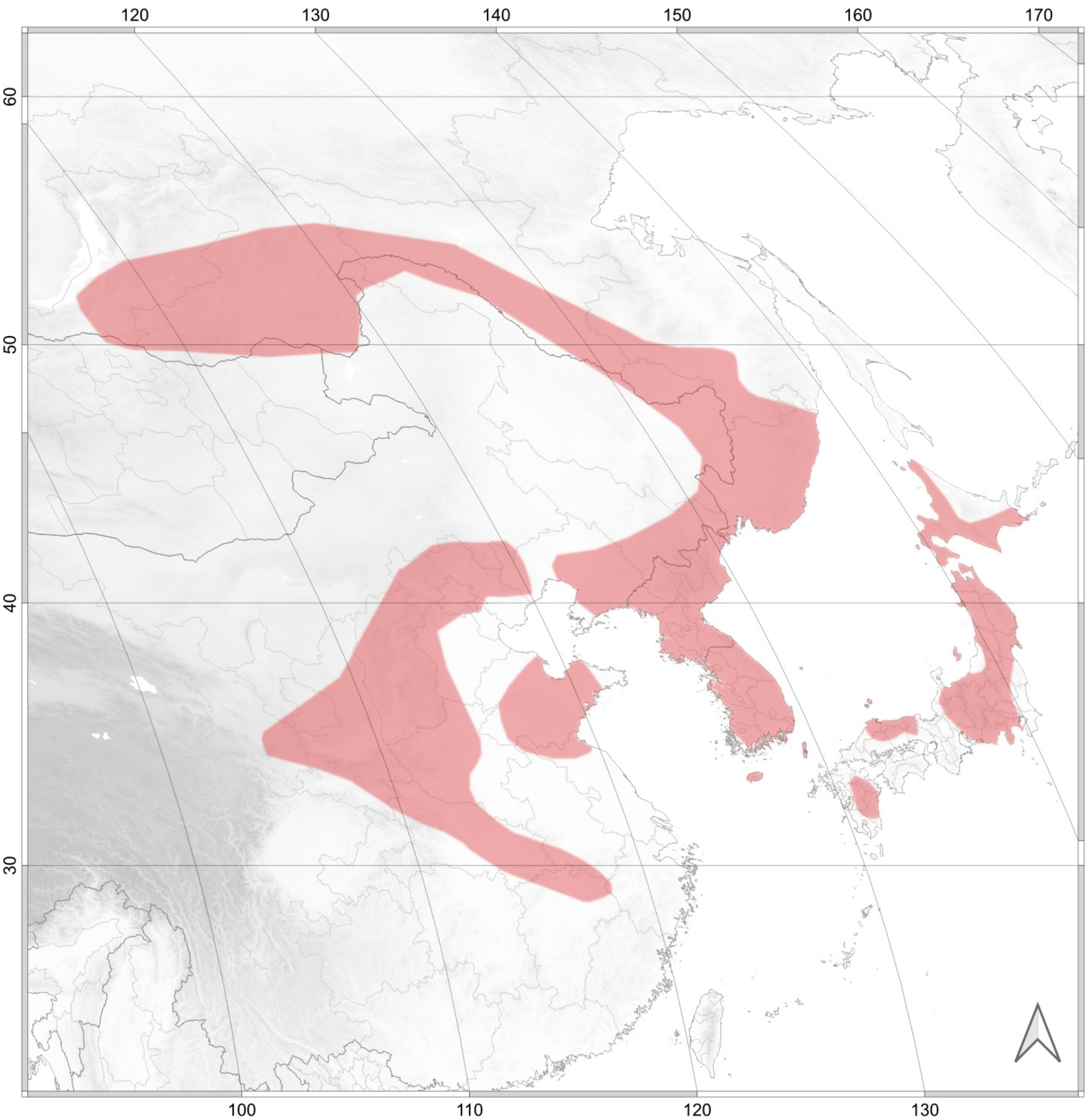

Source of the data : see details in Fragnière et al., 2021

Ulmus elongata

ULMACEAE

L.K.Fu & C.S.Ding

Acta Phytotax. Sin. 17(1): 46 (1979)

IUCN Red list status : VU
*
habitat-ecology : evergreen broad-leaved forests
*
climate - Köppen classification : Cfa
*
indicative altitudinal range : 200 - 900 m
*
min. latitude : 26.4, max. latitude : 29.8,
min. longitude : 113.8, max. longitude : 119.6

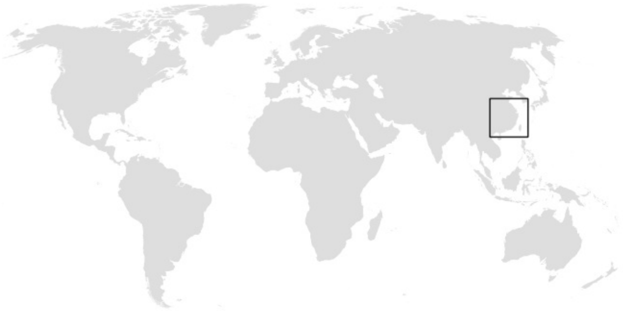

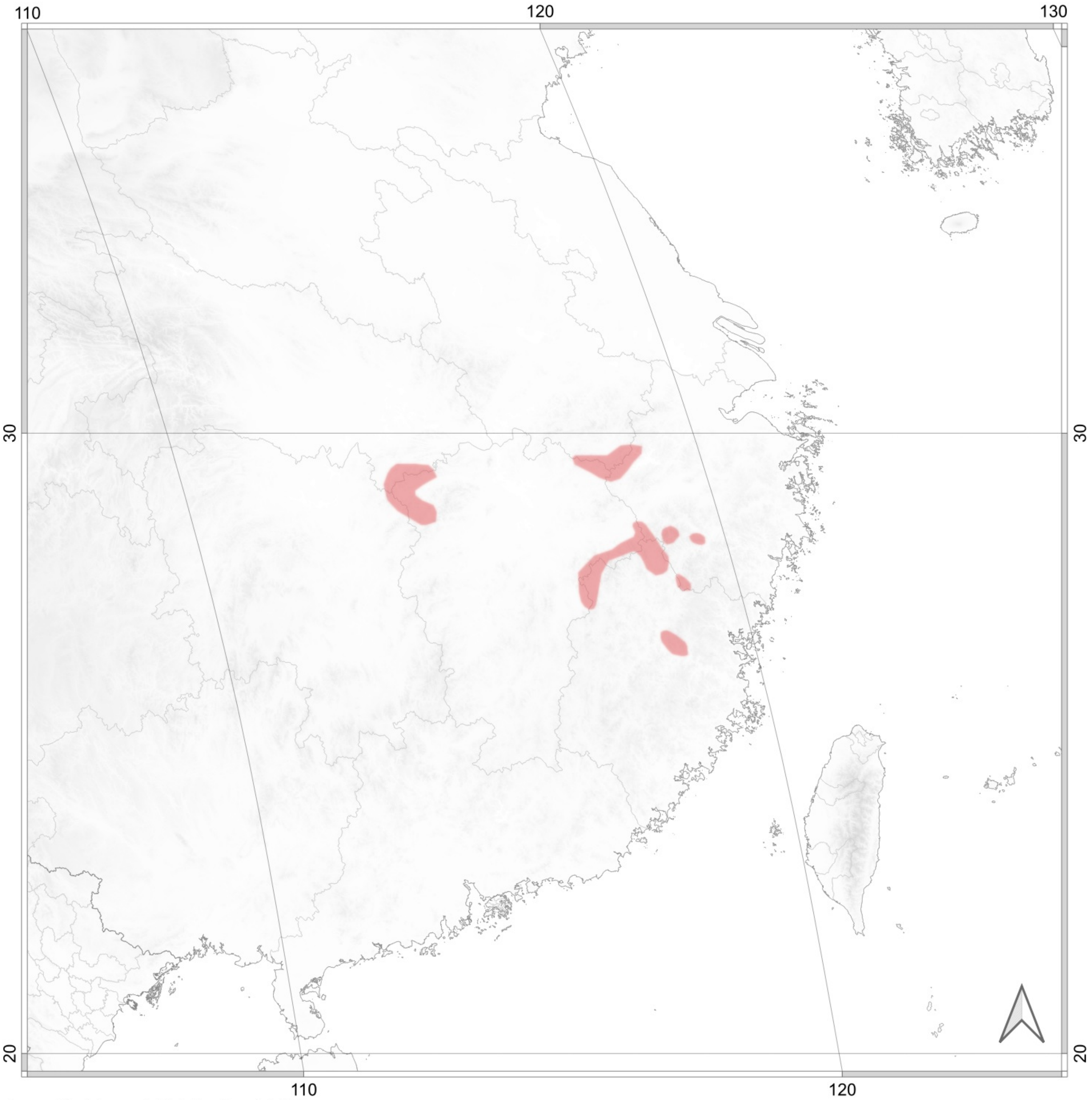

Source of the data : see details in Fragnière et al., 2021

Ulmus gaussenii

ULMACEAE

W.C. Cheng

Trav. Lab. Forest. Toulouse 3(3): 110, f. 1 (1939)

IUCN Red list status : CR
*
habitat-ecology : river banks, limestone mountains
*
climate - Köppen classification : Cfa
*
indicative altitudinal range : 0 - 300 m
*
min. latitude : 32.2, max. latitude : 32.3,
min. longitude : 118.2, max. longitude : 118.3

min+ max elevation estimated from distribution

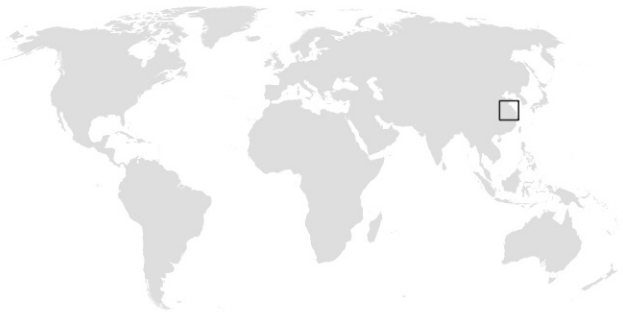

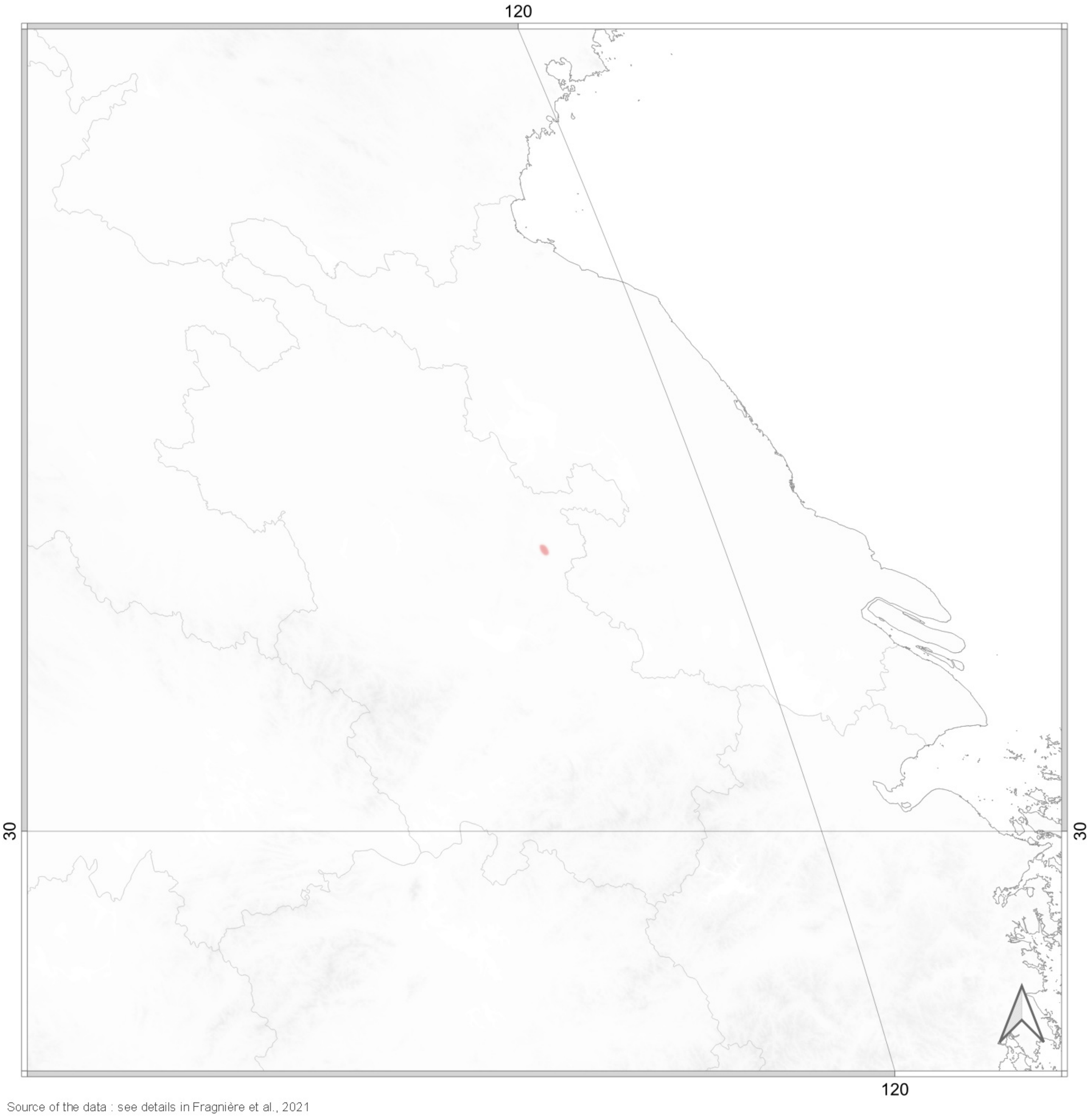

Source of the data : see details in Fragnière et al., 2021

Ulmus glabra

Huds.

Fl. Angl. (Hudson) 95 (1762)

ULMACEAE

IUCN Red list status : LC

*

habitat-ecology : temperate forests with cool summers, moist forests with rich soils and high humidity

*

climate - Köppen classification : Dfb, Cfb, (Dfc), (Dfa), (Csb)

*

indicative altitudinal range : 0 - 1500 m

*

min. latitude : 35.8, max. latitude : 68.1,
min. longitude : -10.5, max. longitude : 60.7

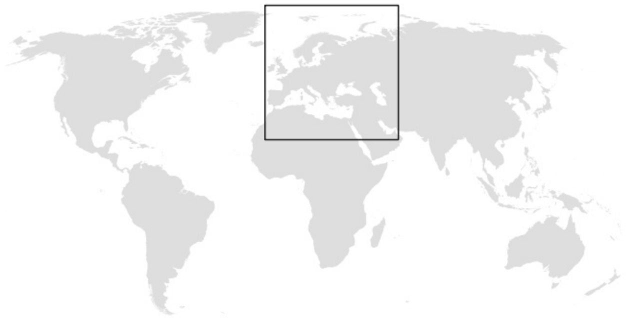

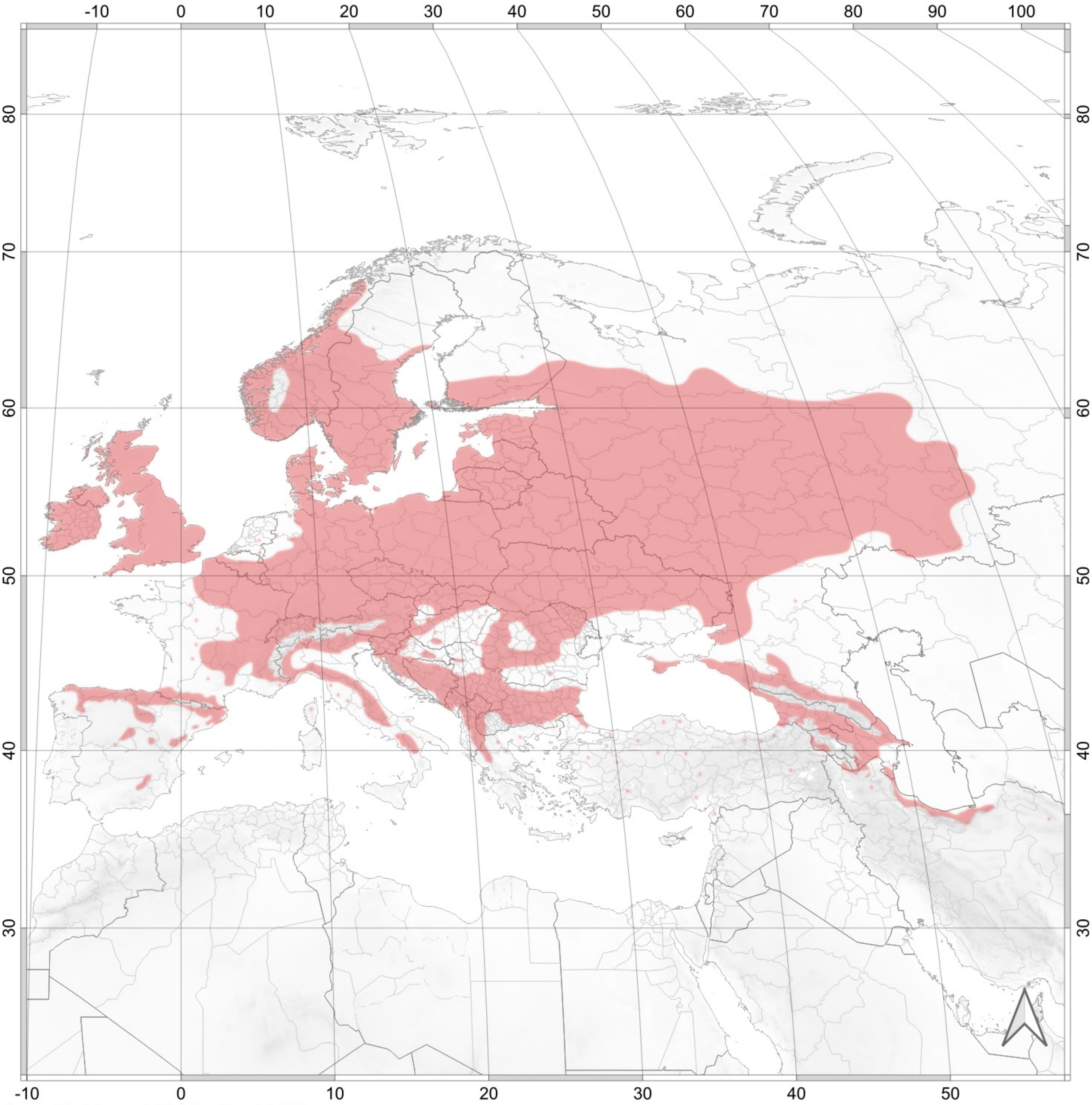

Source of the data : see details in Fragnière et al., 2021

Ulmus glaucescens

Franch.

Nouv. Arch. Mus. Hist. Nat., sér. 2, 7: 76–77, pl. 6, f. A (1884)

ULMACEAE

IUCN Red list status : ne

*

habitat-ecology : along rivers, mountain slopes, xeromorphic shrub and open forest

*

climate - Köppen classification : BSh, BSk, Dwa, Dwb

*

indicative altitudinal range : 500 - 2500 m

*

min. latitude : 33.3, max. latitude : 42.8,
min. longitude : 91.1, max. longitude : 120.7

min+ max elevation estimated from distribution

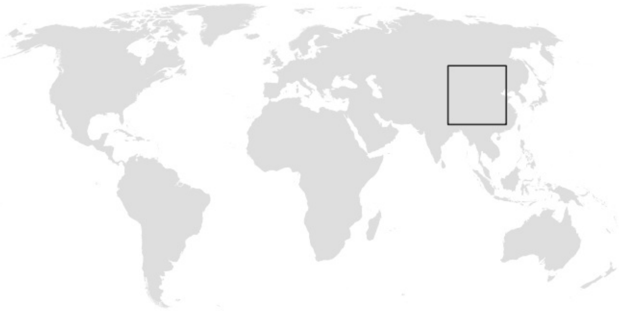

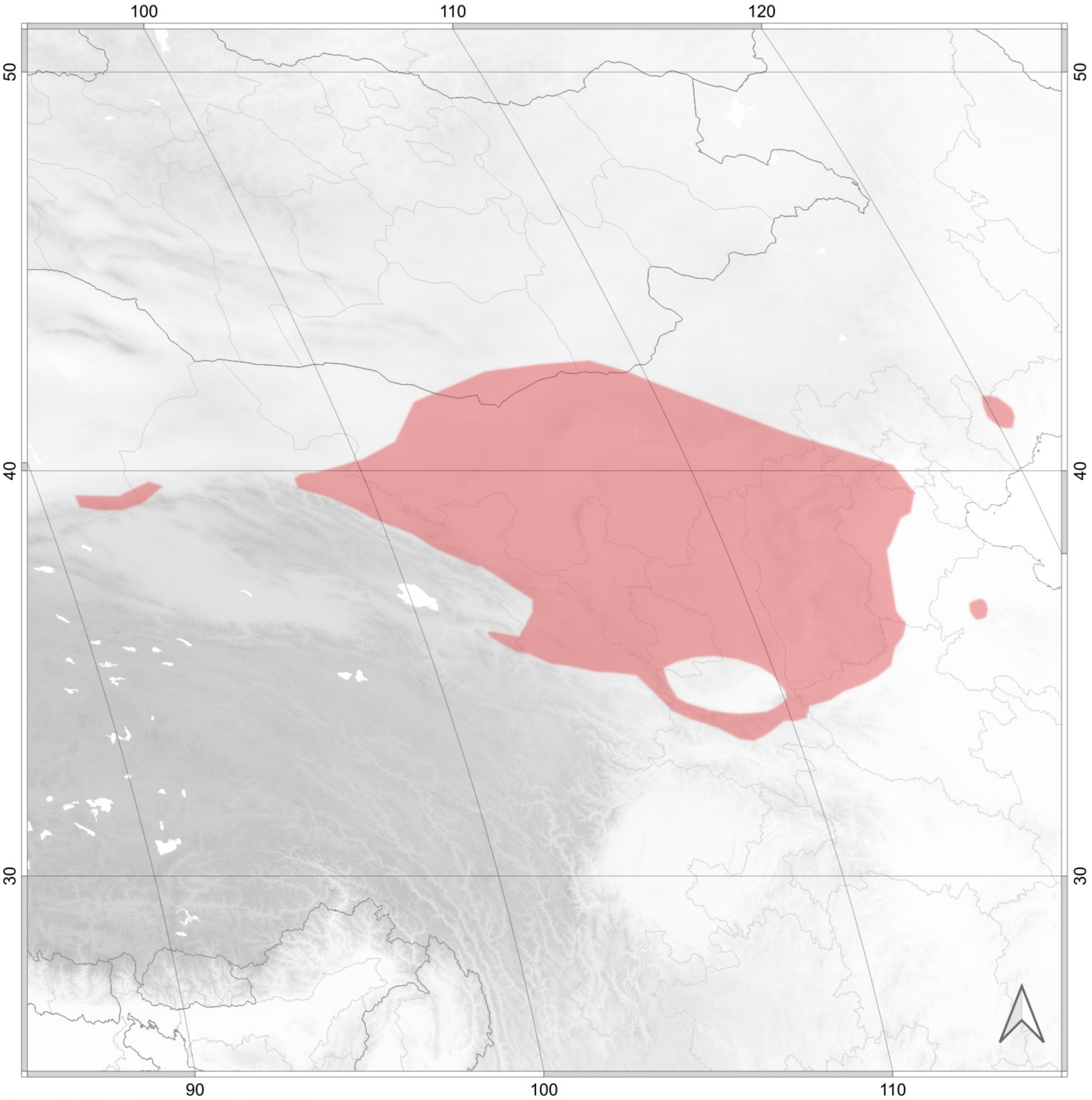

Source of the data : see details in Fragnière et al., 2021

Ulmus harbinensis

S.Q.Nie & G.Q.Huang

Bull. Bot. Res., Harbin 7(1): 151 (1987)

ULMACEAE

IUCN Red list status : ne
*
habitat-ecology : mixed woods
*
climate - Köppen classification : Dwa
*
indicative altitudinal range : 100 - 500 m
*
min. latitude : 45.1, max. latitude : 46.8,
min. longitude : 126, max. longitude : 127.9

poorly understood species should be reinvestigated. min+ max elevation estimated from distribution

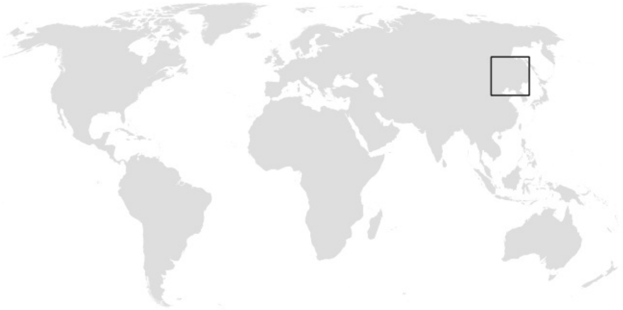

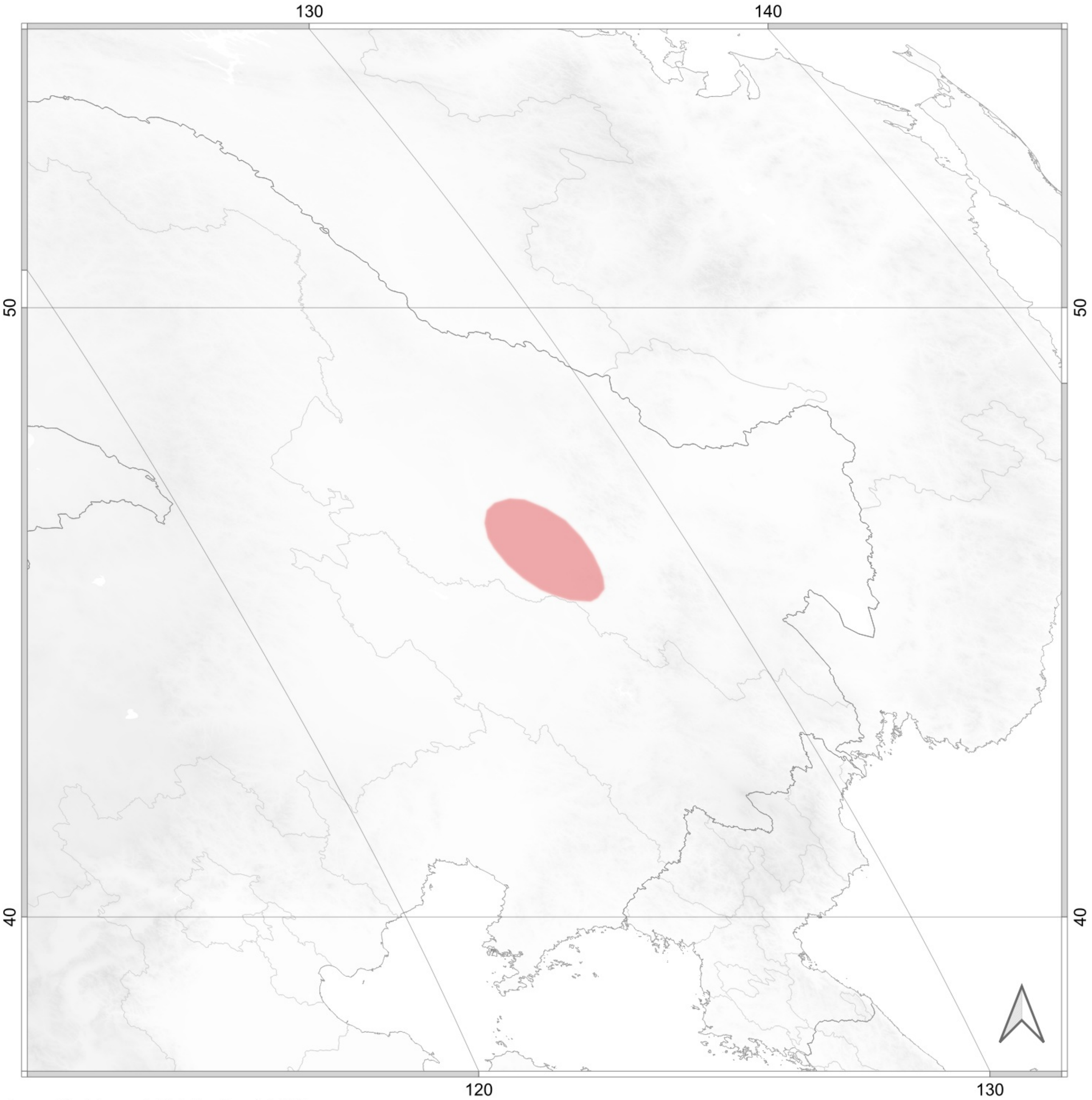

Source of the data : see details in Fragnière et al., 2021

Ulmus ismaelis

ULMACEAE

Todzia & Panero

Brittonia 50(3): 343 (1998)

IUCN Red list status : ne
*
habitat-ecology : riparian forests, canyons, near rivers
*
climate - Köppen classification : Aw, Cwb
*
indicative altitudinal range : 300 - 1500 m
*
min. latitude : 13.8, max. latitude : 17.5,
min. longitude : -98, max. longitude : -86

min+ max elevation estimated from distribution

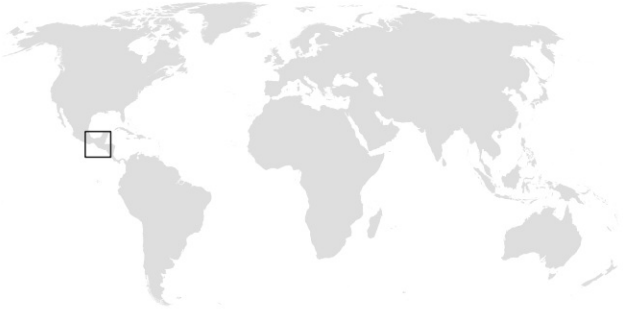

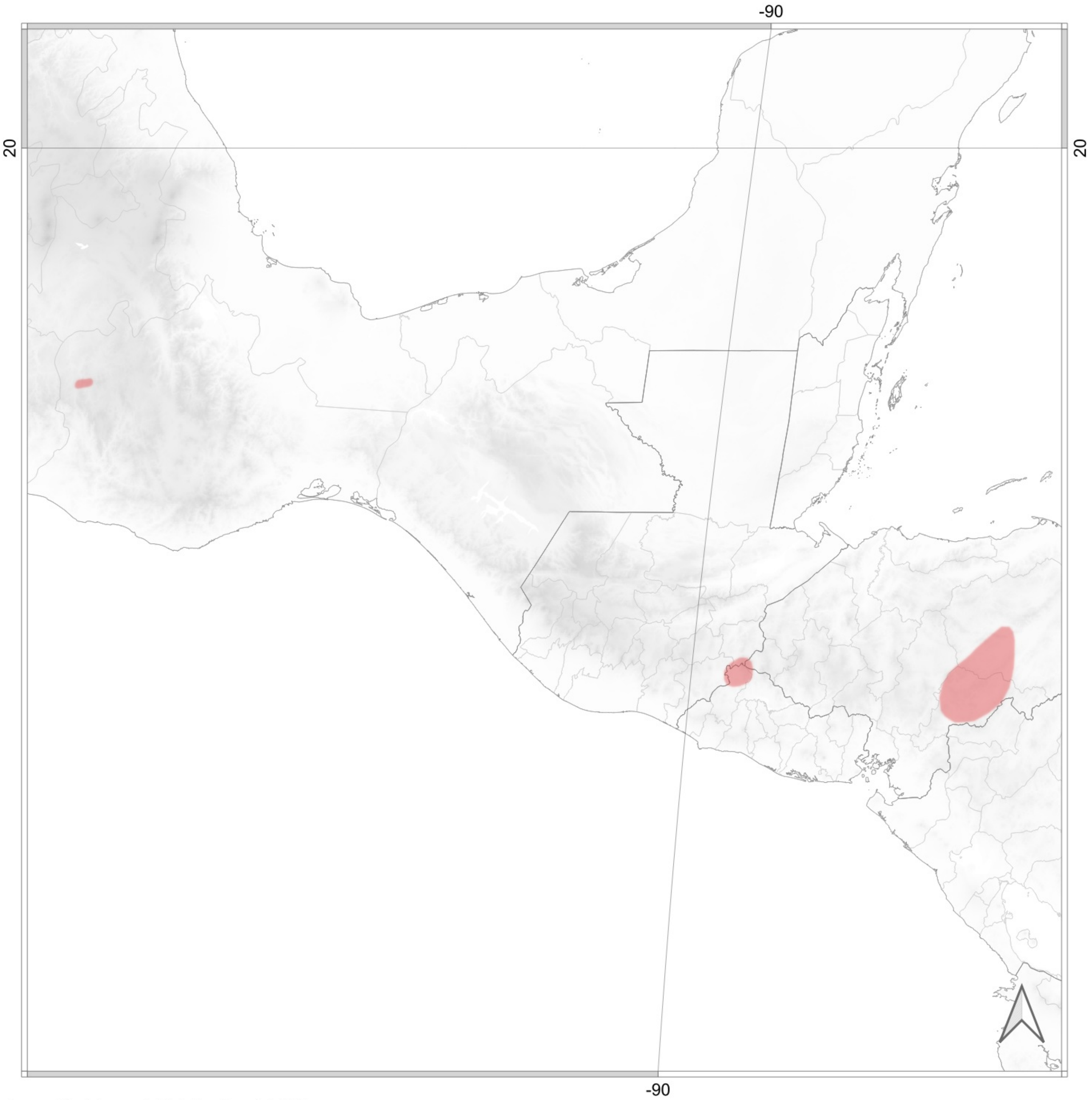

Source of the data : see details in Fragnière et al., 2021

Ulmus laciniata

Mayr

Fremdländ. Wald- Parkbäume 523 (1906)

ULMACEAE

IUCN Red list status : LC
*
habitat-ecology : temperate forests
*
climate - Köppen classification : Dwa, Dwb, Dfa, Dfb, (Dwc), (BSk), (Dfc)
*
indicative altitudinal range : 0 - 2200 m
*
min. latitude : 34.2, max. latitude : 48.4,
min. longitude : 109.7, max. longitude : 146.6

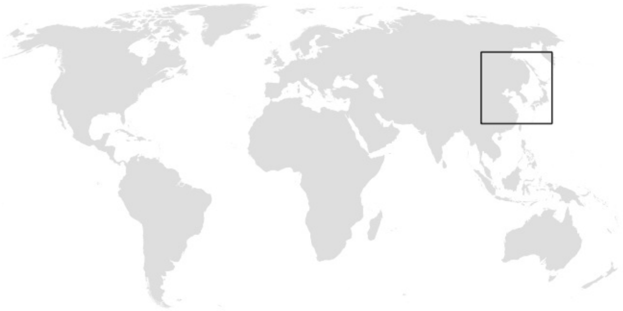

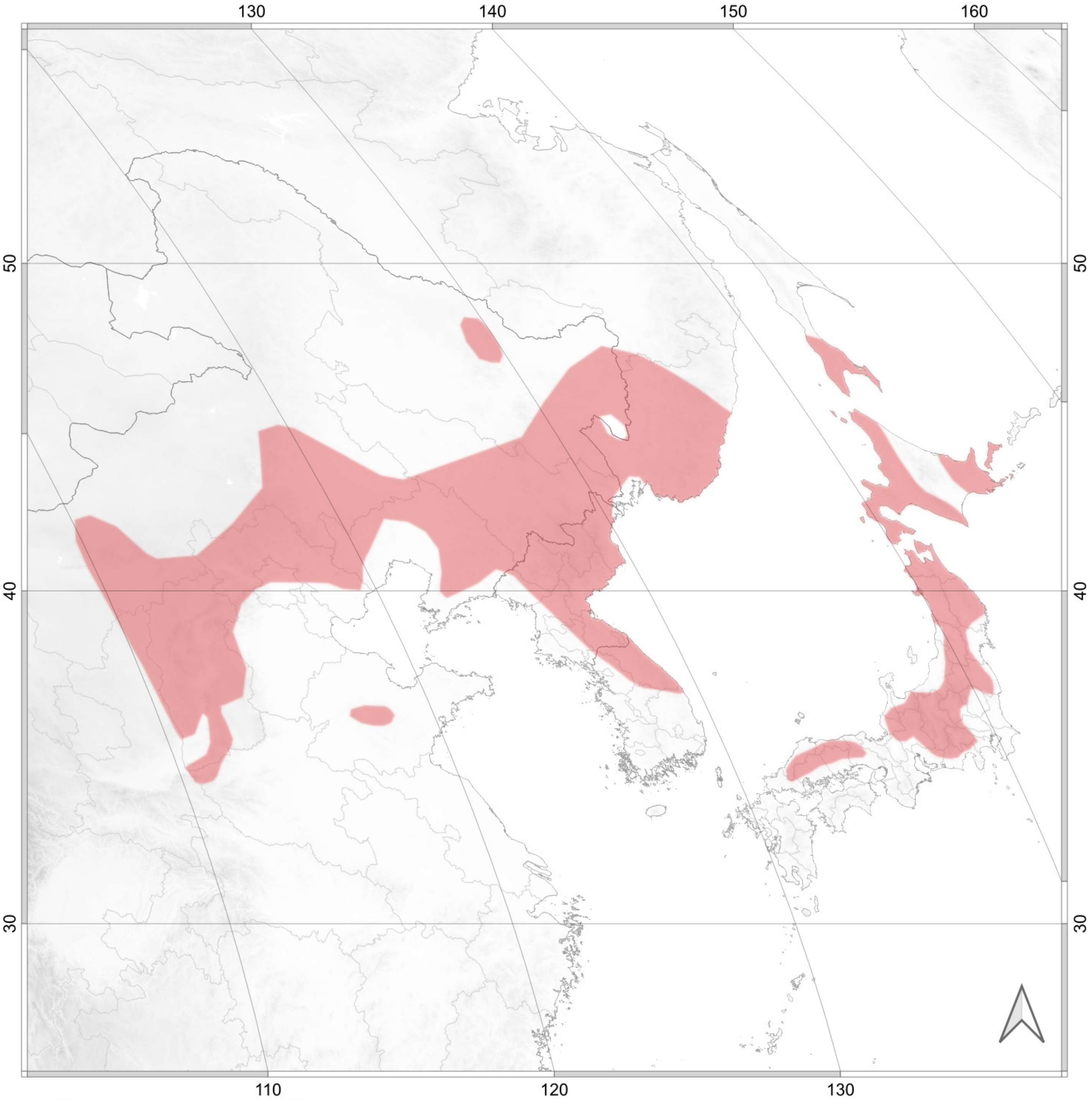

Source of the data : see details in Fragnière et al., 2021

Ulmus laevis

ULMACEAE

Pall.

Fl. Ross. 1(1): 75 (1784)

IUCN Red list status : DD

*

habitat-ecology : riparian forests, along large rivers, thriving in damp, periodically flooded soils

*

climate - Köppen classification : Dfb, Cfb, (Dfc), (BSk)

*

indicative altitudinal range : 0 - 1000 m

*

min. latitude : 36.7, max. latitude : 63.1,
min. longitude : -6.8, max. longitude : 63.6

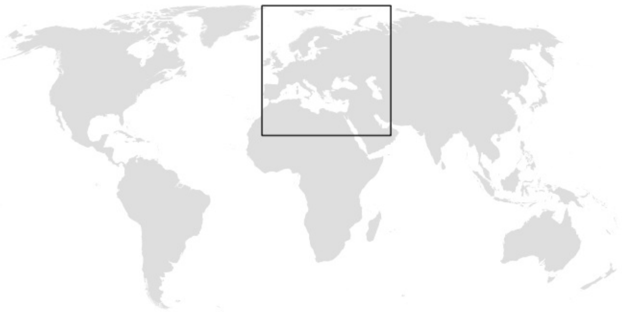

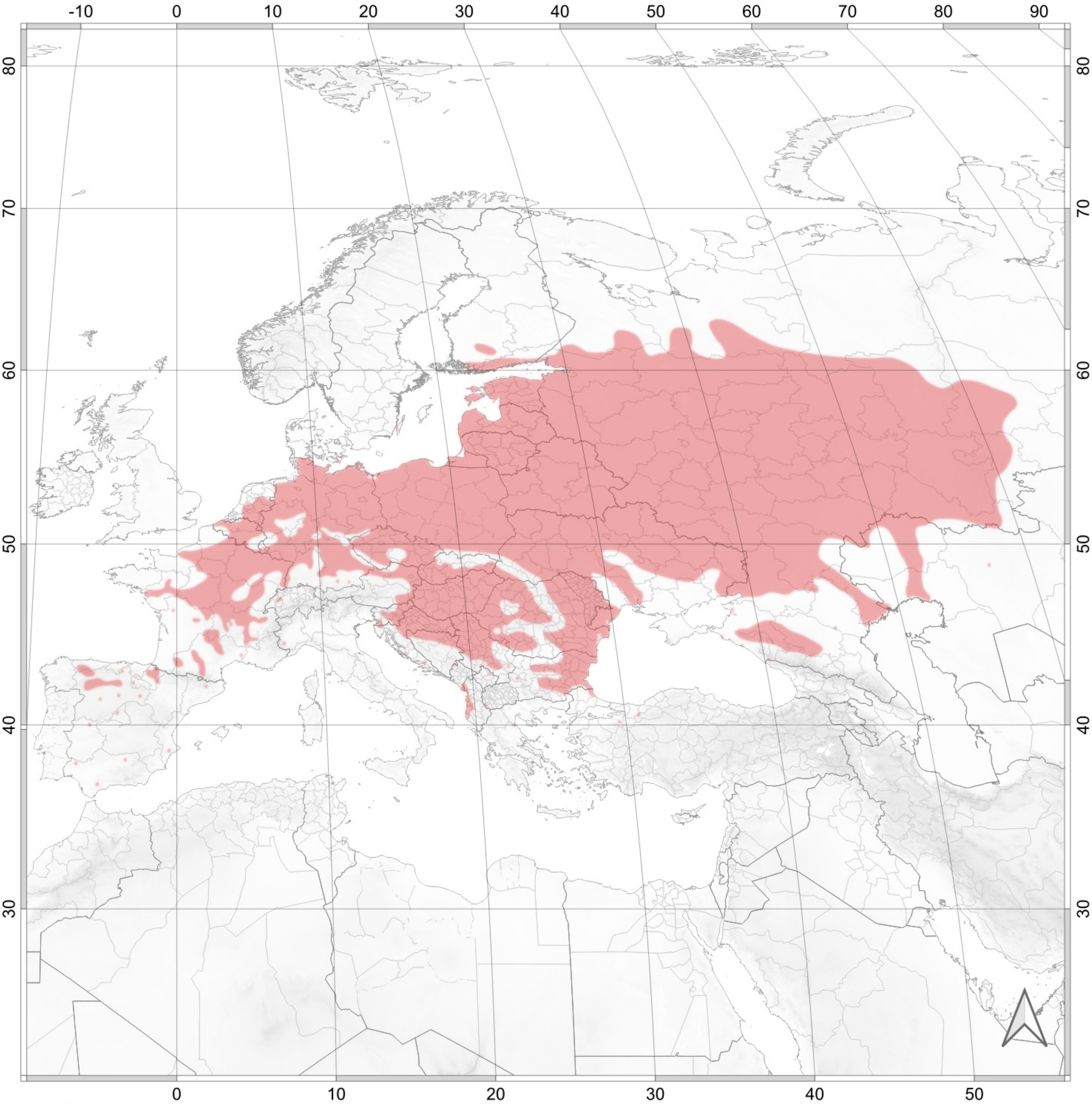

Source of the data : see details in Fragnière et al., 2021

Ulmus lamellosa

Z.Wang & S.L.Chang

Acta Phytotax. Sin. 17(1): 47 (1979)

ULMACEAE

IUCN Red list status : ne
*
habitat-ecology : mountain ravines
*
climate - Köppen classification : Dwa, Dwb, BSk
*
indicative altitudinal range : 500 - 1500 m
*
min. latitude : 34.2, max. latitude : 42,
min. longitude : 110.2, max. longitude : 117.7

min+ max elevation estimated from distribution

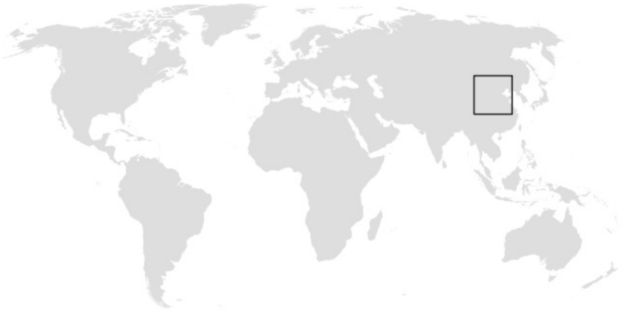

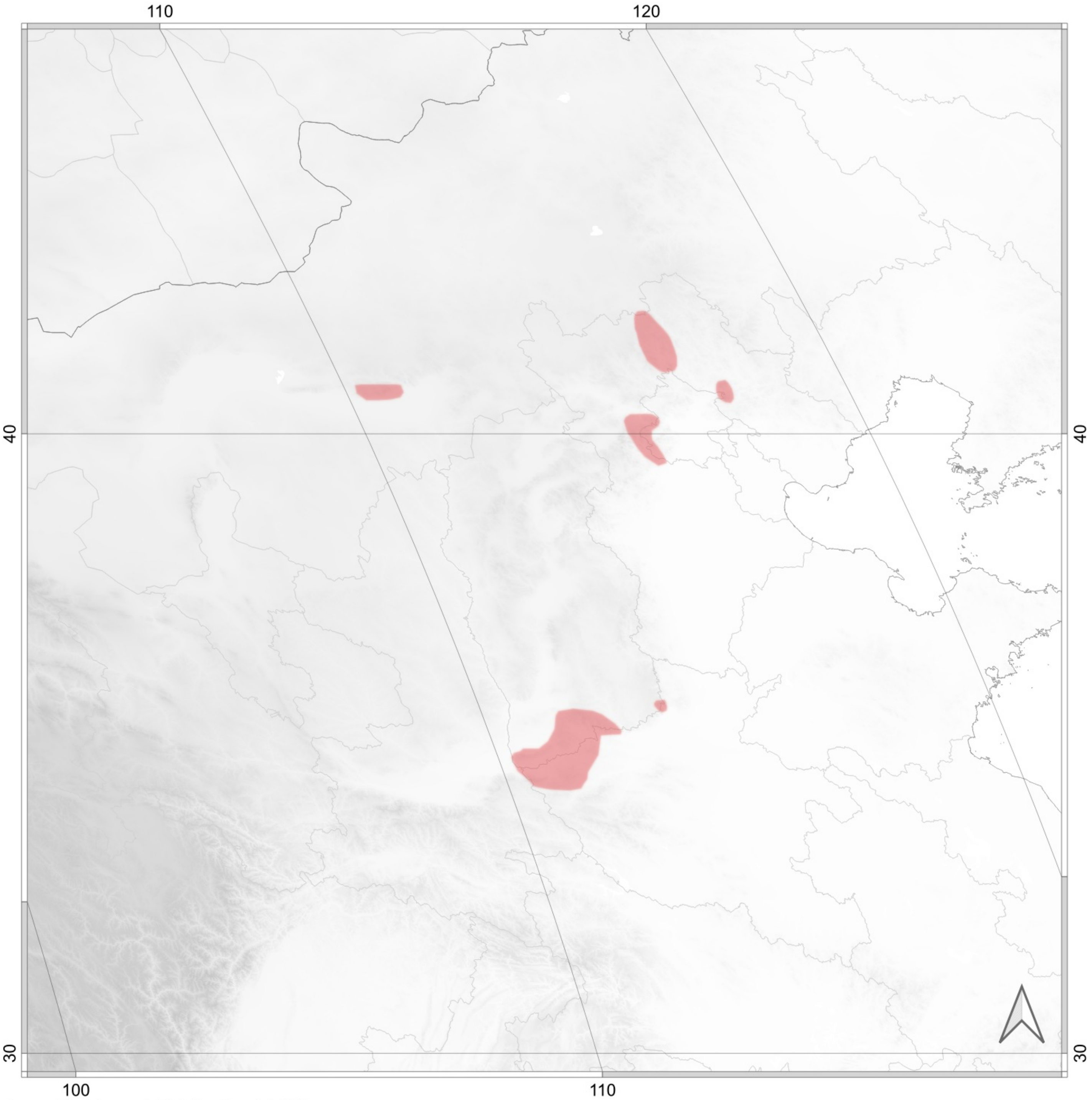

Source of the data : see details in Fragnière et al., 2021

Ulmus lanceifolia

ULMACEAE

Roxb. ex Wall.

Pl. Asiat. Rar. 2: 86, pl. 200 (1831)

IUCN Red list status : ne
*
habitat-ecology : no information
*
climate - Köppen classification : Cwa, Cwb, Aw, (Cfb), (Af), (Am)
*
indicative altitudinal range : 100 - 2000 m
*
min. latitude : -8.8, max. latitude : 28.7,
min. longitude : 88.2, max. longitude : 121

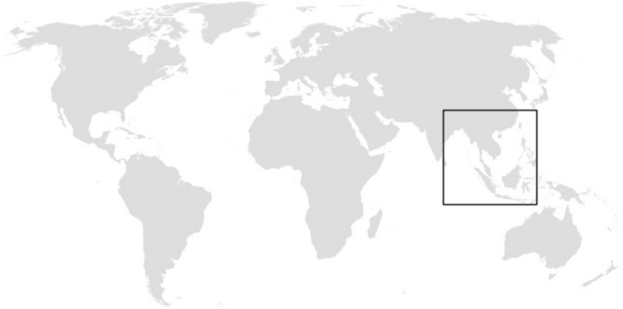

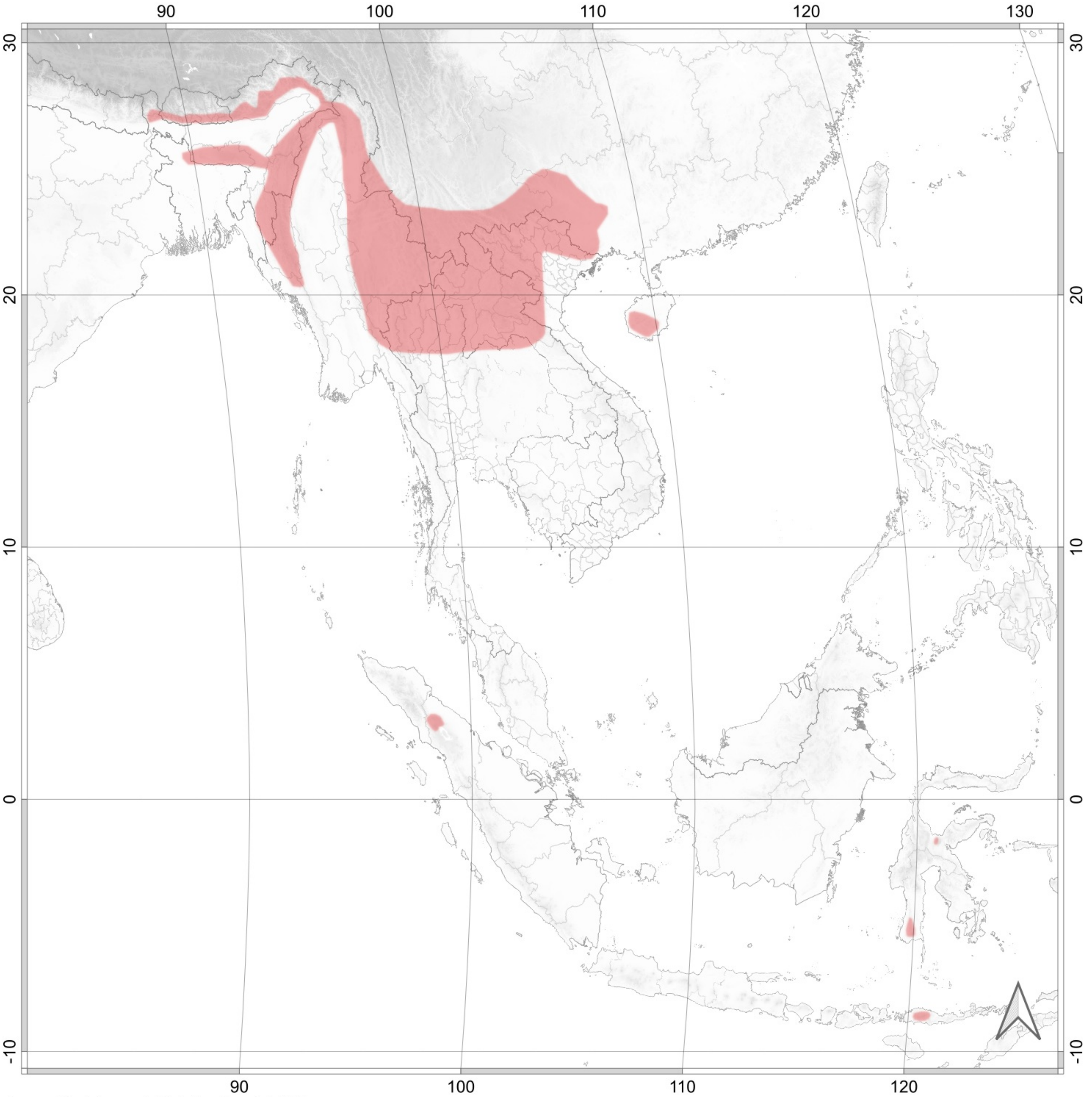

Source of the data : see details in Fragnière et al., 2021

Ulmus macrocarpa

ULMACEAE

Hance

J. Bot. 6: 332 (1868)

IUCN Red list status : LC
*
habitat-ecology : mixed forests, slopes, valleys
*
climate - Köppen classification : Dwa, Dwb, Dwc, BSk, (Cfa), (Cwa)
*
indicative altitudinal range : 0 - 1800 m
*
min. latitude : 27.2, max. latitude : 52.6,
min. longitude : 102.7, max. longitude : 136.3

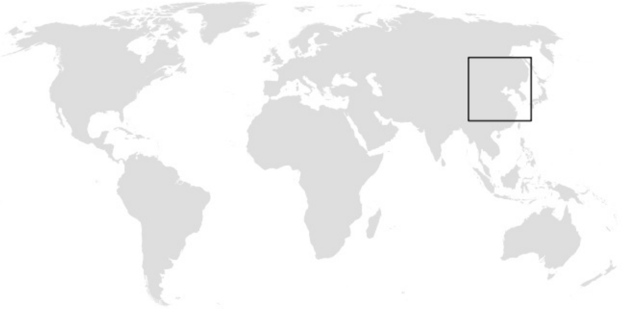

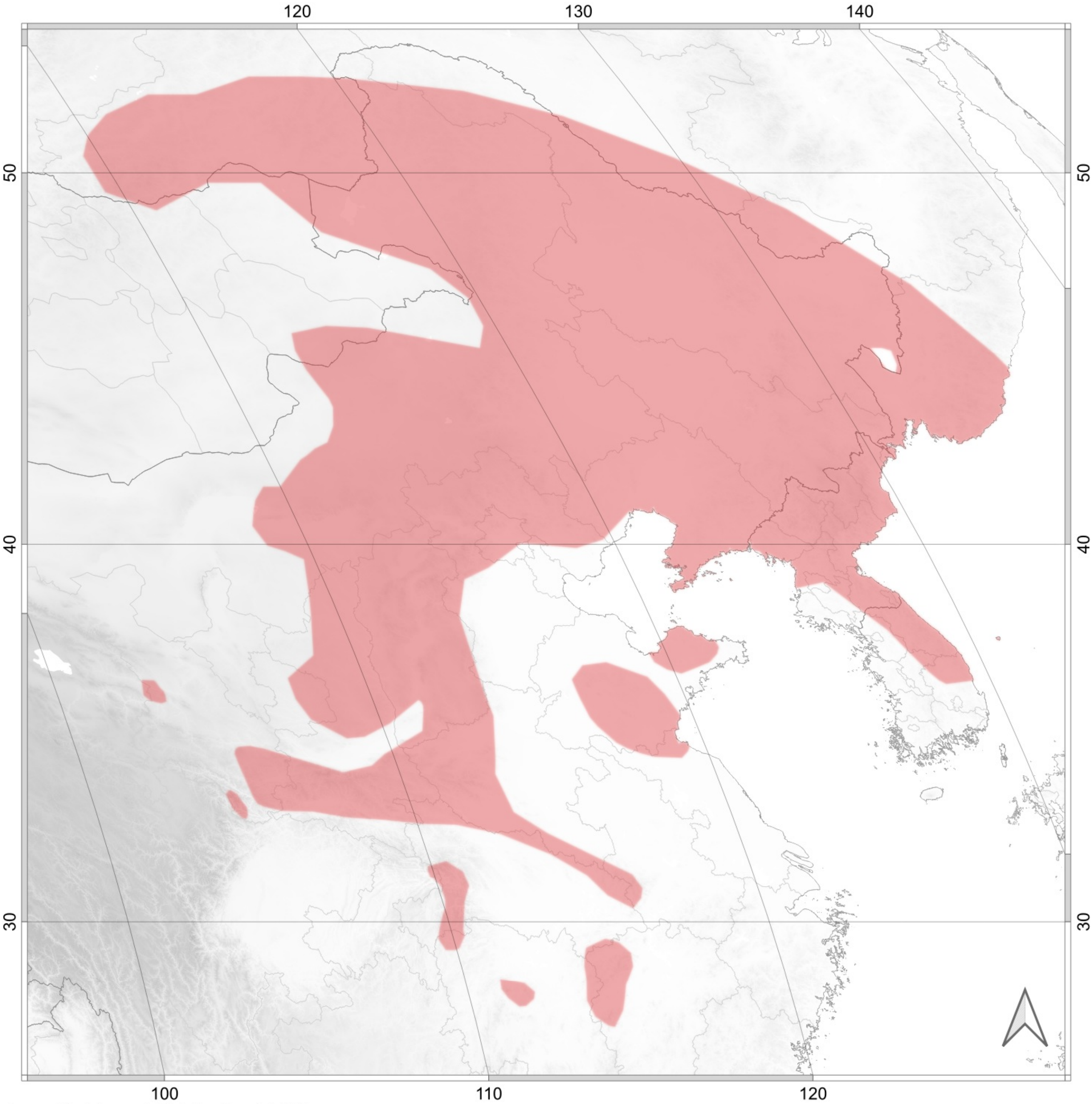

Source of the data : see details in Fragnière et al., 2021

Ulmus mexicana

Planch.

Prodr. [A. P. de Candolle] 17: 156 (1873)

ULMACEAE

IUCN Red list status : ne
*
habitat-ecology : wet forests and very wet mountains, volvanic, calcareous, and metamorphic soils
*
climate - Köppen classification : Aw, Am, Af, Cwb, Cfb, Cfa, Cwa
*
indicative altitudinal range : 800 - 2200 m
*
min. latitude : 8.6, max. latitude : 21.7,
min. longitude : -101, max. longitude : -81.8

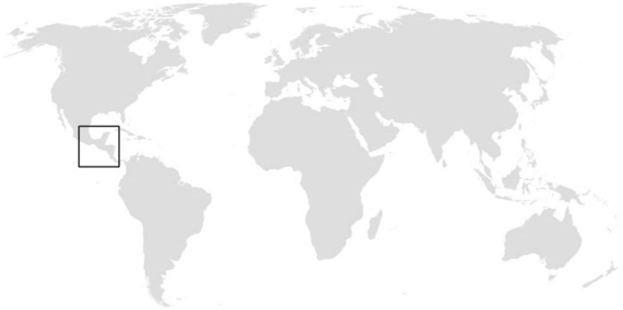

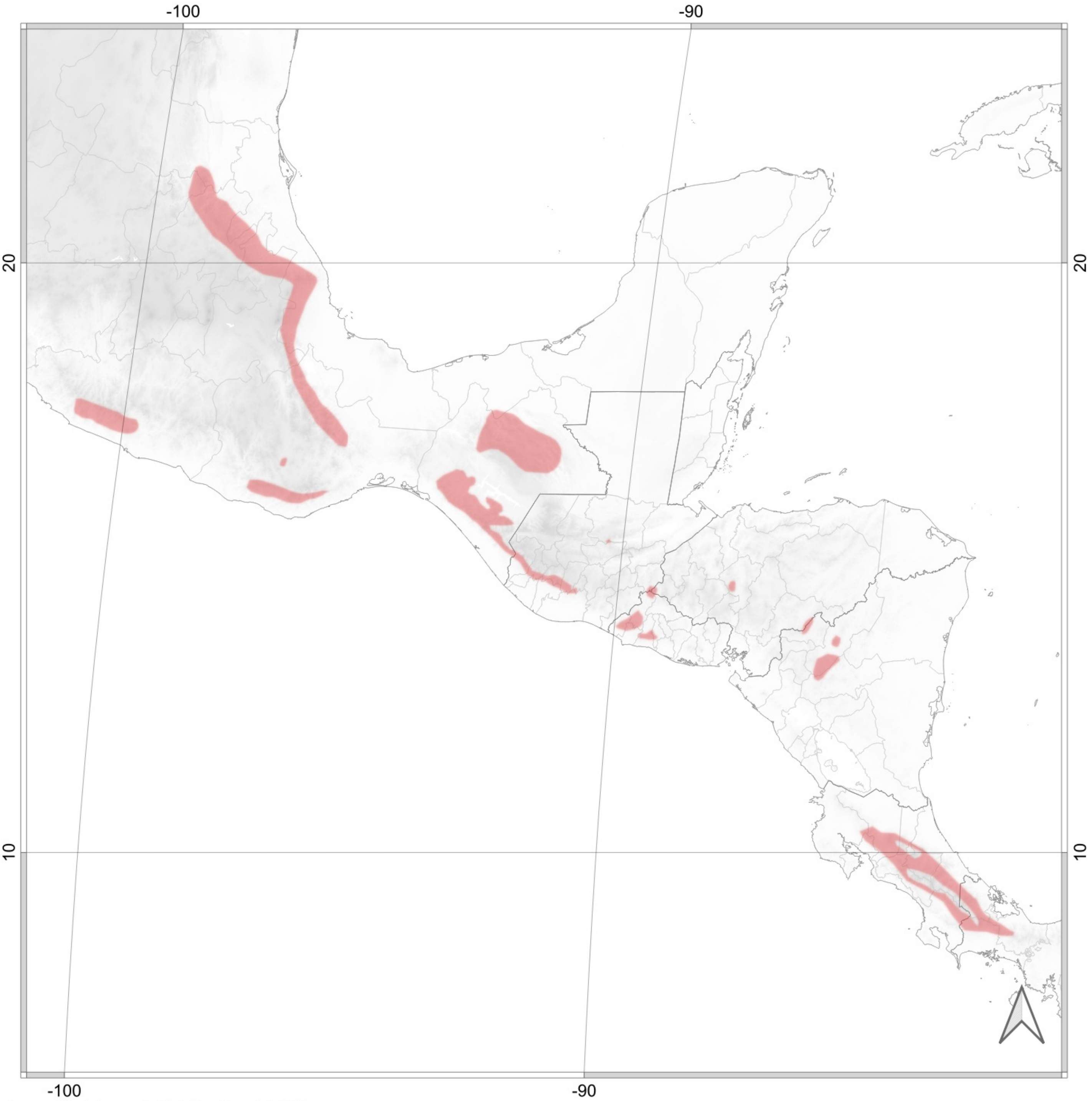

Source of the data : see details in Fragnière et al., 2021

Ulmus mianzhuensis

T.P. Yi & Lin Yang

Bull. Bot. Res., Harbin 26(6): 642–643 (2006)

ULMACEAE

IUCN Red list status : ne
*
habitat-ecology : no information
*
climate - Köppen classification : Cwa
*
indicative altitudinal range : 500 - 700 m
*
min. latitude : 31.2, max. latitude : 31.5,
min. longitude : 104.1, max. longitude : 104.4

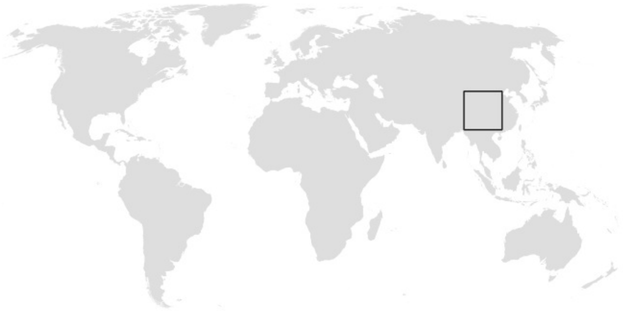

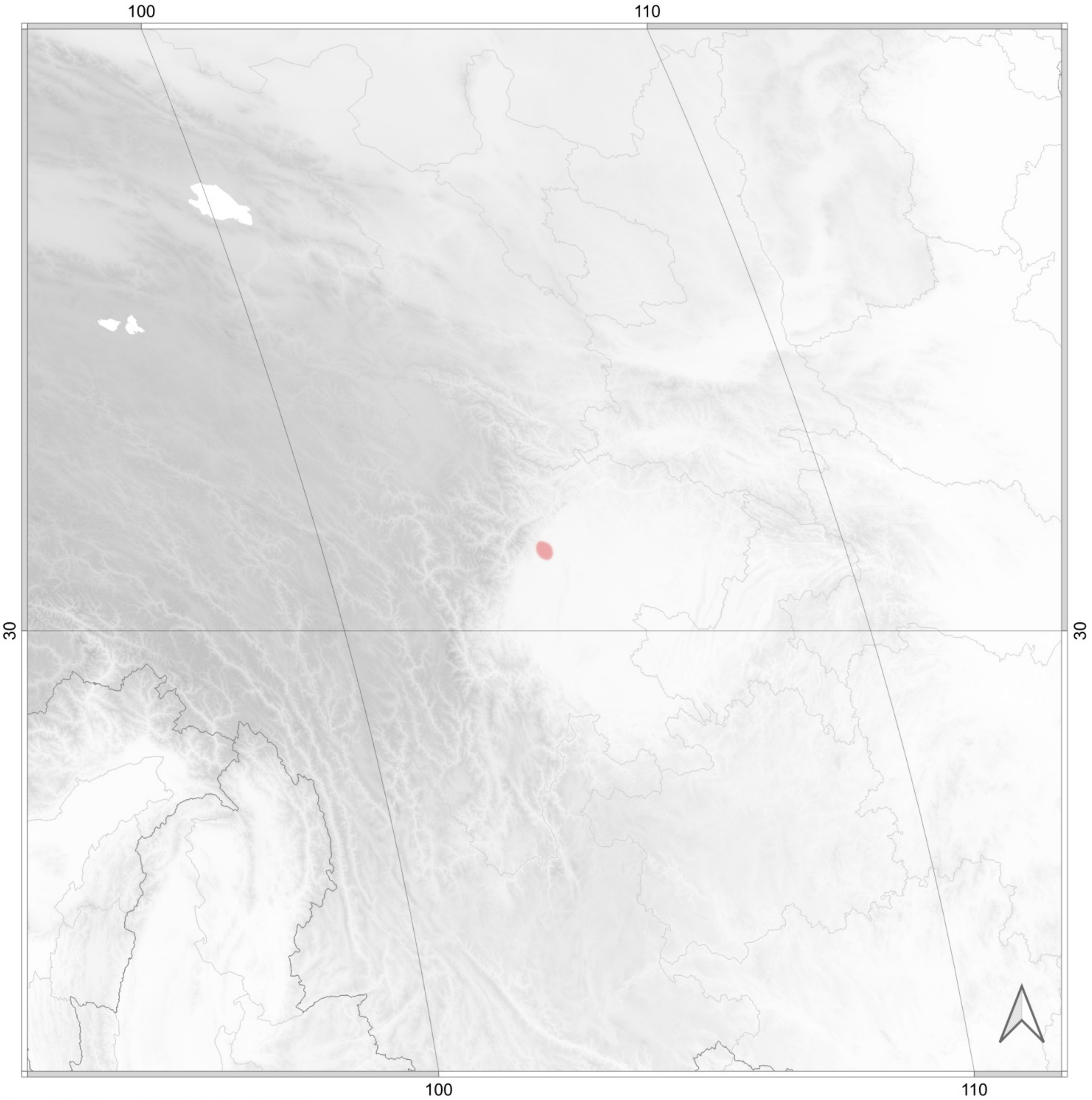

Source of the data : see details in Fragnière et al., 2021

Ulmus microcarpa

L.K. Fu

Acta Phytotax. Sin. 17(1): 48–49, pl. 2, f. 5–8 (1979)

ULMACEAE

IUCN Red list status : ne

*

habitat-ecology : broad-leaved forests

*

climate - Köppen classification : Dwb, Cwb

*

indicative altitudinal range : 2700 - 2900 m

*

min. latitude : 28.2, max. latitude : 29.3,
min. longitude : 96.6, max. longitude : 98.6

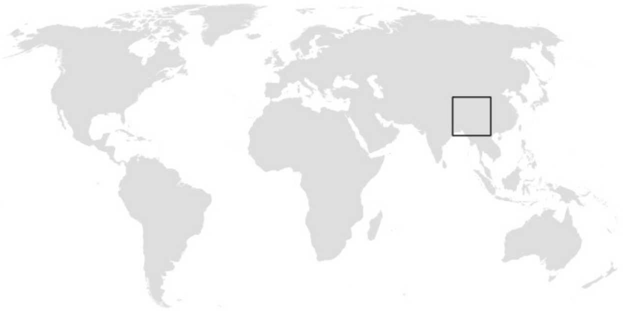

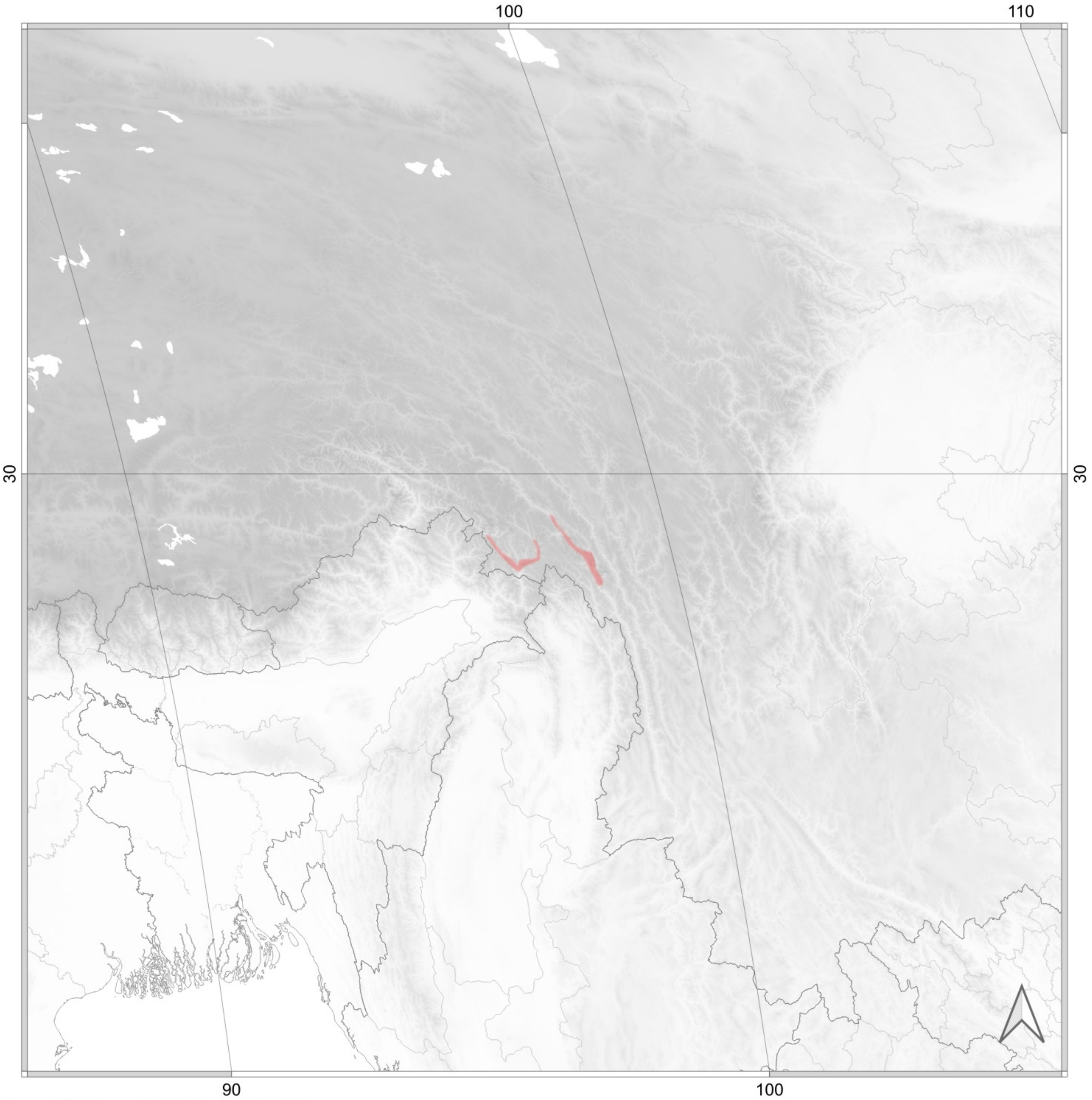

Source of the data : see details in Fragnière et al., 2021

Ulmus minor

Mill.

Gard. Dict., ed. 8. n. 6 (1768)

ULMACEAE

IUCN Red list status : DD

*

habitat-ecology : riparian habitats, pioneer species, characterised by being light-demanding, tolerate drought

*

climate - Köppen classification : Cfb, Dfb, BSk, Dfa, Cfa, Csa, (Csb), (Dfc)

*

indicative altitudinal range : 0 - 1300 m

*

min. latitude : 30, max. latitude : 58,
min. longitude : -9.5, max. longitude : 70.5

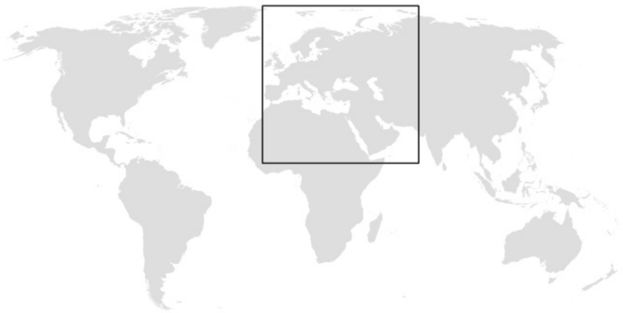

includes U. densa (synonym)

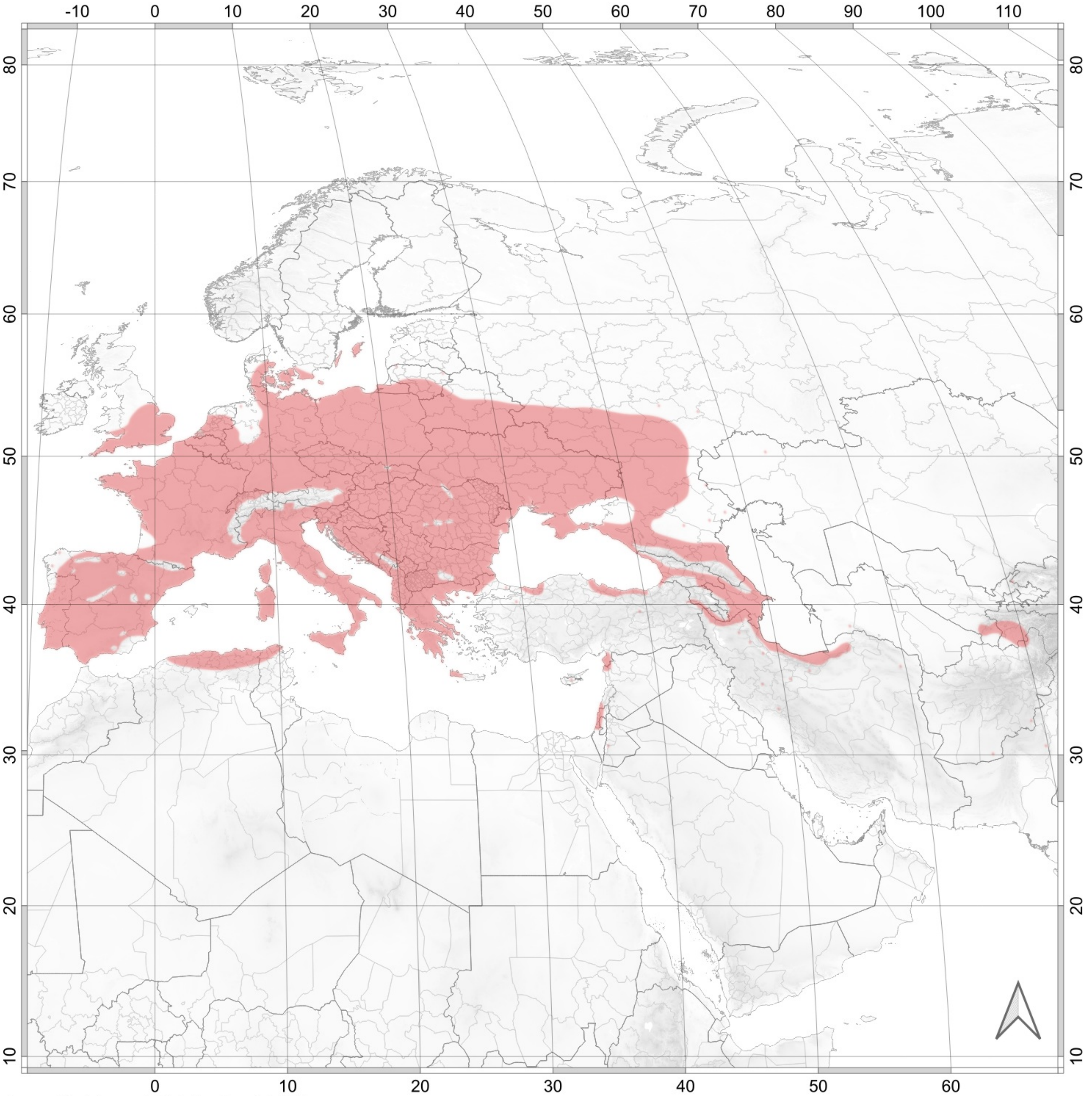

Source of the data : see details in Fragnière et al., 2021

Ulmus parvifolia

Jacq.

Pl. Rar. Hort. Schoenbr. 3: 6, t. 262 (1798)

ULMACEAE

IUCN Red list status : LC
*
habitat-ecology : drought tolerant, but tolerate moist sites
*
climate - Köppen classification : Cfa, Cwa, Dwa
*
indicative altitudinal range : 0 - 800 m
*
min. latitude : 21.9, max. latitude : 39.3,
min. longitude : 105.8, max. longitude : 140.9

widely planted, natural distribution difficult to know

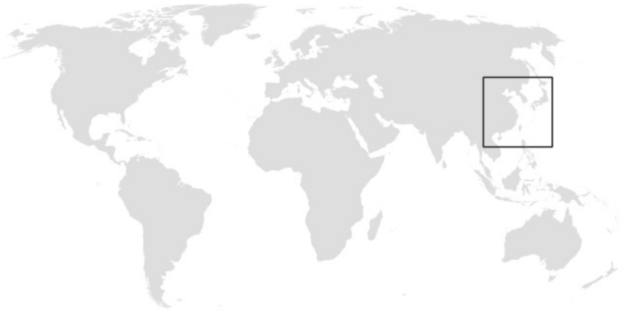

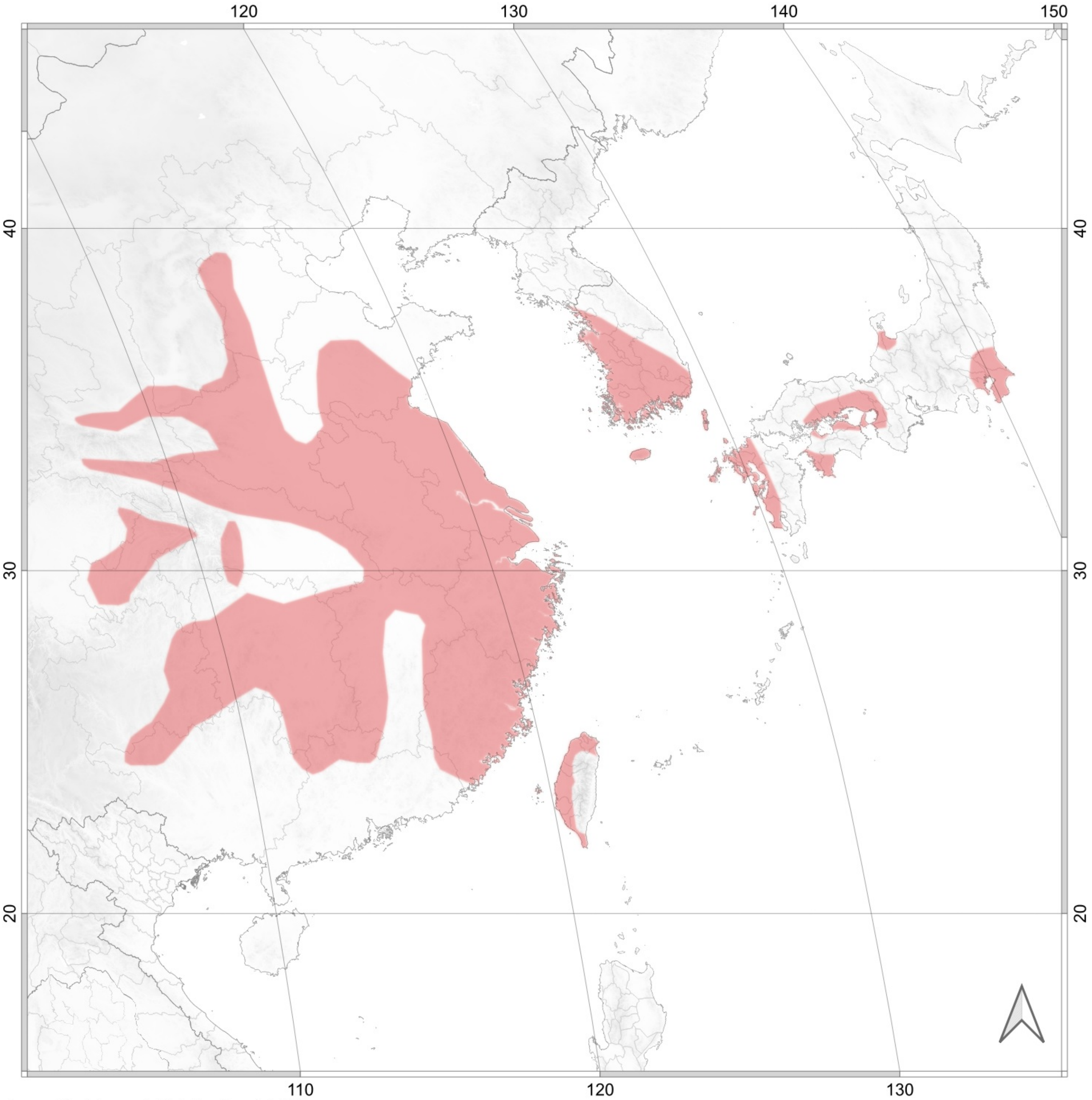

Source of the data : see details in Fragnière et al., 2021

Ulmus prunifolia

W.C.Cheng & L.K.Fu

Acta Phytotax. Sin. 17(1): 48 (1979)

ULMACEAE

IUCN Red list status : ne
*
habitat-ecology : no information
*
climate - Köppen classification : Cfa, Cwa
*
indicative altitudinal range : 1000 - 1500 m
*
min. latitude : 29.8, max. latitude : 31.4,
min. longitude : 107.7, max. longitude : 110.4

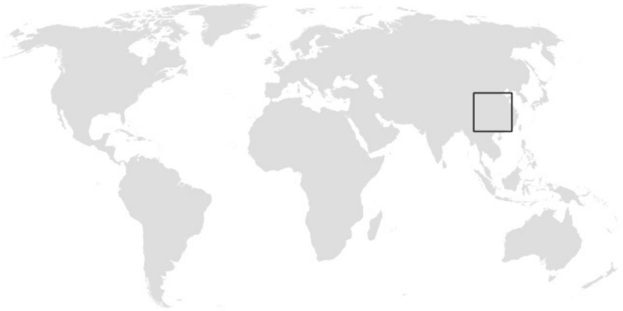

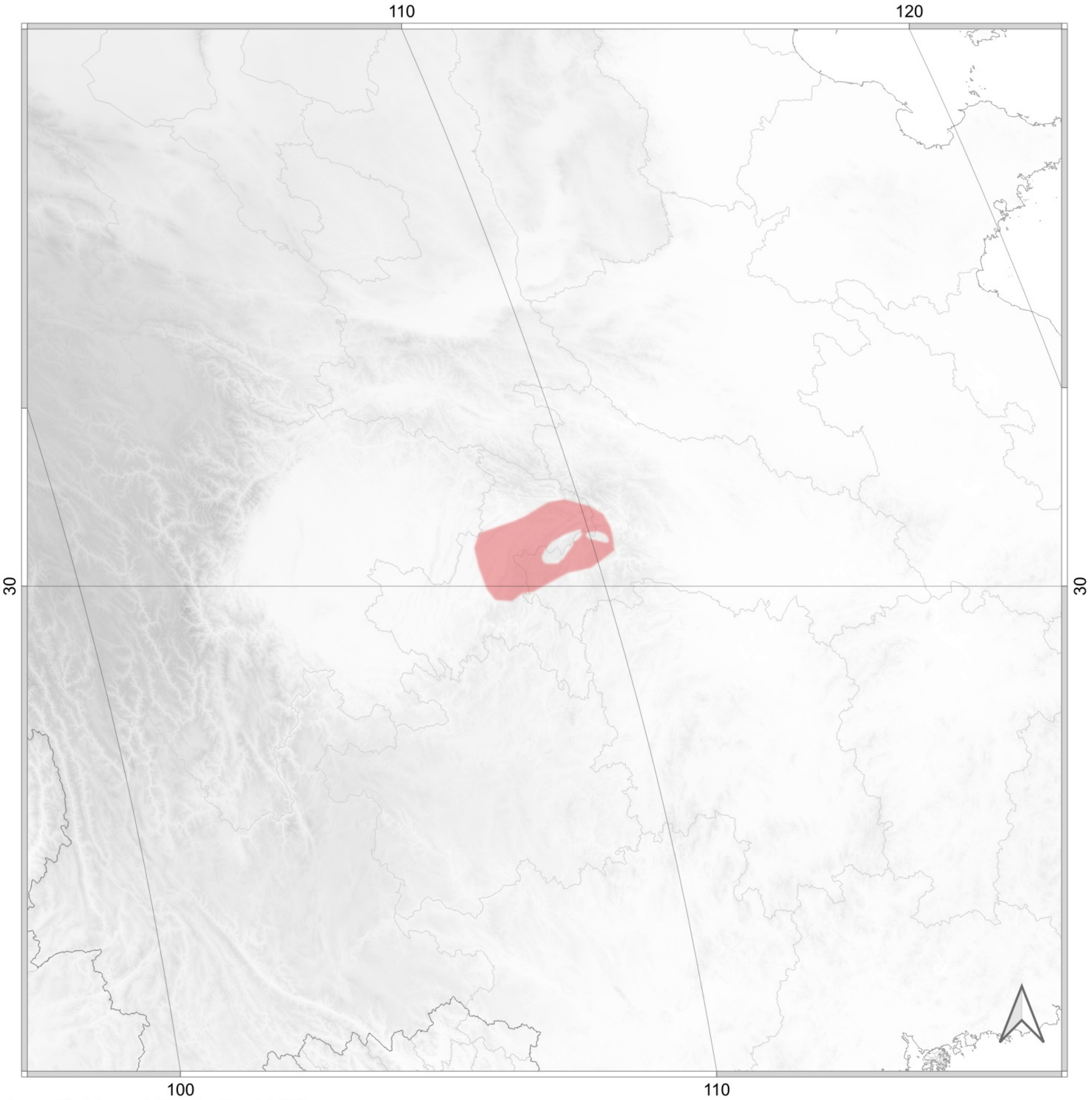

Source of the data : see details in Fragnière et al., 2021

Ulmus pseudopropinqua

Wang & Li

Ill. Fl. Lign. Pl. N.-E. China 231, 561 (1955)

ULMACEAE

IUCN Red list status : ne
*
habitat-ecology : no information
*
climate - Köppen classification : Dwa
*
indicative altitudinal range : 100 - 500 m
*
min. latitude : 45.4, max. latitude : 46.7,
min. longitude : 126.3, max. longitude : 127.8

poorly understood species should be reinvestigated. min+ max elevation estimated from distribution

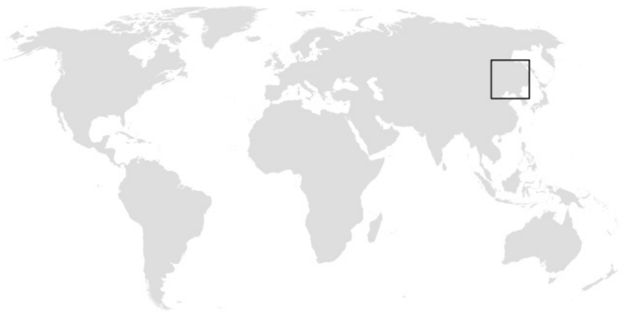

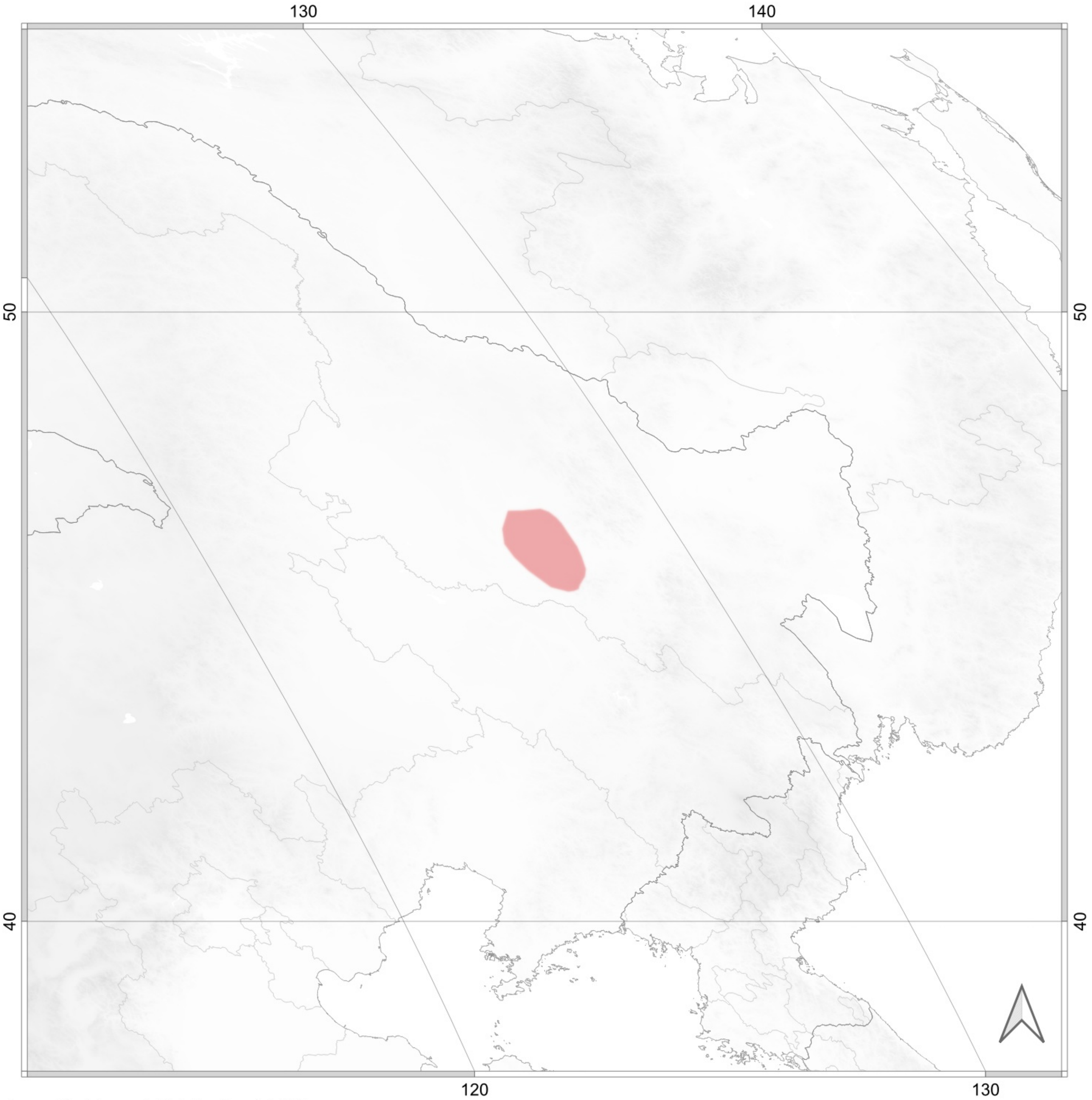

Source of the data : see details in Fragnière et al., 2021

Ulmus pumila ULMACEAE

L.

Sp. Pl. 1: 226 (1753)

IUCN Red list status : LC
*
habitat-ecology : extremely cold resistant, very tolerant to drought, light demanding
*
climate - Köppen classification : BSk, Dwa, Dwb, Dwc, BWk, Cwa, Cwb, Cfa, (Dfc)
*
indicative altitudinal range : 0 - 2500 m
*
min. latitude : 22.9, max. latitude : 54.6,
min. longitude : 71.7, max. longitude : 136.6

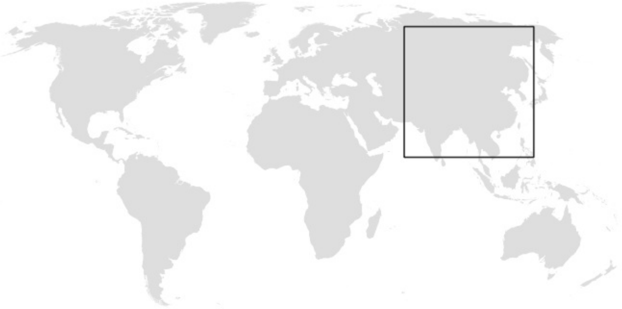

widely planted, natural distribution difficult to know

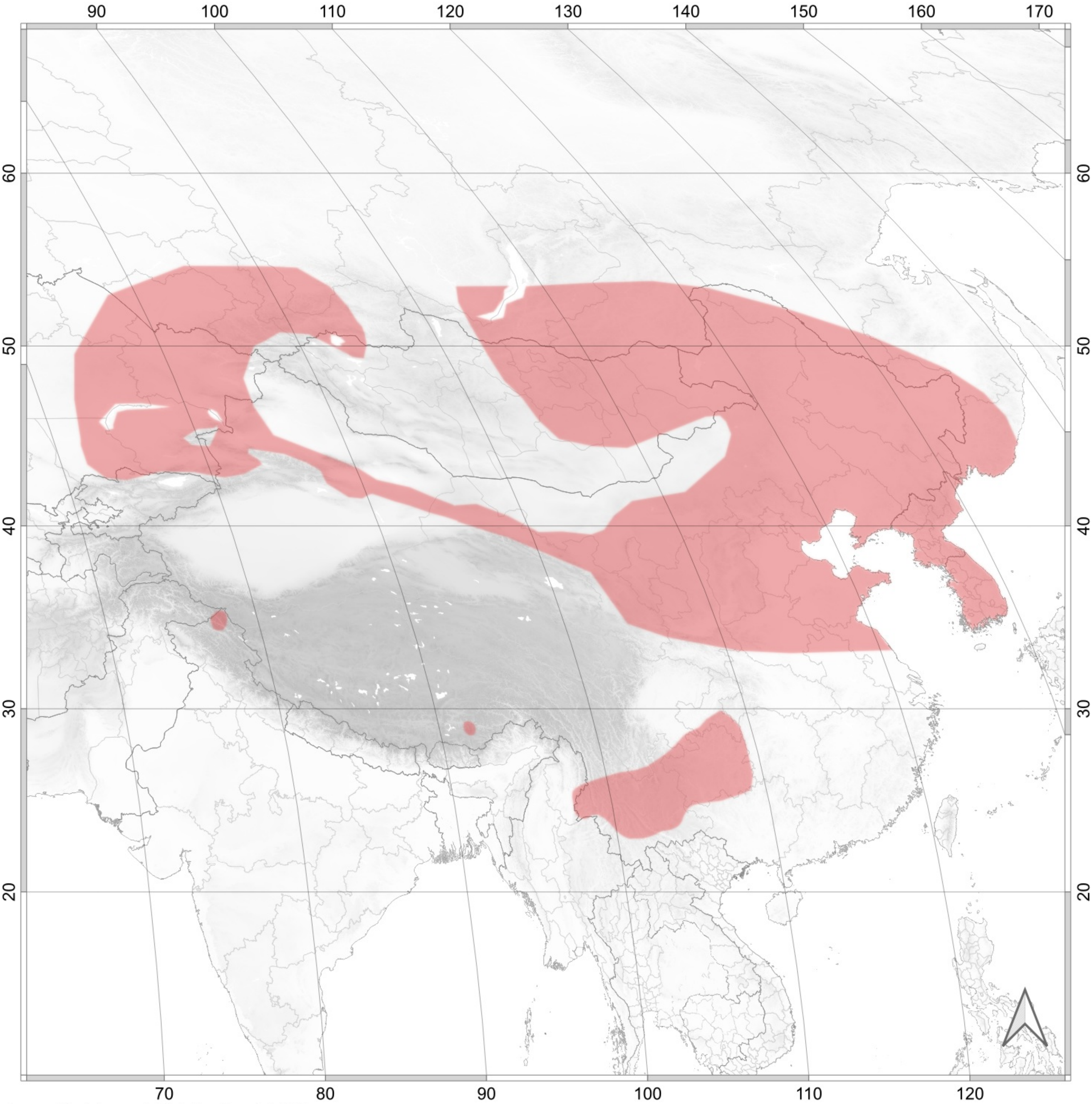

Source of the data : see details in Fragnière et al., 2021

Ulmus rubra

ULMACEAE

Muhl.

Trans. Amer. Philos. Soc. 3: 165 (1793)

IUCN Red list status : LC
*
habitat-ecology : lower slopes, alluvial flood plains, stream banks, riverbanks, and wooded bottom lands
*
climate - Köppen classification : Cfa, Dfa, (Dfb)
*
indicative altitudinal range : 0 - 900 m
*
min. latitude : 28.7, max. latitude : 47.5,
min. longitude : -99.3, max. longitude : -71

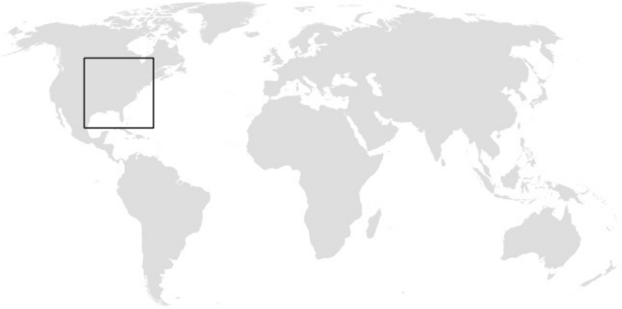

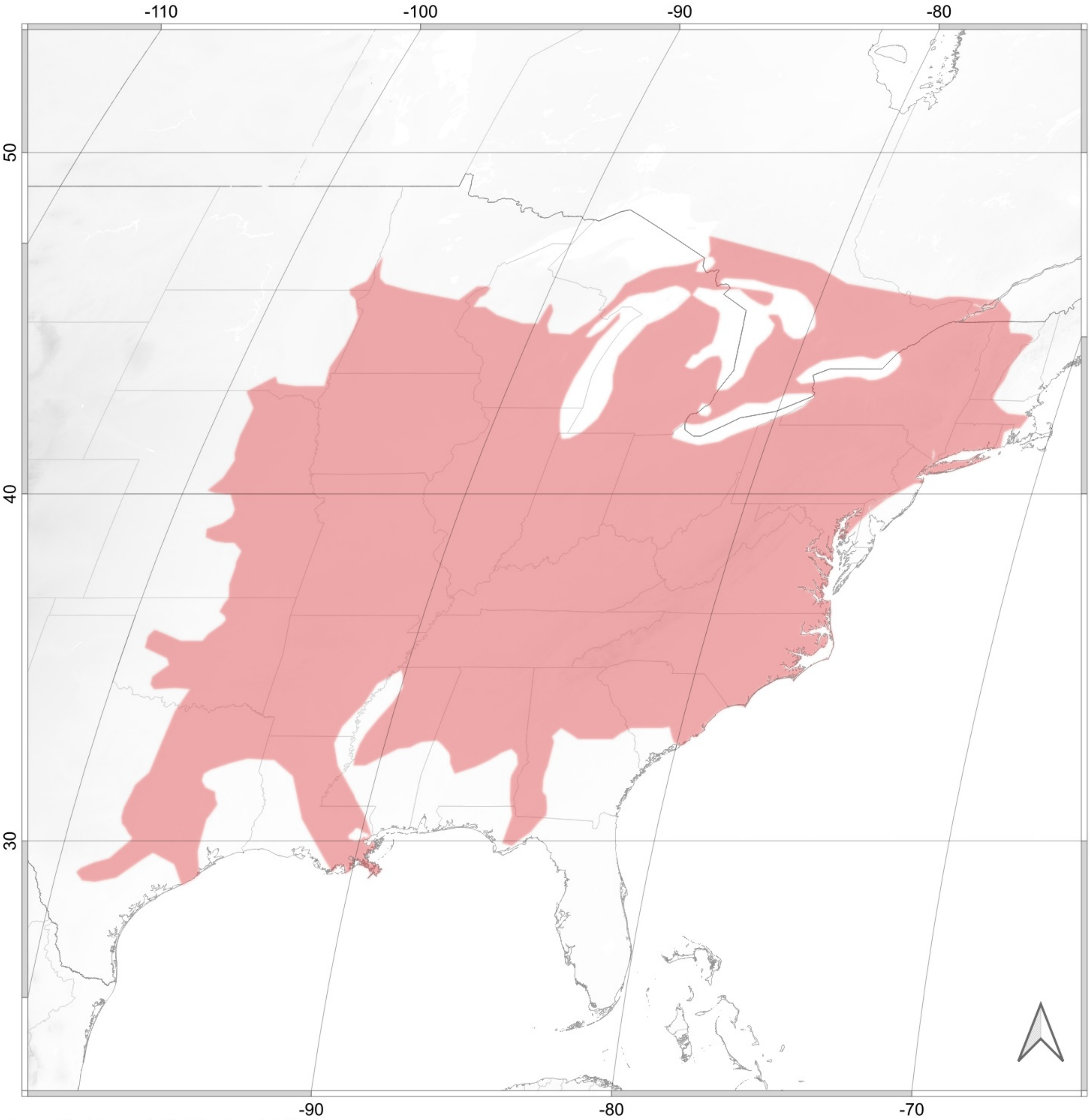

Source of the data : see details in Fragnière et al., 2021

Ulmus serotina

Sarg.

Bot. Gaz. 27: 92 (1899)

ULMACEAE

IUCN Red list status : LC
*
habitat-ecology : limestone bluffs, stream sides, rich woods
*
climate - Köppen classification : Cfa
*
indicative altitudinal range : 0 - 400 m
*
min. latitude : 32.6, max. latitude : 36.8,
min. longitude : -95.3, max. longitude : -85.1

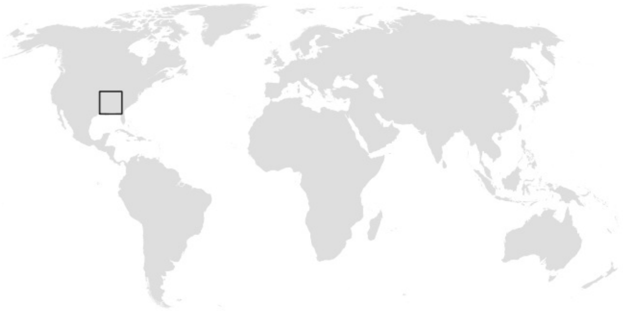

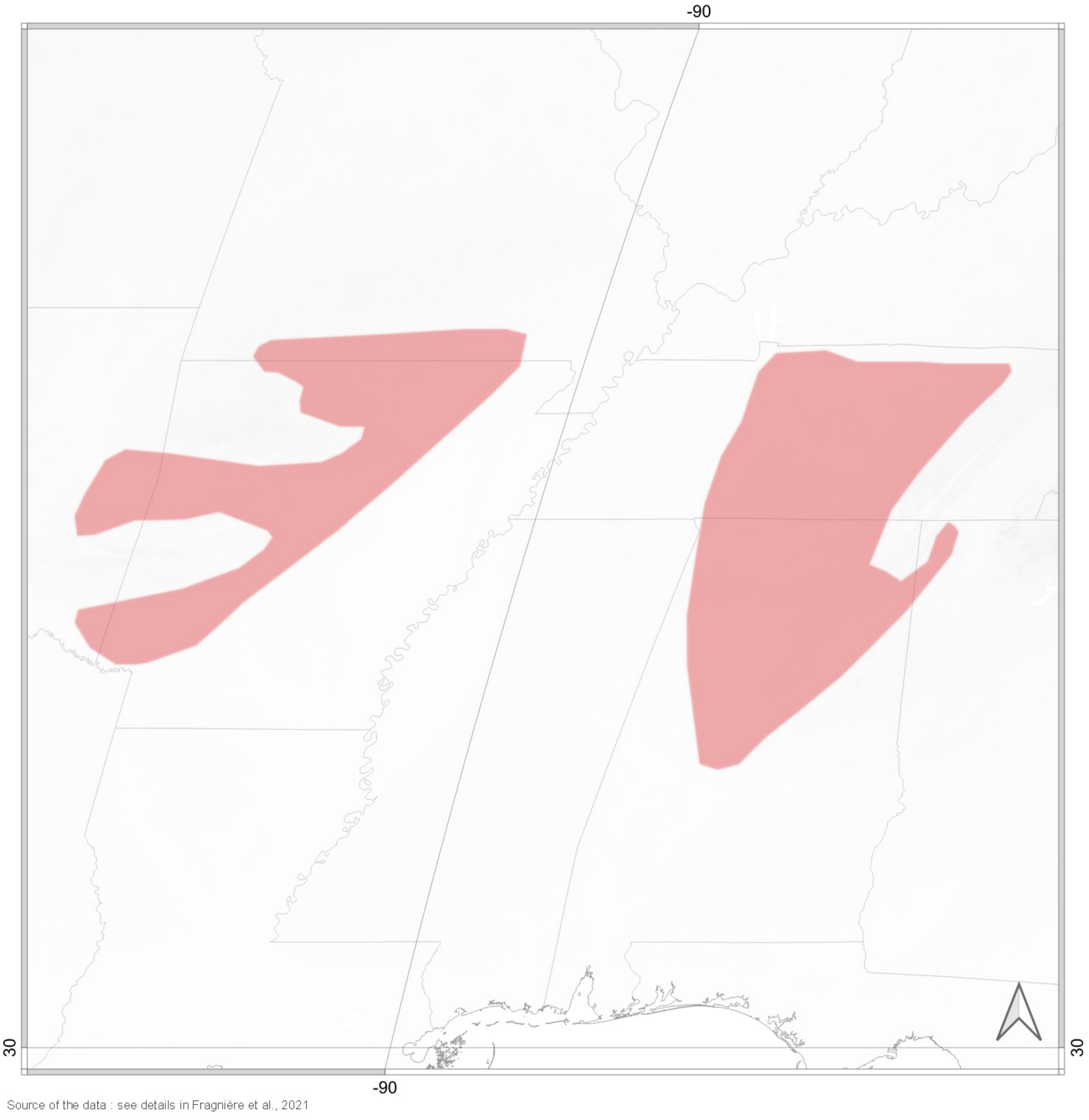

Source of the data : see details in Fragnière et al., 2021

Ulmus szechuanica

W.P. Fang

Commem. Vol. 22 (1947)

ULMACEAE

IUCN Red list status : LC
*
habitat-ecology : no information
*
climate - Köppen classification : Cfa, (Cwa)
*
indicative altitudinal range : 0 - 1100 m
*
min. latitude : 27.1, max. latitude : 32,
min. longitude : 104.1, max. longitude : 120.6

min+ max elevation estimated from distribution

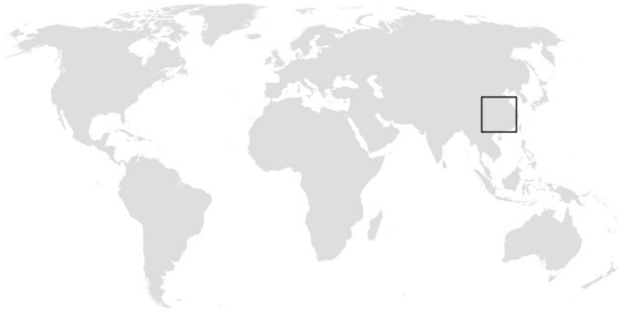

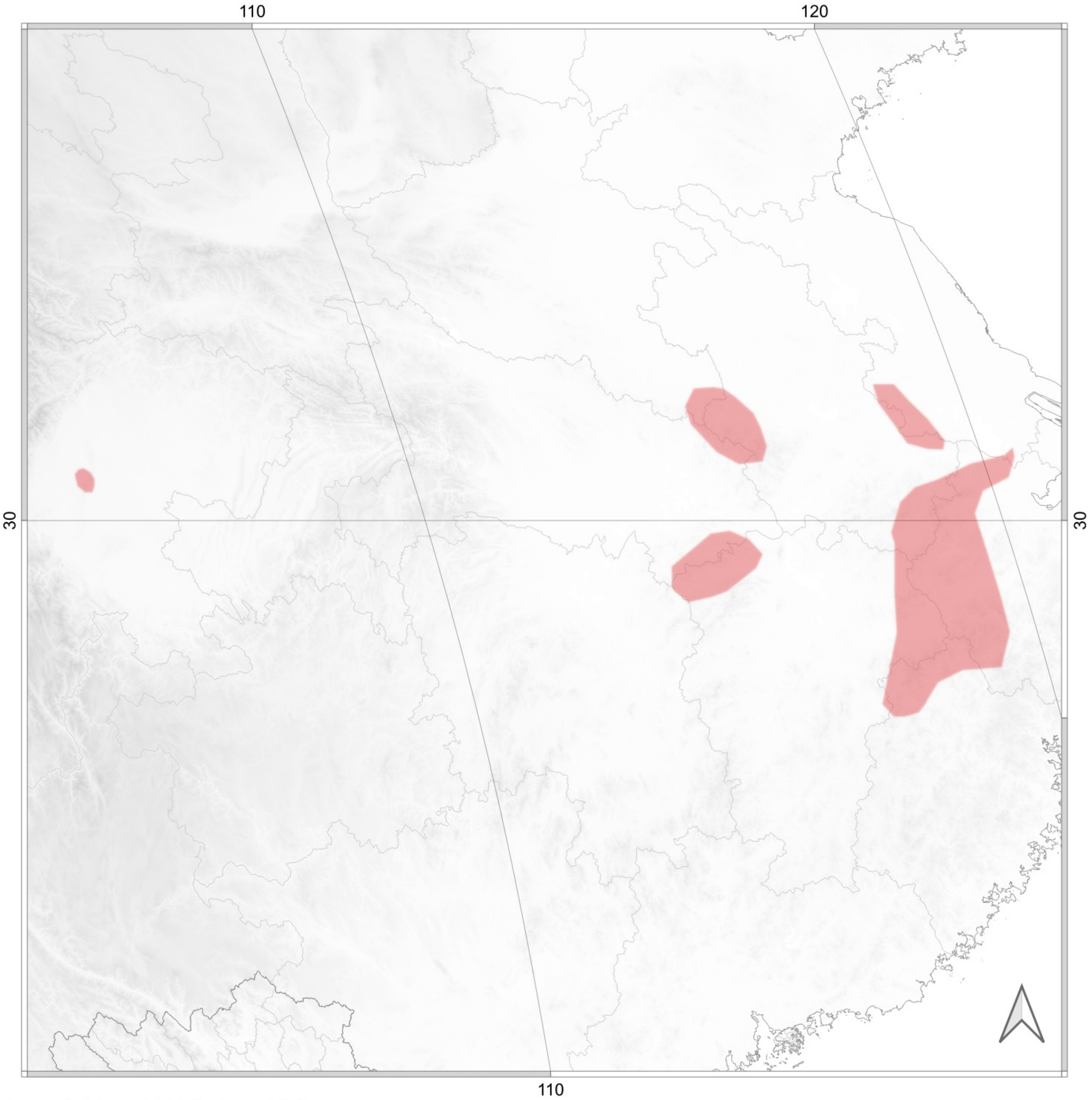

Source of the data : see details in Fragnière et al., 2021

Ulmus thomasii

Sarg.

Silva (Sargent) 14: 102 (1902)

ULMACEAE

IUCN Red list status : LC
*
habitat-ecology : rocky slopes, limestone outcrops, rich woods, flood plains, stream banks
*
climate - Köppen classification : Dfa, Dfb, Cfa
*
indicative altitudinal range : 30 - 900 m
*
min. latitude : 35, max. latitude : 48,
min. longitude : -96.9, max. longitude : -72.9

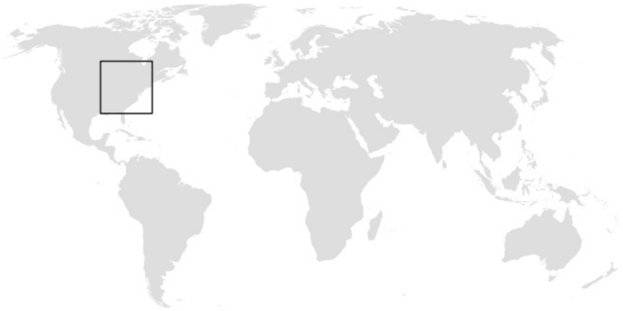

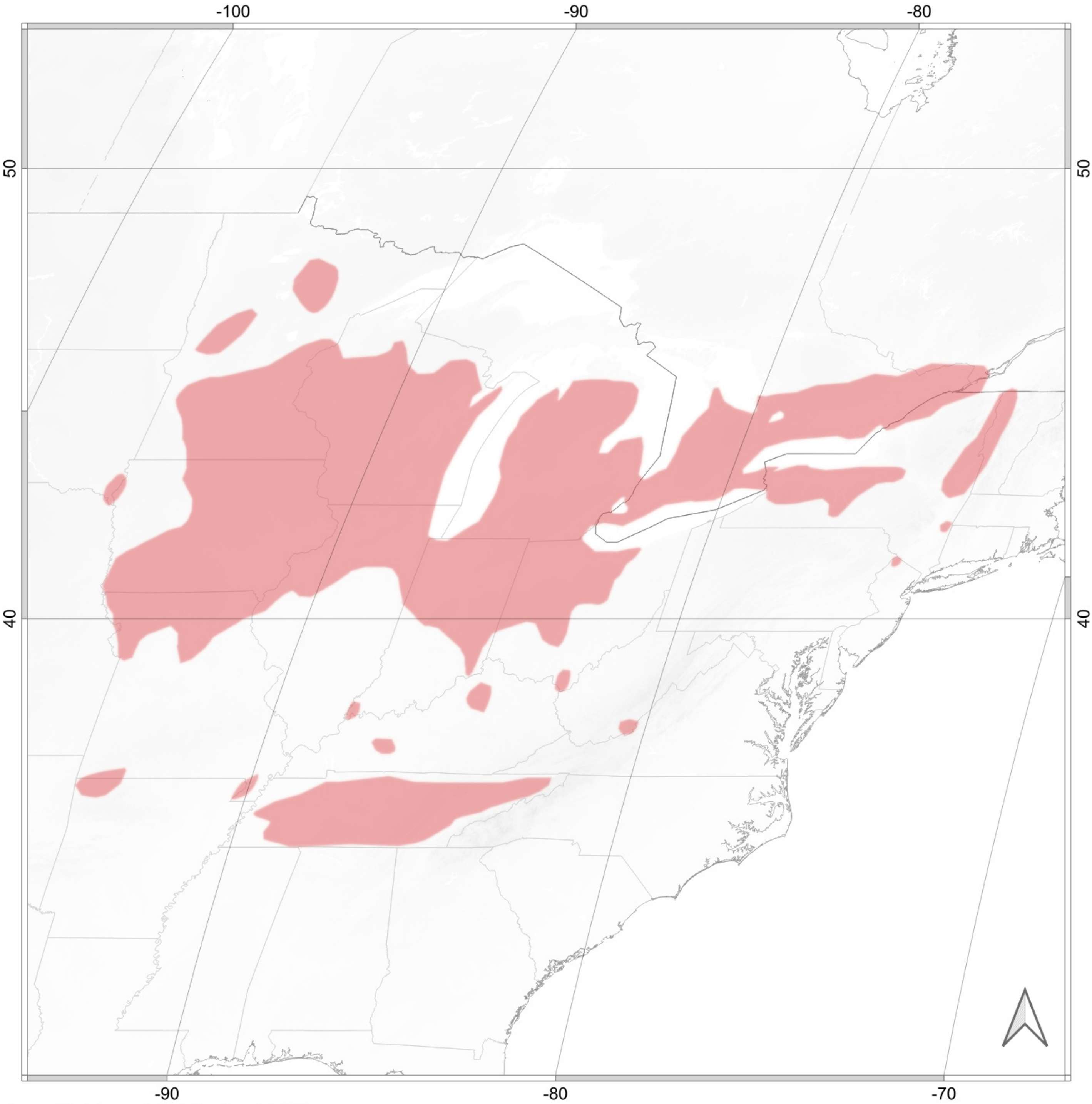

Source of the data : see details in Fragnière et al., 2021

Ulmus uyematsui

ULMACEAE

Hayata

Icon. Pl. Formosan. 3: 174 (1913)

IUCN Red list status : ne
*
habitat-ecology : forests in mountains
*
climate - Köppen classification : Cfb, Cwb, (Cfa), (Cwa)
*
indicative altitudinal range : 800 - 2500 m
*
min. latitude : 22.3, max. latitude : 25.2,
min. longitude : 120.6, max. longitude : 121.8

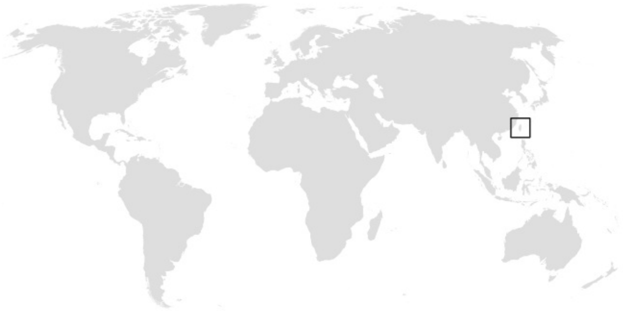

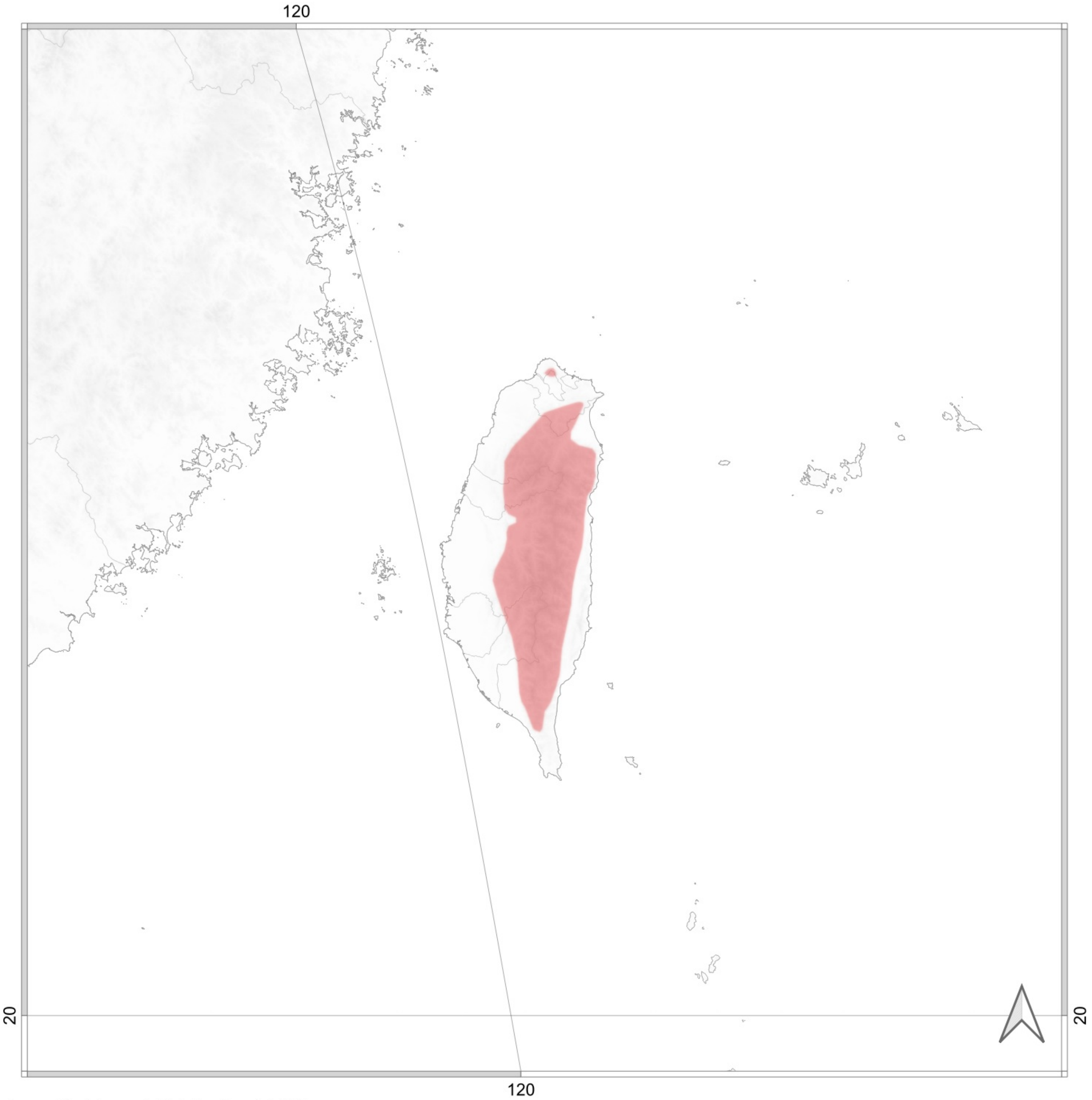

Source of the data : see details in Fragnière et al., 2021

Ulmus villosa

ULMACEAE

Brandis ex Gamble

Man. Ind. Timb. (ed. 2). 628 (1902)

IUCN Red list status : ne
*
habitat-ecology : no information
*
climate - Köppen classification : Cwa, Cwb, Cfa, Csa, BSk, Dfb, Dsb
*
indicative altitudinal range : 1200 - 2700 m
*
min. latitude : 31.1, max. latitude : 35.4,
min. longitude : 69, max. longitude : 77.9

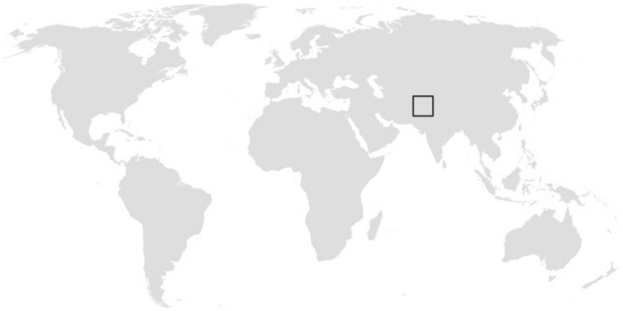

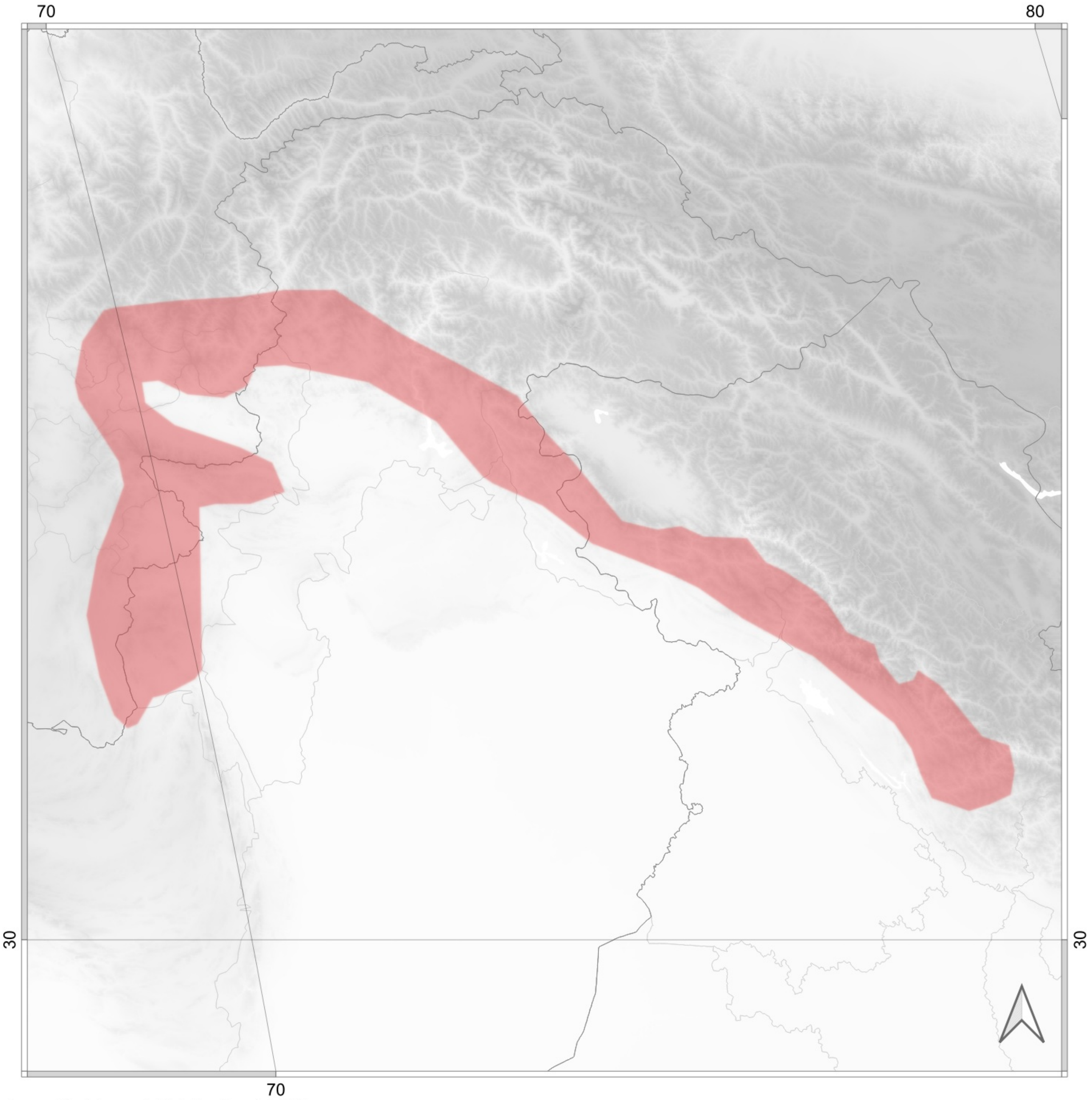

Source of the data : see details in Fragnière et al., 2021

Ulmus wallichiana

ULMACEAE

Planch.

Ann. Sci. Nat., Bot. sér. 3, 10: 277 (1848)

IUCN Red list status : VU
*
habitat-ecology : moist ravines
*
climate - Köppen classification : Cwa, Cwb, Csa, Cfa, Dfb, (Dsb)
*
indicative altitudinal range : 800 - 3000 m
*
min. latitude : 28.2, max. latitude : 35.4,
min. longitude : 70.4, max. longitude : 82.6

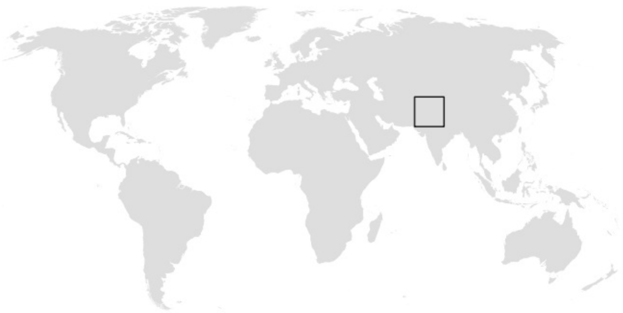

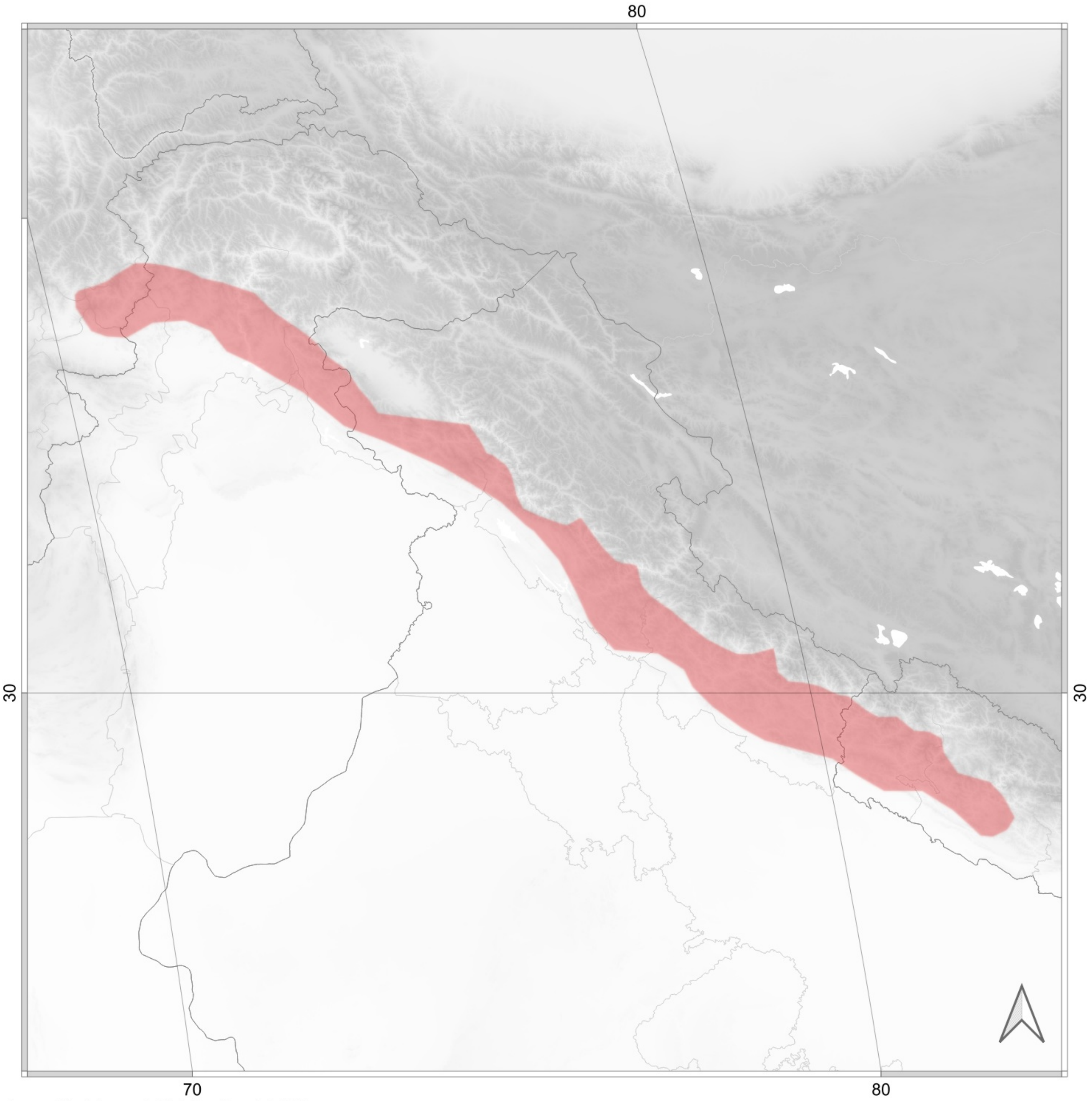

Source of the data : see details in Fragnière et al., 2021

Zelkova abelicea

(Lam.) Boiss.

Fl. Orient. [Boissier] 4(2): 1159 (1879)

ULMACEAE

IUCN Red list status : EN
*
habitat-ecology : rocky areas (inland cliffs, mountain peaks), shrublands, forests
*
climate - Köppen classification : Csb
*
indicative altitudinal range : 900 - 1800 m
*
min. latitude : 35, max. latitude : 35.4,
min. longitude : 23.8, max. longitude : 25.9

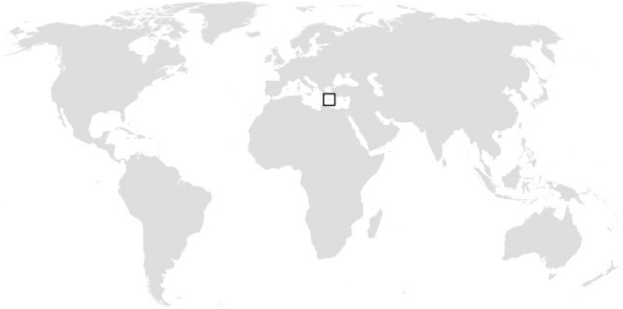

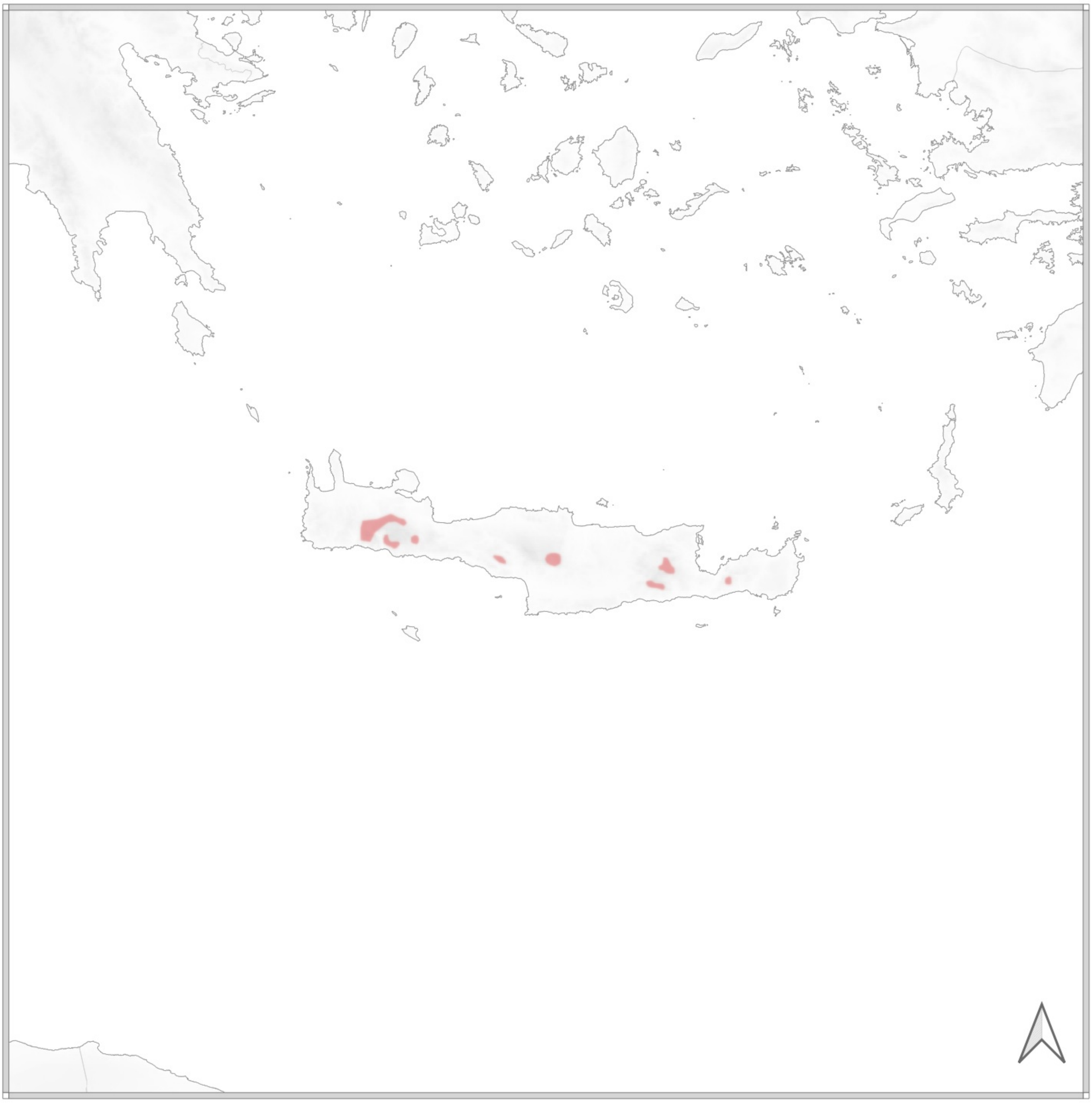

Source of the data : see details in Fragnière et al., 2021

Zelkova carpinifolia

(Pall.) K. Koch

Linnaea 22: 598 (1849)

ULMACEAE

IUCN Red list status : VU
*
habitat-ecology : moist, humus-rich soils but not tolerating waterlogged or swampy conditions
*
climate - Köppen classification : Csa, BSk, Cfa, Cfb, (Dfa)
*
indicative altitudinal range : 100 - 1500 m
*
min. latitude : 35.1, max. latitude : 43,
min. longitude : 39.9, max. longitude : 56.1

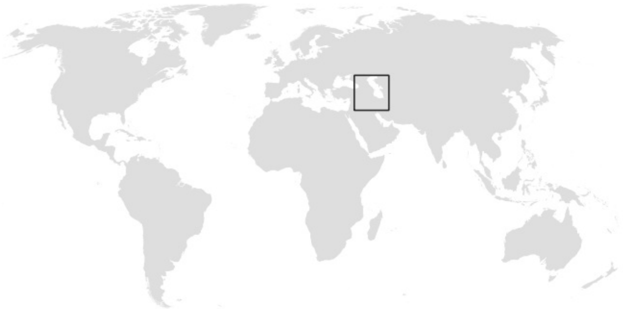

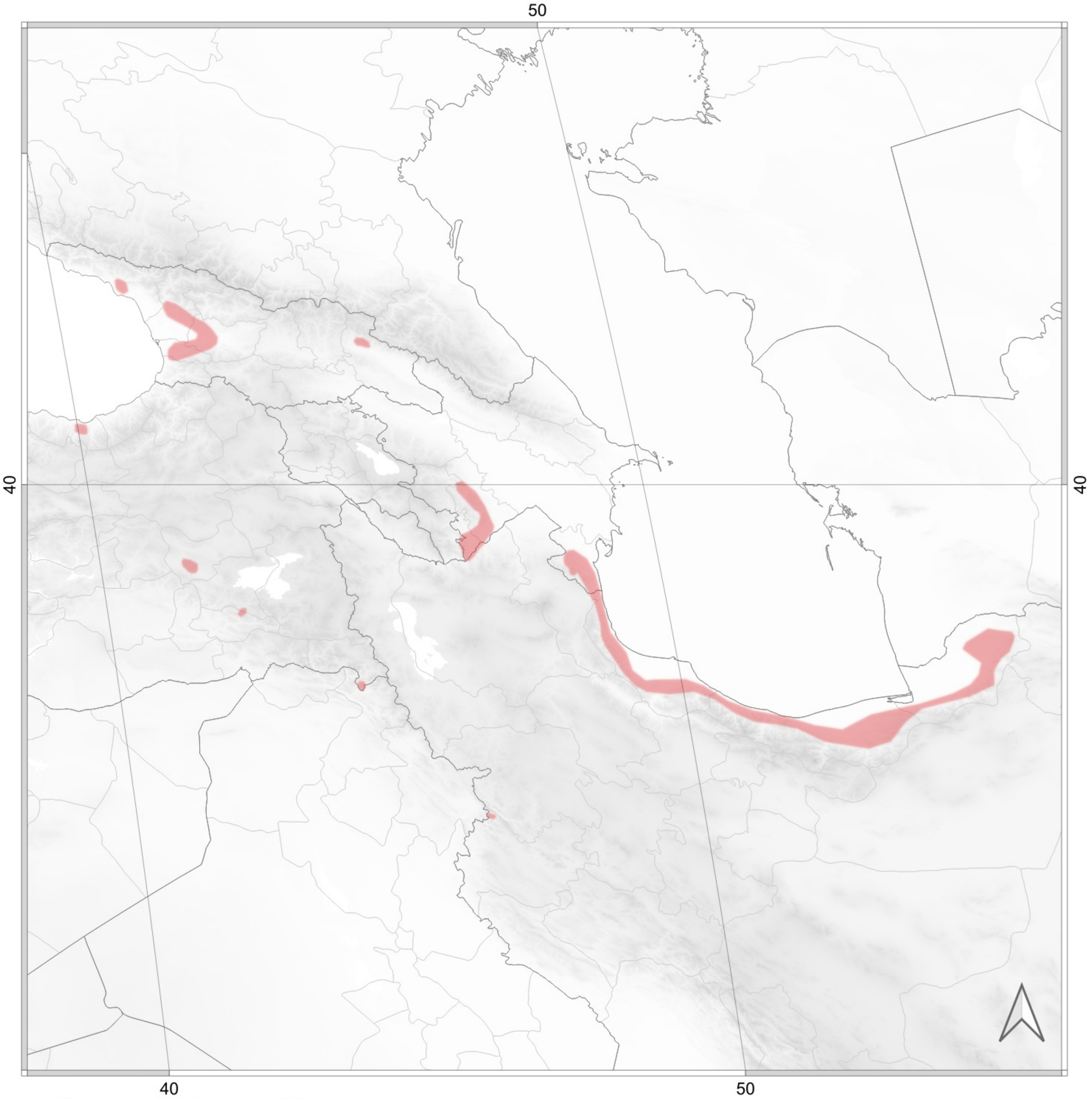

Source of the data : see details in Fragnière et al., 2021

Zelkova schneideriana

ULMACEAE

Hand.-Mazz.

Symb. Sin. Pt. 7, 104 (1929)

IUCN Red list status : VU
*
habitat-ecology : in ravines along streams and rivers, fertile soils
*
climate - Köppen classification : Cwa, Cfa, Cwb, (Dwa)
*
indicative altitudinal range : 0 - 2800 m
*
min. latitude : 22, max. latitude : 35.6,
min. longitude : 97.9, max. longitude : 122.5

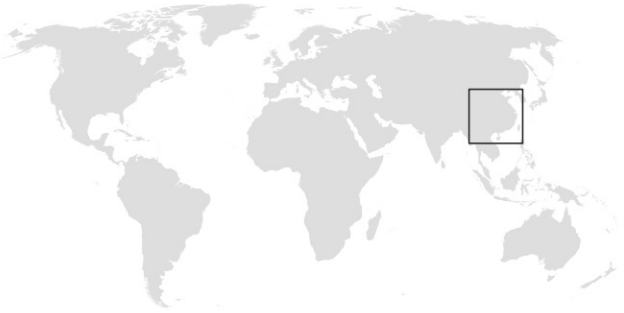

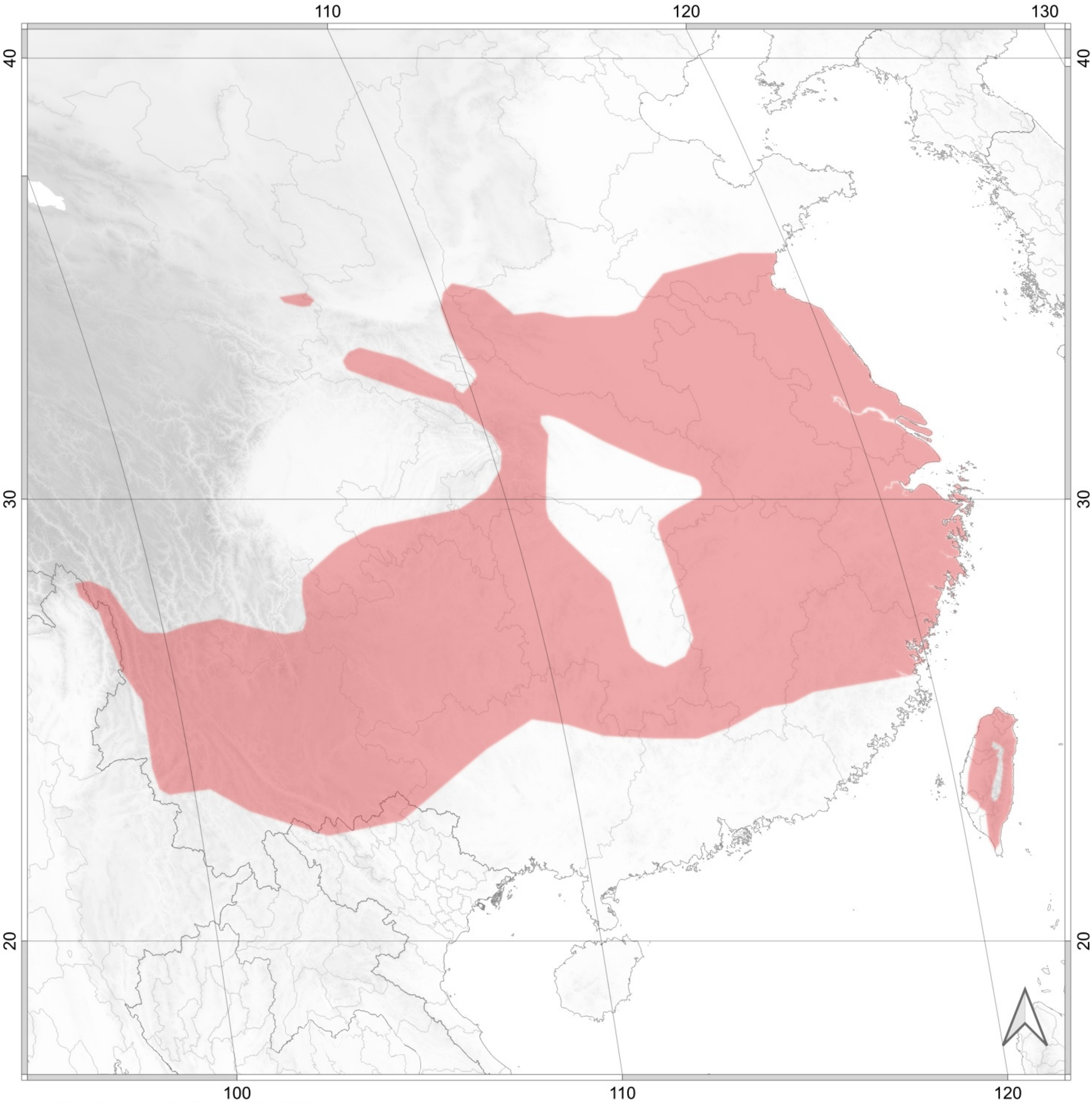

Source of the data : see details in Fragnière et al., 2021

Zelkova serrata

(Thunb.) Makino

Bot. Mag. (Tokyo) 17: 13 (1903)

ULMACEAE

IUCN Red list status : NT
*
habitat-ecology : lowland to mountain forests, riparian habitats, in ravines and on shady slopes
*
climate - Köppen classification : Cfa, Cwa, Dwa, Dfa, Dfb, (Dwb), (Cwb)
*
indicative altitudinal range : 0 - 2000 m
*
min. latitude : 24.1, max. latitude : 43.4,
min. longitude : 104, max. longitude : 142.1

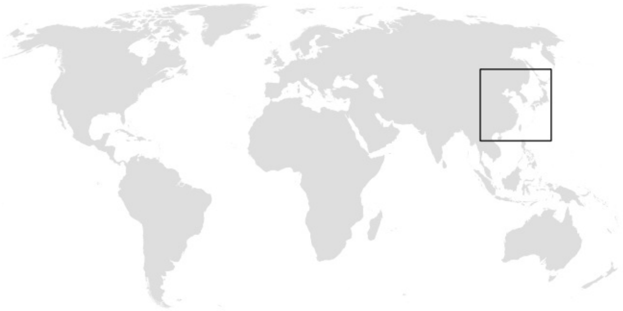

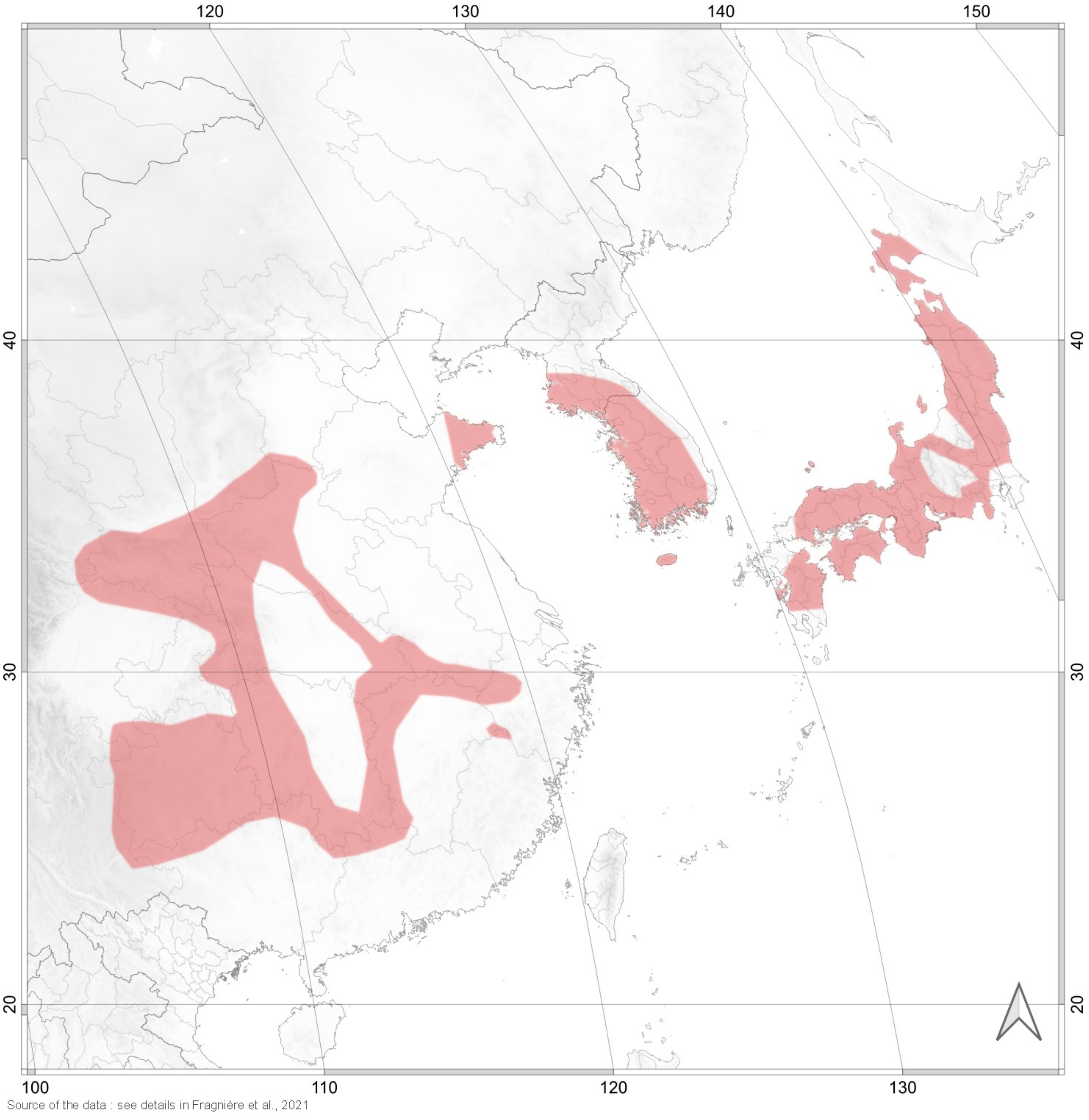

Source of the data : see details in Fragnière et al., 2021

Zelkova sicula

Di Pasq., Garfi & Quézel

Biocosme Mésogéen 8(4)-9(1): 403, 406 (1992)

ULMACEAE

IUCN Red list status : CR
*
habitat-ecology : shrublands, wetlands
*
climate - Köppen classification : Csa
*
indicative altitudinal range : 320 - 530 m
*
min. latitude : 37.2, max. latitude : 37.2,
min. longitude : 14.9, max. longitude : 15.1

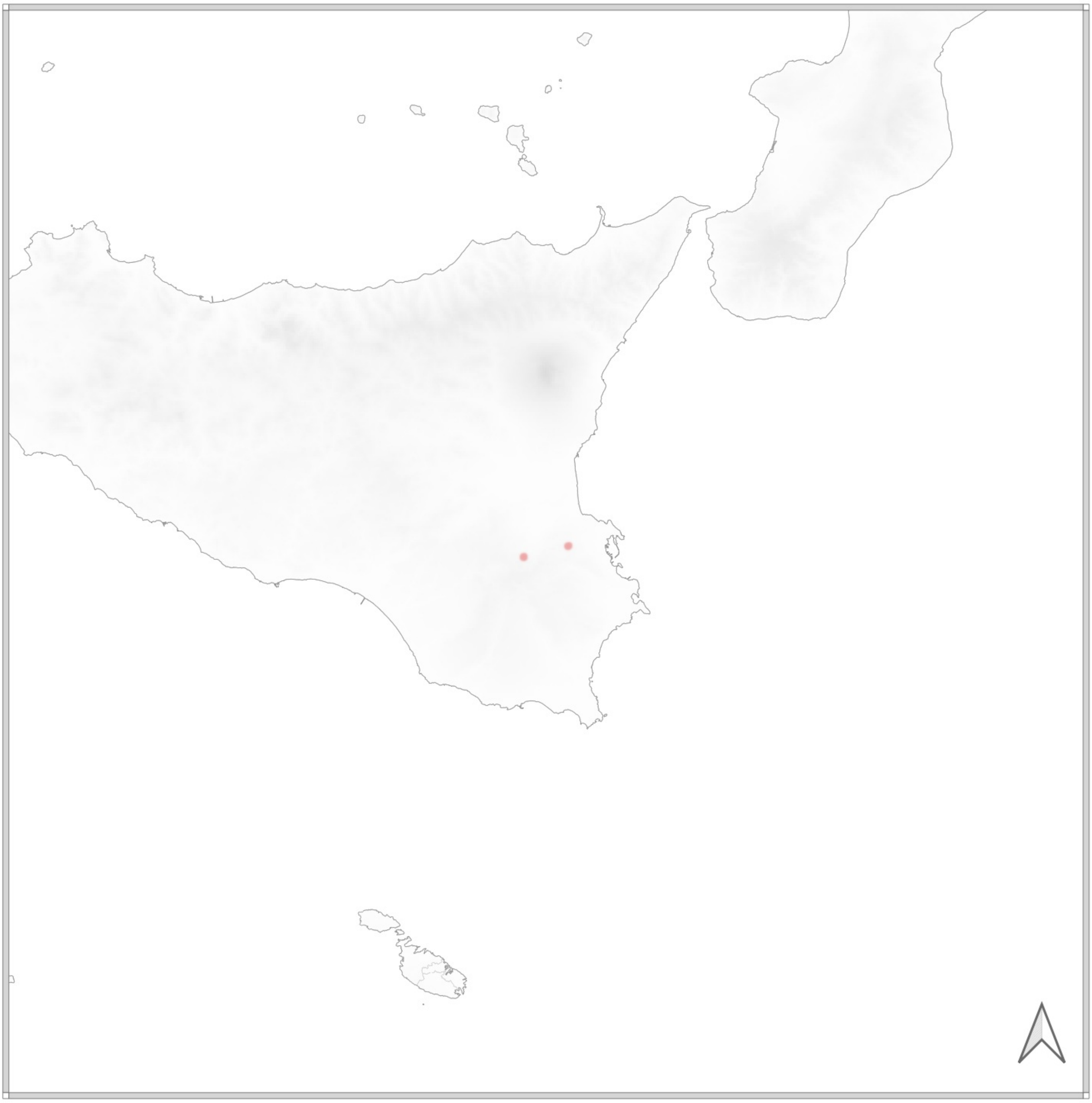

Source of the data : see details in Fragnière et al., 2021

Zelkova sinica

C.K.Schneid.

Pl. Wilson. (Sargent) 3(2): 286 (1916)

ULMACEAE

IUCN Red list status : VU
*
habitat-ecology : humid, subtropical deciduous forests, in valleys along rivers
*
climate - Köppen classification : Cwa, Cfa, Dwa, (Dwb)
*
indicative altitudinal range : 100 - 2500 m
*
min. latitude : 24.6, max. latitude : 37.8,
min. longitude : 104.1, max. longitude : 119.1

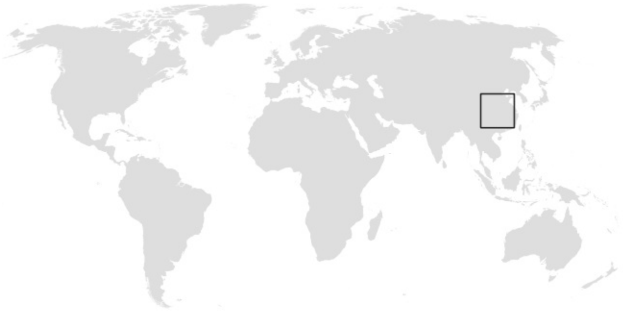

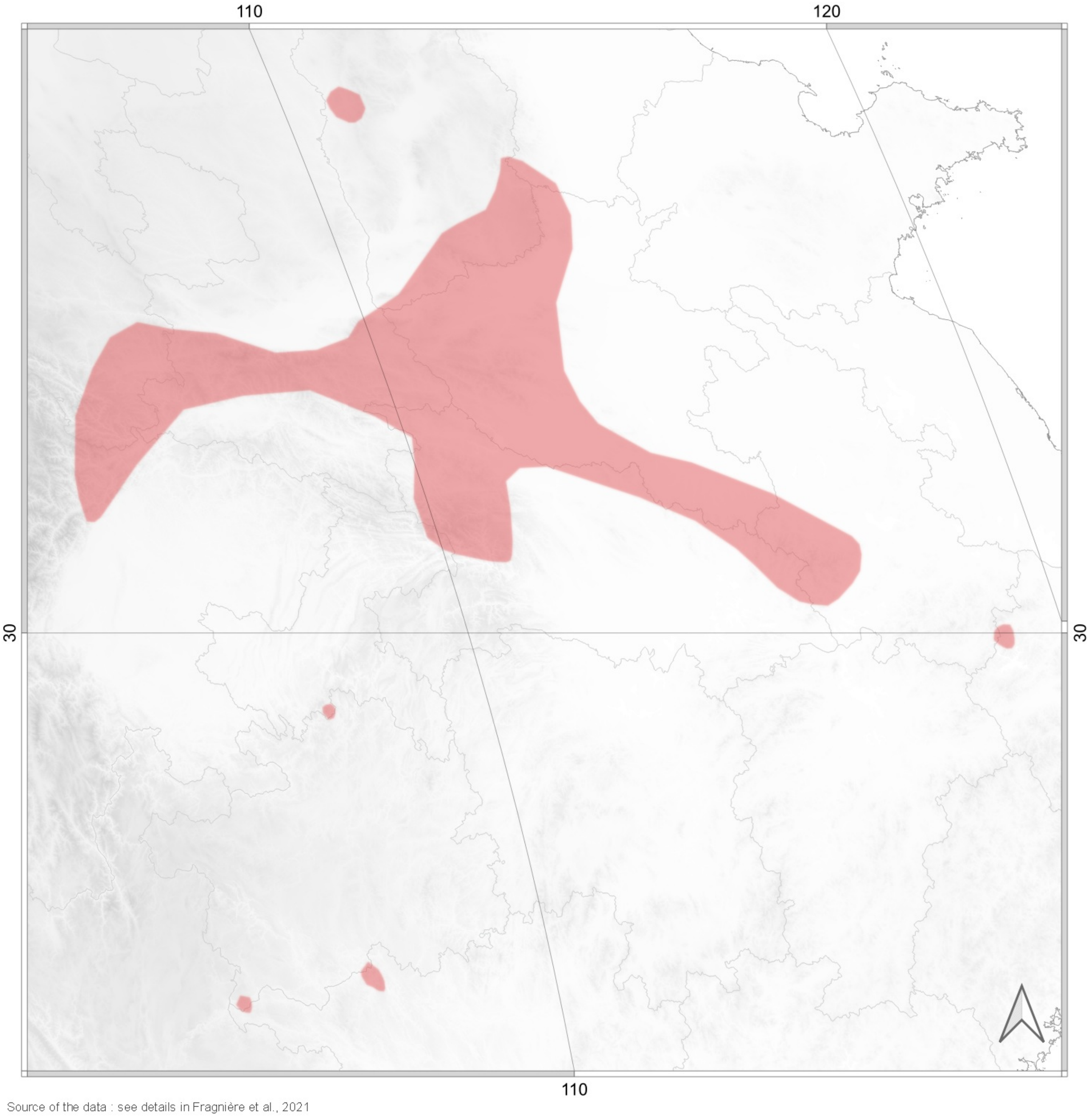

Source of the data : see details in Fragnière et al., 2021
