# Supplementary material for: Biogeographic Overview of Ulmaceae: Diversity, Distribution, Ecological Preferences, and Conservation Status
Source: Plants (Basel). 2021 May 31;10(6):1111. doi: 10.3390/plants10061111 (PMC8227750; doi:10.3390/plants10061111)
Supplement: Supplementary file 1 [file plants-10-01111-s001.zip › Supplementary file S3 List of bioclimatic variables.pdf]

Biogeographic Overview of Ulmaceae: Diversity, Distribution, Ecological Preferences, and Conservation Status

Fragnière et al.

Supplementary file S3

List of bioclimatic variables

List of the 19 bioclimatic variables used to study the realized macro climatic niche of natural populations for each Ulmaceae species. The data of were downloaded from WorldClim version 2.1 (Fick and Hijmans, 2017). For more details and explanations about the different variables, see also (O'Donnell and Ignizio, 2012).

1. Annual Mean Temperature
2. Mean Diurnal Range (Mean of monthly (max temp - min temp))
3. Isothermality (BIO2/BIO7) (×100)
4. Temperature Seasonality (standard deviation ×100)
5. Max Temperature of Warmest Month
6. Min Temperature of Coldest Month
7. Temperature Annual Range (BIO5-BIO6)
8. Mean Temperature of Wettest Quarter
9. Mean Temperature of Driest Quarter
10. Mean Temperature of Warmest Quarter
11. Mean Temperature of Coldest Quarter
12. Annual Precipitation
13. Precipitation of Wettest Month
14. Precipitation of Driest Month
15. Precipitation Seasonality (Coefficient of Variation)
16. Precipitation of Wettest Quarter
17. Precipitation of Driest Quarter
18. Precipitation of Warmest Quarter
19. Precipitation of Coldest Quarter

References

Fick, S. E., and R. J. Hijmans. 2017. WorldClim 2: new 1-km spatial resolution climate surfaces for global land areas. *International Journal of Climatology* 37: 4302–4315.

O'Donnell, M. S., and D. A. Ignizio. 2012. Bioclimatic predictors for supporting ecological applications in the conterminous United States. *US Geological Survey Data Series* 691.
